# Scan-o-matic: High-Resolution Microbial Phenomics at a Massive Scale

**DOI:** 10.1534/g3.116.032342

**Published:** 2016-06-30

**Authors:** Martin Zackrisson, Johan Hallin, Lars-Göran Ottosson, Peter Dahl, Esteban Fernandez-Parada, Erik Ländström, Luciano Fernandez-Ricaud, Petra Kaferle, Andreas Skyman, Simon Stenberg, Stig Omholt, Uroš Petrovič, Jonas Warringer, Anders Blomberg

**Affiliations:** * Department of Chemistry and Molecular Biology, University of Gothenburg, 40530, Sweden; † IRCAN, CNRS UMR 6267, INSERM U998, University of Nice, 06107, France; ‡ Department of Marine Sciences, University of Gothenburg, 40530, Sweden; § Department of Molecular and Biomedical Sciences, Jožef Stefan Institute, SI-1000 Ljubljana, Slovenia; ** Department of Earth and Space Sciences, Chalmers University of Technology, SE-412 96 Gothenburg, Sweden; †† Department of Animal and Aquacultural Sciences, Centre for Integrative Genetics (CIGENE), Norwegian University of Life Sciences (UMB), 1432 Ås, Norway; ‡‡ Department of Biotechnology, Faculty of Natural Sciences and Technology, NTNU Norwegian University of Science and Technology, N-7491 Trondheim, Norway

**Keywords:** phenomics, microbiology, genetics, high throughput, screening

## Abstract

The capacity to map traits over large cohorts of individuals—phenomics—lags far behind the explosive development in genomics. For microbes, the estimation of growth is the key phenotype because of its link to fitness. We introduce an automated microbial phenomics framework that delivers accurate, precise, and highly resolved growth phenotypes at an unprecedented scale. Advancements were achieved through the introduction of transmissive scanning hardware and software technology, frequent acquisition of exact colony population size measurements, extraction of population growth rates from growth curves, and removal of spatial bias by reference-surface normalization. Our prototype arrangement automatically records and analyzes close to 100,000 growth curves in parallel. We demonstrate the power of the approach by extending and nuancing the known salt-defense biology in baker’s yeast. The introduced framework represents a major advance in microbial phenomics by providing high-quality data for extensive cohorts of individuals and generating well-populated and standardized phenomics databases

While our ability to detect and manipulate genetic variation has improved tremendously (Koboldt *et al.* 2013; Mardis 2013), our capacity to rapidly and accurately map the phenotypic effects of this variation—phenomics—has not made comparable gains (Houle *et al.* 2010). This is certainly true for microbes, where estimates of fitness components define the key entrance point when searching for the concerted effects of causative genetic variation. Phenomics requires the availability of large cohorts of populations of genetically diverse strains, with the model yeast *Saccharomyces cerevisiae* leading the way. Collections of artificial gene constructs, *e.g.*, single gene knockout or overexpression strains, have facilitated gene function analysis in lab strains (Giaever *et al.* 2002; Sopko *et al.* 2006; Li *et al.* 2011). Large crosses of natural, industrial, or clinical isolates allow dissection of the genotype–phenotype relationship in wider perspectives (Cubillos *et al.* 2011, 2013; Parts *et al.* 2011; Bloom *et al.* 2013). As the majority of genetic or environmental effects on net fitness are modest in size (Thatcher *et al.* 1998; Wagner 2000), this demands access to a highly accurate measurement methodology capable of capturing subtle differences in growth phenotypes.

An array of technologies has been applied in microbial growth phenomics (Blomberg 2011). Time-resolved analysis of microcolonies estimates within-population variations in growth (Levy *et al.* 2012) but is low throughput for studies on differences between strains. Miniaturization of well-mixed liquid cultures allows automated recording of high frequency time series and very accurate measurement of population growth curves from which growth variables can be extracted (Warringer and Blomberg 2003, 2014). However, its associated costs in time and manpower are too high to allow massive scale-up in the number of strains analyzed. Tagging of strains with DNA sequence barcodes and monitoring tag frequencies in complex strain mixtures allows very high throughput, but at the cost of less accurate and precise measurements, the potential for scoring strain-strain interactions and a costly initial investment in the tagging of strains to be phenotyped (Giaever *et al.* 2002; Winzeler *et al.* 1999). Better cost-efficiency can be achieved using ordered arrays of microbes that are cultivated on solid media as colonies whose area is estimated using cameras (Costanzo *et al.* 2010; Kvitek *et al.* 2008; Bean *et al.* 2014; Lawless *et al.* 2010). However, the environment can rarely be maintained constant across plates. This leads to large spatial biases, especially at plate margins. Spatial bias correction is a formidable challenge (Baryshnikova *et al.* 2010) because colonies exchange nutrients and toxins with the local environment and thereby affect the growth of neighboring colonies. Accentuating problems, 3-dimensional (3D) colony population size is typically approximated by measuring the often poorly correlated 2-dimensional (2D) colony area (Pipe and Grimson 2008). Furthermore, growth is mostly estimated from a single endpoint measure of colony area (Tong *et al.* 2001; Baryshnikova *et al.* 2010; Collins *et al.* 2006). As any particular end point can be reached via an endless number of very different growth paths, this often confounds conclusions by comparing colonies in different physiological states (Figure 1A; conceptual figure). Cultivation on agar has also involved the recording of low frequency time series, followed by image analysis and the fitting of relatively sparse data to models with preconceived definitions of what growth should look like (Hartman *et al.* 2015; Banks *et al.* 2012; Narayanan *et al.* 2015). Estimation of cell densities can be enhanced after serial dilutions (Hartman *et al.* 2015) but at the cost of extra robotic steps that inevitably will increase errors and decrease throughput.

**Figure 1:**
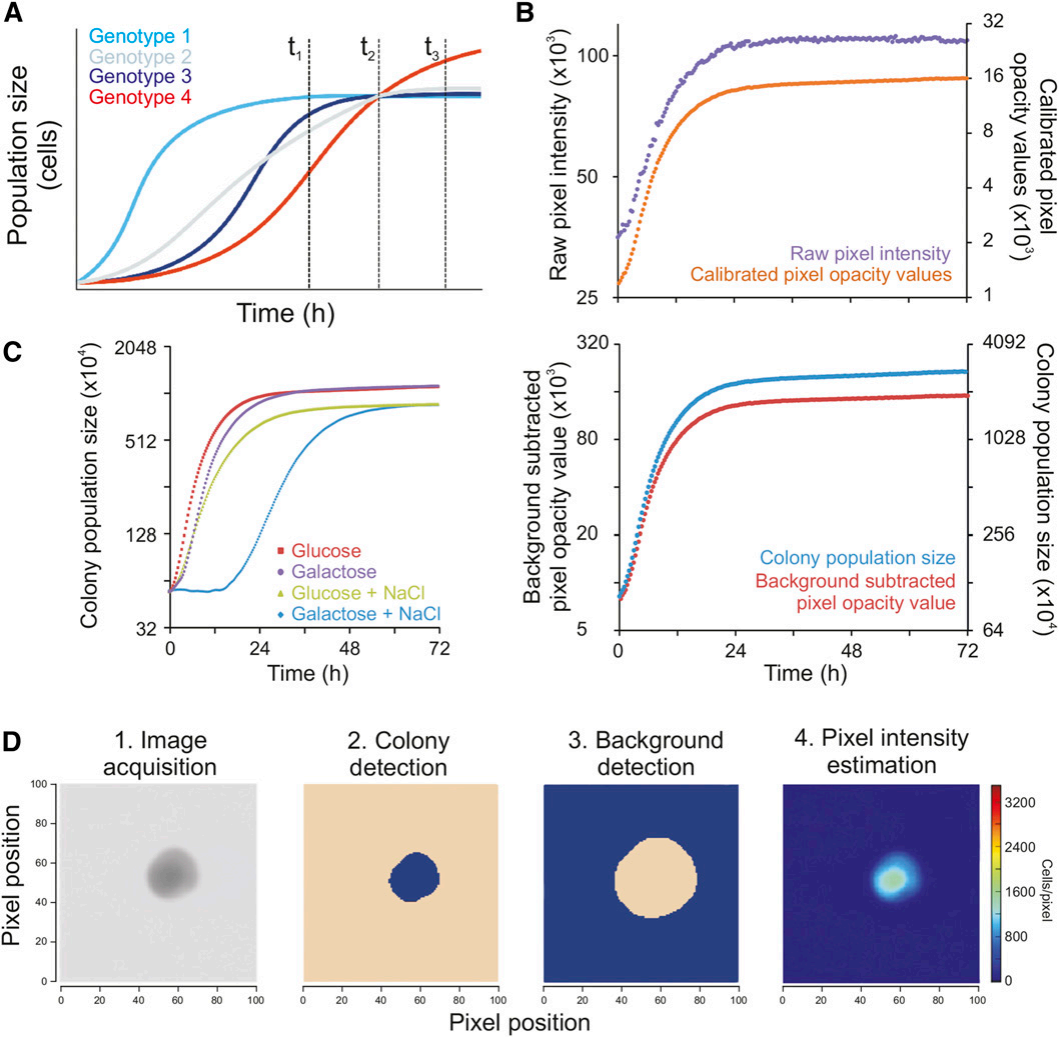
A novel framework for high-resolution microbial phenomics. (A) Simulated growth data to exemplify the problem with endpoint analysis given different growth paths. Single time point measures at different stages of growth (vertical broken lines) can provide wildly diverging views of the relative growth performance of strains, alternatively scoring all genotypes as identical (*t_2_*), genotype 1 as superior (*t_1_*), or genotype 4 as superior (*t_3_*). This illustrates the conceptual importance of having access to highly resolved growth curve data. (B) Illustration of the conversion from raw pixel intensities to total population size in cell numbers for a randomly chosen example colony growing on 2% glucose in basal medium. Upper graph: Colony growth curve based on raw pixel intensities (left axis; lilac) and calibrated pixel opacity values (right axis; orange). Lower graph: Colony growth curve based on background subtracted pixel opacity values (left *y*-axis; red) and cell counts/population size (right *y*-axis; blue). (C) Colony population growth curves obtained by cultivating genetically identical WT (BY4743) colonies in four environmental contexts (conditions indicated) after having shared identical preculture media and measuring colony population size in 20 min intervals. *y*-axis is on log_2_ scale. (D) Colony population size is extracted from raw images in a multi-step procedure, proceeding from 1. raw image, to 2. probable colony (blob; blue) detection and segmentation, 3. local background definition (blue) with a safety margin to colony, and 4. estimation of cells as pixel intensity as compared to background. Color intensity = 0 (dark blue), 1500 (turquoise) cells per pixel. NaCl, sodium chloride; WT, wild type.

The near-term goal of microbial phenomics is therefore to achieve the technical precision (low random error, noise) and accuracy (low systematic error, bias) of liquid microcultivation in a high-throughput solid media cultivation mode. To reach this goal, the 3D topology of microbial colonies must be captured at high spatial and temporal resolution and reduced to precise and accurate estimates of colony population cell densities, while comprehensively accounting for the spatial and temporal bias that makes different experimental positions and time-points nonequivalent. We present a novel microbial phenomics platform, Scan-o-matic, capable of overcoming these hurdles at a very low cost. We demonstrate its utility on the eukaryotic model organism baker’s yeast, *S. cerevisiae*.

## Materials and Methods

For a complete description of the experimental procedures, please consult Supplemental Material, File S1.

### Physical arrangement of Scan-o-matic

Scan-o-matic is based on high-quality, mass-produced desktop scanners, Epson Perfection V700 PHOTO scanners (Epson Corporation, UK), which are controlled by power-managers, GEMBIRD EnerGenie PowerManager LAN (Gembird Ltd, the Netherlands) (Figure S1, A and B). Images are acquired using SANE (Scanner Access Now Easy) (Mosberger 1998) using transmissive scanning at 600 dpi, 8-bit gray-scale and a scan area extension that captures four plates per image. Plates, without lids, are fixed in custom-made acrylic glass fixtures with orientation markers ensuring software recognition of fixture position (Figure S2). Fixtures are calibrated to each scanner prior to use by a fixture calibration model. Pixel intensities are standardized across instruments using transmissive gray-scale calibration strips, Kodak Professional Q-60 Color Input Target (Kodak Company), each consisting of 20–25 (depending on producer) calibration segments with fixed gray-scale intensities (Figure S3). Converting scanner-generated pixel values to standardized gray-scale values makes calibrated data independent of variation in scanner properties. Such variation may be caused by production-linked scanner-to-scanner variability or deterioration of properties over time (aging of lamps, scratches etc.) or differences between scanner types or brands. Here, scanners were maintained in a 30°, high humidity environment in a thermostat controlled room and kept covered by boxes, preventing inflow of light. See File S1, sections 1 and 8, for a complete description of the physical arrangement.

### Scan-o-matic software, image acquisition, and analysis

Scan-o-matic is written in Python 2.7 and can be installed from https://github.com/local-minimum/scanomatic/wiki. Matplotlib is used for graph production. Numpy, Scipy, and Scikits-Image are used for computation and analysis (van der Walt *et al.* 2011, 2014). Experiments are initiated from a web-interface, 7 min being the minimum time interval between scans (20 min as default) and 96, 384, or 1536 pinned plates being the allowed formats. Each series of scans is analyzed in a two-pass process. The first-pass analysis of images is performed during image acquisition, as outlined in Figure S1C. Using a fixture calibration model and orientation markers, the plate and transmissive gray-scale calibration strip positions are identified (Figure S2). The transmissive gray-scale calibration strip area is trimmed (Figure S3A, B), and segment pixel intensities are compared to manufacturer’s supplied opacity values such that a polynomial relating the two sets of values is established (Figure S3C). This polynomial is called upon to transform all pixels in the image to standardized gray-scale values. In the second-pass analysis (which is performed after the experiment is completed; schematically outlined in Figure S1D), a virtual grid is first established across each plate, with grid intersections matching the centers of likely colonies (Figure S4). At grid intersections, the local area is segmented, colonies defined relative to the local background, and pixel intensities of both are determined, compared, and converted into actual cell number estimates based on a cell calibration function. These cell number estimates reflect the 3D colony population size (Figure S5 and Figure S6). For the purpose of comparison, we also extracted the horizontal 2D area covered by each colony as the number of pixels included in each colony definition. See File S1, sections 2 and 3, for complete descriptions.

### Extracting, evaluating, and normalizing population growth rates from smoothed growth curves

Raw growth curves are smoothed to reduce the influence of noise, first using a median filter that removes local spikes and then using a Gaussian filter that reduces remaining noise (Figure S7 and supplemental movies File S2, File S3, and File S4). The minimum population doubling time is then extracted from smoothed growth curves based on a local regression over five time points in the region around the steepest slope (Figure S8). The time of extraction of the minimum population size doubling time, the error of the linear regression that underlies extraction of the minimum doubling time, and the growth curve fit to an initial value extended version of the classical Chapman-Richard model are also extracted from smoothed growth curves as auxiliary growth curve parameters. These auxiliary parameters serve as quality indices upon which the growth curves are quality ranked, offering the operator easy manual evaluation of growth curves for potential rejection due to too low-quality (∼0.3% were here rejected). Here, we also extracted the initial colony population size to understand the origins of spatial bias (Figure 3 and Figure S10); however, the initial population size is not a part of the Scan-o-matic analysis pipeline.

Every fourth experimental position was reserved for internal controls, creating a reference grid of isogenic wild-type (WT) populations. Colony population doubling times of these controls were used to establish a reference surface. First, controls with extreme values were removed. Remaining control positions were then used to interpolate a normalization surface. For each experimental colony, the log_2_ difference between its observed doubling time and the doubling time of the corresponding position in the normalization surface was calculated. When multiple plates were included in an experimental series (Figure 4), we first normalized for between-plates bias by shifting the mean of all colonies on a plate by a factor that transforms the mean of the nonspatially normalized controls to the mean of the controls over all plates. Because of the reference grid spatial normalization, this has a very small effect on data, with the effect representing bias that affects experiments but not controls. To call gene-by-salt interactions to exclude just general growth defects (Figure 4) we finally normalized growth rates in NaCl by subtracting growth rates in a basal (no added NaCl) environment. See File S1, sections 4 to 7, for complete descriptions.

### Wet-lab experimental procedure

**Figure 2:**
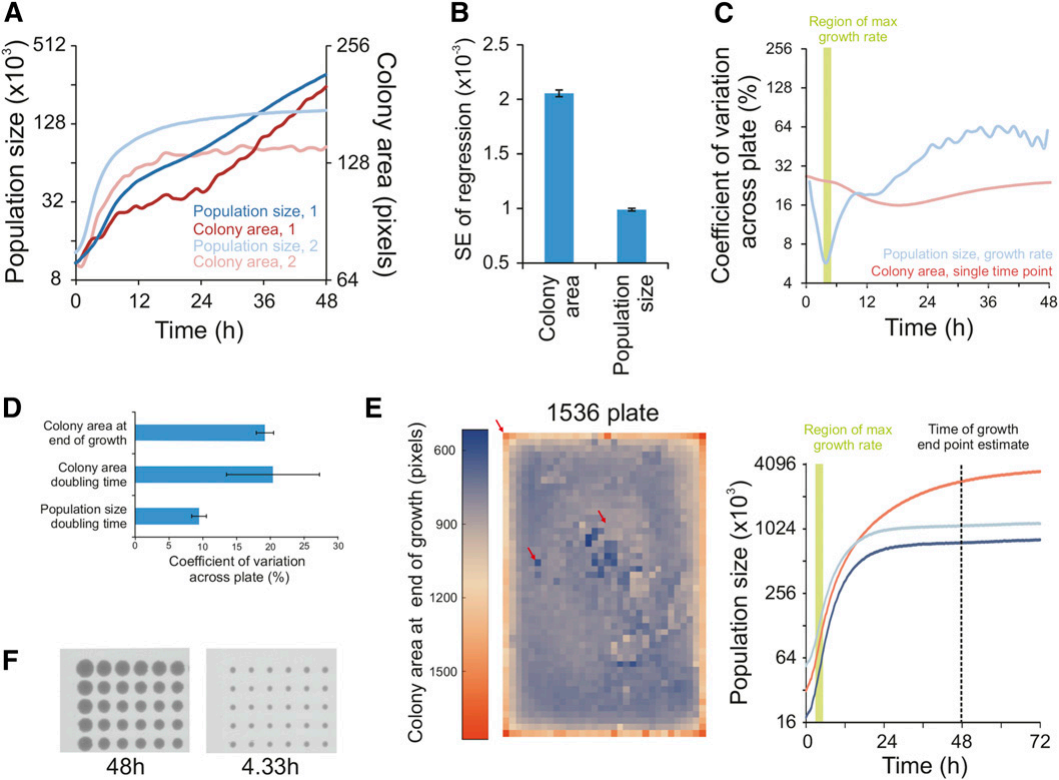
Dramatically enhanced measurement precision in microbial phenomics. (A and B) Comparing random noise in growth curves based on either colony area or colony population size. (A) Growth curves of two colonies, labeled 1 and 2, based on either colony area or population size (cell counts). *y*-axes are on log_2_ scale. (B) Estimating growth curve noise in the critical section of the curve when growth is maximal. Noise was measured as the standard error of the regression corresponding to the highest slope. Mean of 1536 genetically identical WT growth curves in an unstressed environment is shown. Error bars = SEM. (C and D) The sum of random noise and systematic bias over a plate, measured as the coefficient of variation across 1536 genetically identical WT colonies. (C) Precision as a function of time, for colony area size and colony population size growth rate. A single plate of unstressed populations is depicted. *y*-axis is on log_2_ scale. Green area shows the time period in which 96% (within 2σ of the mean time point: 4.15 hr) of experiments have their maximum growth rate. (D) Precision for single measure of colony area at end of growth (48 hr), colony area-based growth rate, and colony population size-based growth rate. The mean of four plates with different conditions (2% glucose and 2% galactose, with and without 0.85 M NaCl) is shown. Error bars = SEM. (E) Left panel: visual representation of the edge effect over a plate for colony area at the end of growth (48 hr). Each square corresponds to one of 1536 genetically identical WT colonies grown in 2% glucose, basal medium. Color intensity shows colony area, dark blue = 500 and red = 1800 pixels. Red arrows indicate the three colonies highlighted in the right panel. Right panel: population size growth curves for colonies indicated by red arrows in the left panel. Curve color matches the color of the squares in the left panel. Broken line: 48 hr time point that is typically used for endpoint growth measures. Green area: time region in which 96% (within 2σ of the mean time point, 4.15 hr) of the curves have their maximum growth rate. (F) Raw image of a corner section of a 1536 plate with genetically identical (WT) colonies growing in 2% glucose, basal medium, at 48 hr and at the time of maximal growth rate (4.33 hr). NaCl, sodium chloride; WT, wild type.

**Figure 3:**
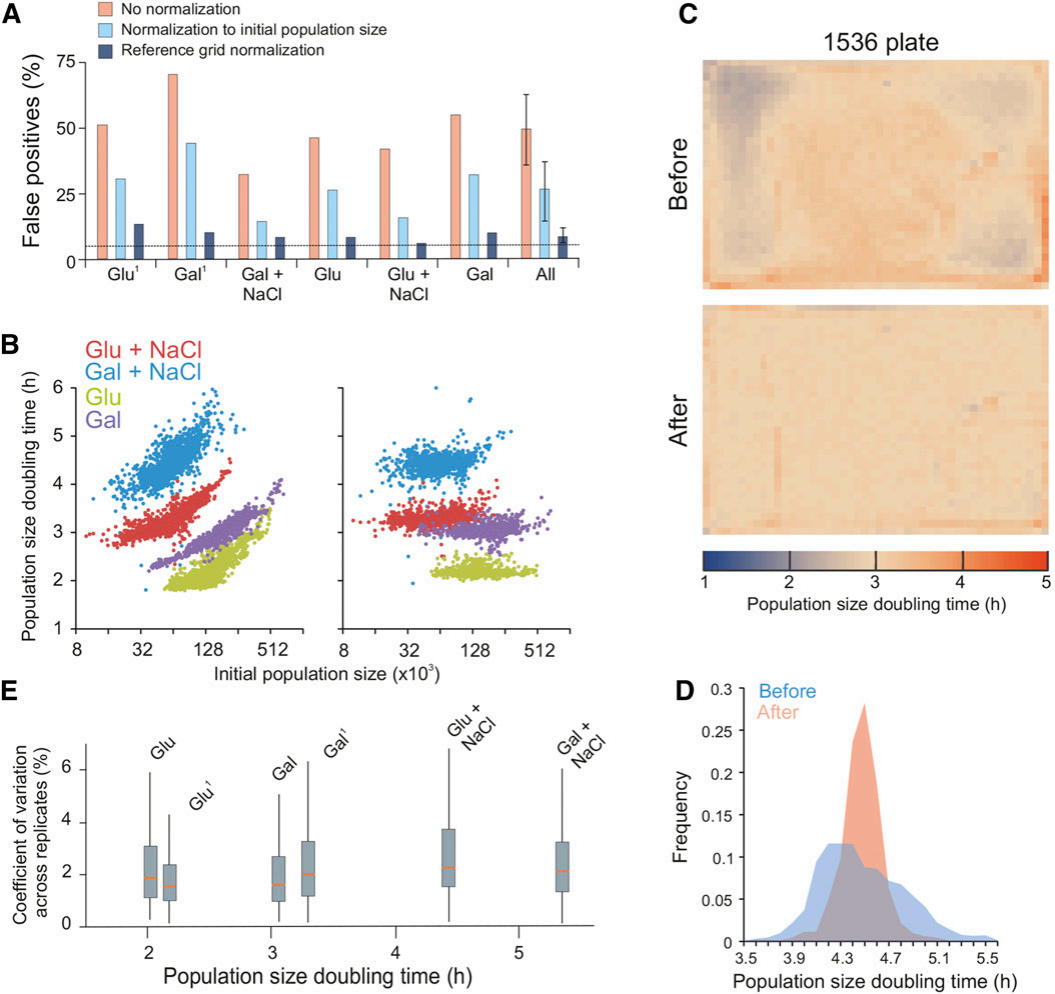
Comprehensive removal of spatial bias in microbial phenomics. (A) Fraction of false positives due to spatial bias within plates with genetically identical colonies (WT). Each plate corresponds to one distinct environmental challenge: 2% glucose or galactose, with or without 0.85 M NaCl. Superscript 1 = glucose or galactose plates stored (cold, dark, and enclosed in plastic) for 1 mo before use. On each plate, population size doubling times of immediately adjacent colonies (excluding every fourth position that was used as a control position) were statistically compared to those of nonadjacent colonies using a one-sample Student’s *t*-test (*H_0_* = 0 difference, α = 0.05). Assuming all variation to be random, *i.e.*, no spatial bias, the random expectation is 5% false positives (broken line) at this significance cut-off. Any systematic excess of false positives corresponds to spatial bias. Pink bars = before normalization, light blue bars = after normalization to initial population size, dark blue bars = after reference grid normalization. “All” indicates the mean of false positives over all six plates with error bars = SEM. (B) Population size doubling time as a function of initial population size. Left panel = before normalization, right panel = after reference grid normalization. All individual estimates over four of the six genetically homogeneous 1536 plates with different environmental challenges (2% glucose and 2% galactose, with and without 0.85 M NaCl) are shown. (C) Spatial bias is removed by reference grid normalization. Genetically identical reference colonies are pinned into every fourth colony position (lower right position in every tetrad of positions), creating a matrix of 384 control colonies on which a normalization surface of population doubling times is based. The local normalization surface is subtracted from each observation. Upper panel: distribution of population size doubling times of 1536 genetically identical colonies across a plate, before normalization. Each square corresponds to a colony position. Color indicates population size doubling time. Lower panel: as upper panel, but color represents population size doubling times after reference grid normalization. See also Figure S10. (D) Frequency distribution of population size doubling times, before and after reference grid normalization, in a sample plate (blue experiments in B, 2% galactose + 0.85 M NaCl. (E) Box plot showing variation in population doubling time estimates (*y*-axis, CV between adjacent colonies) after normalization within each of the six genetically identical (as in A) but environmentally distinct experiments, as a function of plate mean population doubling times (*x*-axis). Red line = median CV for all groups of adjacent colonies on plate, box = inter quartile range (mid 50%) of CVs, whiskers = complete range of CVs. CV, coefficient of variation; Gal, galactose; Glu, glucose; NaCl, sodium chloride; WT, wild type.

**Figure 4:**
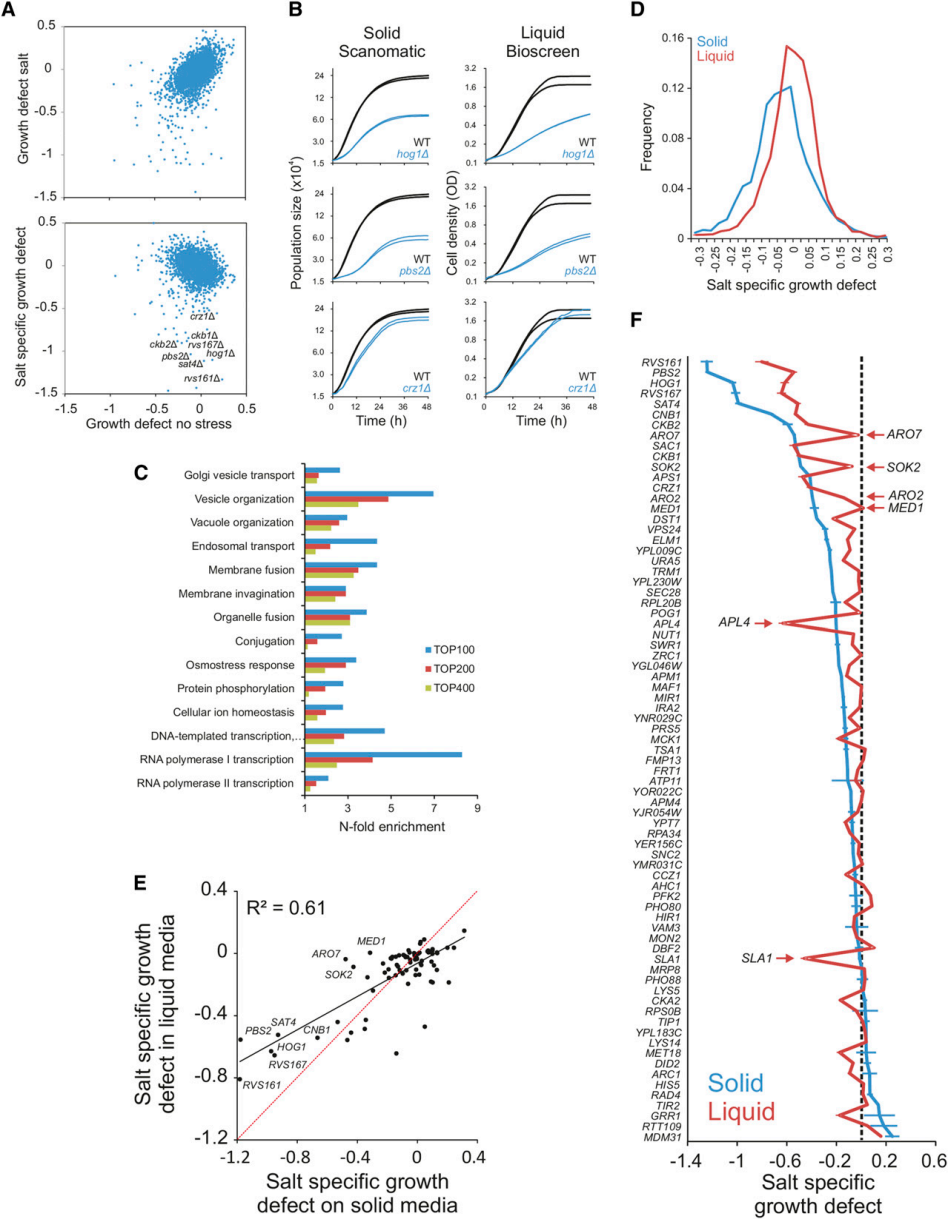
Recapitulating and extending the known salt biology with Scan-o-matic. The haploid *MATα* yeast deletion collection (BY4742) was cultivated in 2% glucose, with and without 0.85 M NaCl. Log_2_ population doubling times relative the control surface of WT controls were extracted. Negative values represent growth defects. (A) Upper panel: comparison of relative population size doubling times of the yeast deletion collection in presence and absence of NaCl. Lower panel: colony population doubling times in NaCl were normalized to corresponding measures in absence of NaCl, estimating NaCl-specific growth effects. These were plotted as a function of relative population doubling times in the absence of NaCl. Little correlation remains. (B) Growth dynamics of three sample deletion strains (*n* = 2) with NaCl-specific growth defects, in Scan-o-matic and during liquid microcultivation. (C) Functions enriched (Fisher’s exact test, false discovery rate, *q* < 0.05) among the top 100, 200, and 400 most salt sensitive deletion strains in Scan-o-matic. Cut-offs approximately correspond to relative growth defects larger than −0.27, −020, and −0.15. (D) Frequency distributions of salt-specific deletion strain growth effects, obtained by solid substrate cultivation in Scan-o-matic and by liquid microcultivation in a Bioscreen C (Warringer and Blomberg 2003). (E and F) A subset of 70 deletion strains, half of which were chosen to represent the most salt sensitive deletion strains detected in the global screen and half of which were chosen randomly, were recultivated in the absence and presence of 0.85 M NaCl at high replication, using Scan-o-matic (*n* = 24) and liquid (*n* = 6) microcultivation, respectively. Recultivations were performed in parallel (the same precultures) and in identical conditions, removing all conceivable systematic variation beside cultivation method. (E) Salt-specific growth defects in Scan-o-matic and liquid microcultivation regimes. Regression (black, Pearson *R*^2^ is indicated) and 1:1 lines (red) are shown. (F) Comparison of growth defects in mutants between agar and liquid growth. Deletion strains were ranked based on salt-specific growth effects during solid substrate cultivation and salt-specific growth effects were plotted for both conditions (solid and liquid). Error bars = SEM. NaCl, sodium chloride; WT, wild type.

Solid media plates were cast with 50 ml of Synthetic Complete (SC) medium: 0.14% Yeast Nitrogen Base (YNB, CYN2210, ForMedium), 0.50% ammonium sulfate, 0.077% Complete Supplement Mixture (CSM, DCS0019, ForMedium), 2.0% (w/v) glucose and pH buffered to 5.8 with 1.0% (w/v) succinic acid, and 0.6% (w/v) NaOH. Media was supplemented with 20 g/L of agar. Where indicated, 2.0% glucose was replaced by 2.0% galactose and/or supplemented with salt to 0.85 M NaCl. Three strain layouts were used: 1) all colonies being diploid BY4743 reference strain (Brachmann *et al.* 1998) (Figure 1, Figure 2, and Figure 3); 2) colonies corresponding to single yeast gene knockouts of the haploid BY4742 deletion collection (Giaever *et al.* 2002), with WT control colonies interleaved in every fourth position and *n* = 3 replicates of each strain in juxtaposition (Figure 4, A–D); 3) for the confirmation experiment (Figure 4, E and F) the same procedure was employed, but at high number of replicates (*n* = 24). The reference liquid media experiments (*n* = *6*) (Figure 4, E and F) were performed as previously described (Warringer *et al.* 2011). See File S1, section 8, for a complete description of the wet-lab procedures.

### Data availability

The authors state that all data necessary for confirming the conclusions presented in the article are represented fully within the article.

## Results

### A novel framework for high-resolution microbial phenomics on solid media

To develop a microbial growth phenomics framework that is both high-resolution, high throughput, and accessible to the average microbiology lab, we established an analysis pipeline based on transmissive scans of colonies expanding on the surface of a solid agar medium. This is achieved using high-quality, mass-produced, desktop scanner technology (Figure S1A; see File S1 for more detailed description of the procedure). Scans are initiated at preprogrammed intervals by means of a power manager that switches off scanner lamps immediately after scanning (Figure S1B). This precaution reduces confounding spatial bias from exposure of colonies closer to the lamp parking position to excessive light and temperature. Four solid media microcultivation plates, each containing from 96 to 1536 colonies, fit in each scanner. Given initial instructions, Scan-o-matic autonomously completes the entire pipeline from data acquisition to growth feature extraction.

The computational pipeline is divided into two separate processes, first and second pass analysis (Figure S1, C and D, respectively). The computationally less intense first pass analysis is run during image acquisition, and registers images as well as calibrates pixel intensities to the opacity values of a fixed standard. Plates are fixated within an acrylic glass fixture where their precise position is automatically detected during the first pass analysis using orientation markers (Figure S2, A and B). Each fixture also contains a transmissive gray-scale calibration strip. During the 1^st^ pass analysis, the observed pixel intensities for the segments of this calibration strip are related to fixed standard values given for each segment (Figure S3, A–D). The established gray-scale calibration function is then applied to all pixels on the whole image. This procedure effectively accounts for variations in pixel intensities in a scanner over time, differences in properties between scanners, and also compensates for much of the random noise that arises from digitalization effects (via interpolation: see File S1, section 3.4.1.3). The calibration converts pixel intensities into what is here termed pixel opacity values (Figure 1B; upper panel).

The more computer intense secondpass analysis is performed after all images in an experimental series have been acquired. It is performed in reverse chronological order, starting with the last registered image. This allows the more challenging analysis of early images, where colony population sizes are small, to utilize information about colony position from easier to analyze later images, where population sizes are larger. First, Scan-o-matic segments images and robustly localizes the center point of colonies, placing a virtual grid across the plate where each intersection corresponds to the predicted center point of one colony (Figure S4). Second, once the predicted center points have been established, Scan-o-matic precisely identifies colonies and defines pixels in each local colony area as colony pixels, local background pixels, or trash that cannot confidently be assigned to either (Figure S5). The local background pixel opacity values are then subtracted from the pixel opacity values of each colony pixel, producing background-subtracted pixel opacity values. Background-subtracted pixel opacity values are finally converted via a cell calibration function into cell counts per pixel (Figure 1B; lower panel). The cell calibration function was established by comparing background-subtracted pixel opacity values of > 40 colonies, covering the complete range of population sizes, to both FACS and diluted OD established cell counts (Figure S6, A–D). Cell counts per pixel are summed over the colony to produce precise estimates of colony population size and finally colony population size growth curves. The key steps of the second pass analysis are shown for one example colony in Figure 1C.

Because of the frequent recording of images and the high accuracy of the data, obtained growth curves require only very light smoothing. This is achieved using median and mean filters that remove most of the remaining noise (Figure S7). Animations connecting colony images to growth curves are shown in supplemental movies, File S2, File S3, and File S4. Suspected low-quality curves are flagged for visual inspection by the operator using various quality indices, with the operator typically having to reject < 0.5% of curves. Finally, population doubling times are extracted by local regression over the steepest part of each growth curve. Typically, growth is not exponential over substantial time periods (Figure S8). The whole process is performed with no need for fitting population size data to models based on prior assumptions of what growth curves should look like, because of the high frequency and the high quality of data.

Cultivation over a range of environmental contexts showed the vast majority of growth curves to be sigmoid in shape, to be minimally affected by noise and artifacts, and to have distinct, environment-specific properties typical also in liquid cultivations (Figure 1D). Following a lag phase of variable length, population doubling times reached a peak after around 4.15 hr in basal medium that corresponded to a raw population size doubling time of 2.0 hr (Coefficient of Variation, CV = 4.9%, *n* = 1536). Thereafter, growth rates declined and cultures finally reached the stationary phase, which in carbon-limited populations growing in basal medium corresponded to 4.5–5.5 population doublings over the whole growth period. Curve shapes were found to be independent of pinning density (384 or 1536 colonies) (Figure S9A). Final cell number in each colony increased at lower pinning density. This was expected, given the lesser competition for carbon and energy when the number of colonies on a plate is reduced. However, initial population sizes are smaller in the denser formats, because the smaller pins of the 1536 format pin-heads deposit fewer cells. The net effect was a longer growth span for each colony in the 1536 format relative to the 384 format (mean of 5.1 *vs.* 4.9 doublings, *P* < 10^−8^), marginally faster growth, and more precise estimates of the minimum population doubling time (Figure S9B).

### Enhanced measurement precision and accuracy of colony population size and growth rate

Highly time-resolved growth data offers conceptual advantages by allowing unbiased comparisons of populations with very different growth dynamics, with no need of fitting data to a preconceived model (Figure 1A). To test whether Scan-o-matic also offers other advancements, we compared its precision and accuracy to that of the current standard in microbial high-throughput phenomics: measures of the horizontal, 2D area occupied by a colony at a single endpoint. We considered plates containing 1536 genetically identical cultures (WT) and found growth curves based on colony population sizes to be considerably less affected by random noise than their colony area counter parts (Figure 2A). We quantified this improvement in precision using the standard error of the regression at the time of maximal growth rate. Overall, we found the colony area-based growth curves to be about twice as noisy as population size-based growth curves (Figure 2B). The improvement in precision becomes even more dramatic when considering the relative noise, *i.e.*, the standard error as a fraction of the signal strength (CV), because the signal strength of 3D growth vastly exceeds that of 2D growth. The difference in 3D relative to 2D colony information content increases as growth proceeds to reach a maximum at entry into the stationary phase. At this point, the 2D colony area has on average only doubled, whereas the 3D population size growth has increased by a factor 4.5–5.5 (Figure 2A, compare left and right *y*-axes).

Diminished random noise provides an incomplete picture of technical achievement because it overlooks systematic bias. We therefore evaluated the total measurement error across plates, *i.e.*, including both random and systematic variation between the 1536 genetically identical colonies. Following the total measurement error over time for 2D colony areas and 3D population size growth rates, respectively, we found it to shift dramatically depending on growth state (Figure 2C). Precision was initially low for both 2D area and 3D population size. This followed from the relatively large variations in the robotic delivery of cells and from the larger influence of randomness in defining colony borders when pixels representing each colony are few (initially ∼100 pixels) and when true borders pass through pixels. The total error for 2D colony areas then slowly decreased, the CV reaching a minimum late in the growth phase. CV then increased again, as competition for resources intensified and only outer frame colonies with fewer neighbors continued to grow. Precision for 3D population size growth rates began similarly low, but increased dramatically, with CV reaching a minimum at around 4.5 hr. At this point, it greatly exceeded the precision of 2D colony area measurements. The time of maximum measurement precision coincided with the time period at which the population size growth rate was maximal. We therefore extracted maximal growth rates, *i.e.*, minimum population size doubling times, as not only the conceptually most relevant feature of growth curves, but also both the most stable and precisely estimated feature.

Overall, considering six diverse environments, 3D population size doubling times were estimated with about half the error of single endpoint measures of 2D colony area (Figure 2D). This gain was not only due to the more precise measurement of 3D population sizes, it also followed from the much lower competition for resources between colonies at the time at which growth is maximal and thus from lower spatial bias (Figure 2, E and F). This avoidance of colony competition effects via maximal growth rate estimates is also conceptually important, because growth rate estimates avert confounding comparisons of cell populations that are in different physiological states at the time at which growth is estimated (Figure 1A).

### Comprehensive removal of spatial bias by control-surface normalization

Careful attention to experimental standardization can reduce, but never completely remove, systematic bias between colonies, because much of that bias emerges as a consequence of design parameters that cannot be altered. The growth advantage of populations in close proximity to the plate edge is one of several such biasing effects that are intrinsic to the experimental design. Randomization of replicates across biasing factors can potentially account for this bias, but requires that biasing factors have been identified and is logistically extremely challenging when ≈ 100,000 experiments are run in parallel. The most realistic approach to account for bias is therefore by normalization, *i.e.*, to identify error patterns in the acquired data and treat them such that these error patterns are compensated for. In solid medium microbial phenomics screens, the most evident bias that remains after careful technical standardization is the bias between experimental positions within a plate. Assuming the common, but naïve, null hypothesis that all systematic bias has been removed by standardization, we would expect ≈ 5% false positives for each of the above single genotype (WT) 1536-format plates (α = 0.05, Student’s *t*-tests). However, even after very extensive standardization (see File S1, sections 1.1 and 8) and using the most accurate measure of growth, minimum population size doubling times, to minimize error, we scored many more (∼10 ×) false positives than chance expectation (Figure 3A). Thus, substantial systematic bias remained. The change in error-to-signal ratio over time (Figure 2C) indicated two distinct types of errors: an initial bias from systematic variations in the number of cells deposited at different positions on plates, and a later bias from increasing competition for resources between neighboring colonies (Figure 2E). Of these, the former is the most important at the time when the maximum growth rate is extracted, as seen from the strong correlation between the minimum population size doubling time and the initial population size (Figure 3B; left panel). However, after normalizing minimum population doubling times to the initial population size, we still found that about half of the excess of false positives remained (Figure 3A and Figure S10). Therefore, we rejected initial population size normalization as an unsatisfying approach. Further scrutinizing the topology and strength of spatial bias, we found both to vary dramatically between plates and environments, producing unpredictable and very complex patterns (Figure 3C and Figure S11; left panels). The major tendency was a positive correlation between neighbors. To meet this challenge of unpredictable and highly shifting bias, we developed a normalization approach that does not require any prior assumptions of what causes the spatial bias and is independent of its topology. We replaced every fourth position on the plates with isogenic controls, creating an array of 384 evenly distributed reference positions (Figure S12). Assuming that spatial bias is independent of genotype, this reference grid of control colonies should well capture the spatial bias affecting colonies in the remaining 1152 experimental positions. By interpolation from the population doubling times of control positions, after the removal of control outliers (Figure S13), we constructed a spatial bias control-surface that estimates the spatial bias affecting each individual position. Subtracting this spatial control-surface of population doubling times from the actual estimates, in each experimental colony position, we obtained normalized population doubling times of experiments’ relative controls. Performing this normalization for the plates containing 1536 genetically identical populations (see above), we found the visually detectable bias to be vastly reduced (Figure 3C and Figure S11; right panels) and the correlation of population doubling times to initial population size to be almost gone (Figure 3B; right panel). Critically, it also resulted in a false positive rate that approached random expectations and that was an order of magnitude lower than the one obtained without spatial normalization (Figure 3A). Spatial normalization resulted in population doubling times that were approximately normally distributed (Figure 3D) and removed the correlation between relative error (CV) and signal strength (Figure 3E). These are both fundamental requirements for the application of standard parametric statistics. After spatial normalization, the total measurement error in an unstressed environment was around 2% (CV) of the signal, approaching the 1.5% achieved with state-of-the-art microcultivation in liquid media (Warringer *et al.* 2003, 2011). Thus, although some remnants of spatial bias did remain, this control-surface normalization resulted in a measurement precision that almost matched the best that can be achieved with lower throughput approaches.

### Scan-o-matic extends and nuances the salt biology of baker’s yeast

Gene-by-salt interactions have previously been called by scoring NaCl-sensitive gene deletion strains with state-of-the-art microcultivation, and the underlying biology has been extensively studied (Warringer *et al.* 2003). To evaluate the capacity of Scan-o-matic to recapitulate established knowledge, we compared our new method with previous microcultivation experiments calling salt-sensitive mutants in the complete yeast gene deletion collection.

By comparing growth in the presence and absence of added NaCl (Figure 4A), we identified a large number of salt-sensitive gene deletions. Many of these deleted genes, such as those encoding Hog1 and Pbs1, which are part of a signaling cascade that activates the osmo-response, or the salinity-responsive transcription factor Crz1, corresponded to proteins that are well known to control salt tolerance (Hohmann 2002). Growth curves on solid and liquid media in these deletion mutants were similar overall (Figure 4, A and B). Among the salt-sensitive mutants identified in this genome-wide screen, 14 biological processes were enriched. Many of these, *e.g.*, ion homeostasis, ion transport, response to osmotic stress, and endocytosis and vacuolar transport (Figure 4C), have previously been shown to be of importance during salt exposure (Warringer *et al.* 2003). Thus, prominent parts of the salt protection system appeared to be shared between liquid and solid growth and to be well captured by Scan-o-matic.

Nevertheless, the distribution of gene-by-salt interactions was substantially wider using Scan-o-matic, with a distinct shoulder toward salt sensitivity (Figure 4D). Thus, at any given signal strength and at any given significance stringency, more and stronger interactions were identified on agar compared to liquid microcultivation. The amplification of signal strengths of growth phenotypes was also evident in the absence of NaCl. This suggests the increase in phenotypic variation on solid media to be a general phenomenon (Figure S14A). More disconcertingly, some genes that were required for salt tolerance during liquid microcultivation were irrelevant for salt tolerance when scored on agar, and vice versa. Overall, the correlation between the here reported results and earlier published data sets from other laboratories was modest (Figure S15). This implied that cultivation method could have a substantial impact on growth phenotypes in general, and salt sensitivity in particular. Unfortunately, our two screens were separated by almost a decade and many variables other than cultivation method, including strain marker background and nutrient medium composition, differed between them, precluding firm conclusions. Other published data sets provided no help in this regard, because of the vast number of additional parameters differing between these and the current screen (Figure S15).

To exclude all sources of variation other than cultivation method, we therefore rescreened a subset of salt sensitive and non-salt sensitive deletion strains with both cultivation methods. We kept all factors constant, except cultivation method, and employed a large number of replicates in randomized positions, thereby maximizing both precision and accuracy. We found correlation between cultivation methods to be only intermediate (*r*^2^ = 0.5–0.6), both in the presence and absence of salt (Figure 4E and Figure S14, B and C). As roughly 10% of the total variation could be explained by technical noise, the remaining 30–40% derived from phenotypic effects imposed by differences in cultivation method.

Both cultivation methods called the most important salt-defense regulators. This included the signaling components Hog1 and Pbs1, Rvs161 and Rvs167 (which reorganize the action cytoskeleton during Na^+^ stress) (Balguerie *et al.* 2002; Lombardi and Riezman 2001), the regulatory subunits Ckb1 and Ckb2 of the casein kinase that regulates Na^+^ extrusion (Glover 1998), the regulatory subunit of calcineurin Cnb1 plus its downstream transcription factor Crz1 that controls Na^+^ efflux at current pH (Stathopoulos and Cyert 1997), and the cation extrusion regulator Sat4 (Mulet *et al.* 1999)(Figure 4E). However, many mutants were salt sensitive in only a single cultivation regime. Thus, despite an abundance of amino acids in the media, the absence of either Aro2 or Aro7, required for synthesis of aromatic amino acids, was critical to salt tolerance only during colony agar-growth, as were the removal of the RNA pol II component Med1 or the inhibitor of pseudohyphae formation, Sok2 (Figure 4F). In contrast, the presence of the clathrin coated vesicle component Apl4 and the actin cytoskeleton associated Sla1 were important to salt tolerance only for cells reproducing dispersed in a solution but not on agar. Overall, 81% of retested gene deletions differed significantly (Student’s *t*-test, FDR *q* < 1%) in salt tolerance between solid and liquid media growth. The majority (68%) of the mutants displayed more severe salt sensitive phenotypes on solid media. Thus, whereas our new approach recapitulated the essence of established yeast salt biology, it also highlighted the large influence of cultivation approach on both the quantitative and qualitative aspects of phenotypes.

## Discussion

Scan-o-matic sets a new standard in microbial growth phenomics by simultaneously allowing high-resolution, high precision, accuracy, and throughput analysis. Even in our prototype arrangement, with five computers and 15 scanners, we are capable of running more than 92,000 individual growth experiments in parallel, with automated extraction of minimum population doubling times. This throughput is orders of magnitude better than what can be achieved by liquid microcultivation. In recent proof-of-principle studies, we have illustrated the importance of simultaneous high-resolution and high throughput analysis, using Scan-o-matic to completely decompose trait variation in diploids into its dominant, epistatic, and additive components (Hallin *et al.* 2016), and to predict traits of individuals with near perfect accuracy from their genome and the genome and phenome of relatives (Martens *et al.* 2016). Here, we showed that Scan-o-matic nicely captures the known salt biology of *S. cerevisiae*, largely uncovering the same cellular functions as earlier found to be important by microcultivation (Warringer *et al.* 2003). However, we also firmly established the context-dependence of the salinity response with several deletion mutants displaying rather distinct effects between agar- or liquid-growth cultivation. This provides stark arguments for taking the growth regime into account when comparing phenotypes obtained using different platforms and when annotating genes in phenotype databases. Surprisingly, more and stronger phenotypic interactions were identified on agar compared to liquid microcultivation. This raises the question of what is the most natural lifestyle for *S. cerevisiae*: being dispersed in liquid media or grown as microcolonies on solid substrates? *S. cerevisiae* in nature is typically found on the surface of tree bark, in the top layer of soil in forests, the intestinal content of insects, and on damaged or very ripe fruits (Liti 2015). Thus, homogeneous liquid cultivations where all individuals have equal access to nutrients, are equally exposed to detrimental factors, and are perfectly genetically mixed such that mothers and daughters are randomly dispersed in the population (as in liquid growth), may not be a good representation of how natural yeast populations grow and have been selected during evolution. Solid media cultivation might, thus, provide a more natural context for testing and revealing gene function in this species.

Despite the high performance of Scan-o-matic using our current protocols and experimental set-up, we have not exhausted all avenues for optimization. The validity of the data can be improved either by increasing precision, *i.e.*, reducing random noise, or by reducing bias. Reductions in random noise would be achievable by changing from 8- to 16-bit image depth. However, 16-bit image depth increases file size considerably, and given the vast amount of hard-disc space taken by the files currently generated from one single experiment (≈ 5–10 Gb) this would pose a serious challenge to the logistics of data storage and analysis. Analyses of 16-bit image depth data are also poorly supported in the Python packages upon which Scan-o-matic is based. This is not a trivial challenge to overcome. Second, precision could be improved by increasing image resolution. This would ensure that each colony is represented by a larger number of pixels, reducing both the stochastic noise originating in errors in assigning pixels to colonies and the noise deriving from true colony edges going through, rather than following the boundaries of, pixels. However, increasing image resolution necessarily reduces scanning speed and thereby increases exposure to radiation, heat, and dehydration from light. This is a serious concern because exposure to intense light severely impedes yeast growth (Logg *et al.* 2009). Here, the scanning speed-to-image resolution trade-off was set such that the growth of the extremely light-sensitive *hog1* and *pbs2* mutants were unaffected by the scanning, *i.e.*, we ensured that the confounding effect of light exposure would be effectively zero under basal growth conditions (data not shown). Third, precision could be improved by increasing scanning frequency (we currently use 20 min between scans, as default). This would result in growth curves represented by a larger number of measures, allowing more exact curve smoothing to filter out noise. Again, however, this increases light stress and associated impact on cells, and creates challenges in the logistics of handling and storing data, because of the increase in data amount.

Systematic bias can be accounted for by standardization to avoid error sources, by randomization of replicates over error sources or by normalization to remove the effects of error sources, *a posteriori*. Here, we have meticulously standardized experimental procedures as described in File S1 (sections 1.1 and 8) to minimize systematic variations in the robotic transfer of cells, precultivation regimes, cultivation parameters, and data acquisition. Such careful attention to standardization is essential; normalization is complementary to, not a replacement for, standardization. Nevertheless, standardization can never completely remove the spatial bias that causes systematic variations between experimental positions on a plate, because a plate due to its edges constitutes a heterogeneous environment. We found about half of the excess of false positives that persisted after standardization to derive from spatial differences in initial population size (Figure 3A). Differences emerge from variation both in the number of cells collected from each preculture colony, and the fraction of collected cells that are deposited on the cultivation plates. The size of the preculture colonies plays a role in the number of cells deposited, with fewer or no cells collected from very small precultures. It is also possible that the length of plastic pins varies slightly across a pin pad. In addition, the variations in deposition emerge due to slight differences in medium thickness across the plate which, in turn, depend on where, how, and when the medium is poured into plates and solidifies, how much the agar creeps up along the plastic walls, and the patterns of water loss across the plate. The correlation between initial population size and population doubling time is negative, *i.e.*, the higher the initial population size, the slower the estimated growth. We believe this to be caused by the absence of an extensive exponential phase: at higher initial population sizes resource limitation sets in before the theoretical maximum growth rate has been reached. Decreasing the mean initial population size by shifting to thinner pins, *e.g.*, to a 6144 pinning format, would potentially further reduce the spatial bias derived from variation in population size. Unfortunately, the current image resolution is not sufficient for the accurate detection and definition of 6144 format colonies. An increase in image resolution would, in that case, be required, which would in turn increase the image acquisition time and bias associated with radiation and light exposure. A reduced initial population size also translates into an increased risk of sampling errors, such that nonrepresentative cells are transferred from precultures to found experimental populations.

The spatial bias deriving from sources other than variations in initial population size has diverse origins. At the point of maximum growth rate, competition for resources, and the associated influence of the local density of cells, is small. Nevertheless, when the diffusion of nutrients through the solid matrix is slow relative the growth rate of populations, *e.g.*, in basal environments, there might be some competition. In addition, the competition for resources is influenced both by the number and the size of colonies in each region, and the variations in resource abundance that derive from any variations in the thickness of the medium. Designing experiments such that genotypes with similar growth rates are cultivated on the same plate may reduce the spatial bias from competition effects, but at the cost of running the risk of systematic differences between plates. Conceivably, the concentration of the growth limiting resource could be fine-tuned such that competition for this resource has less effect on the maximum growth rate, but given the unavoidable differences in distance to the edge, it seems implausible that the effect could be removed altogether. The differences in the local density of cells across a plate not only manifests as competition for resources, but also as a faster build-up of compounds produced and secreted as a function of growth in regions where the local density of cells is high. Some of these compounds can have adverse impacts on growth. Notably, this is true for many of the acidic metabolites that are generated by carbon catabolism and that reduce the external pH, creating spatial variations in pH across plates that depend on both distance to edge, genotype, and medium thickness (Figure S16). Note that the spatial variation in pH is exacerbated by the common use of ammonium as a nitrogen source: ammonium is basic, meaning that the initial pH is high, and the pH drops rather drastically when ammonium is consumed as a function of growth (Hensing *et al.* 1995). The acidification in unbuffered media can reach down to pH 2.5. At this point, growth is severely affected, with the degree of effect depending on both genotype and environment (Walker *et al.* 2014). Therefore, spatial variations in pH may not just be consequences of spatial bias in growth, but drive such bias. This underscores the importance of buffering the medium. Some spatial bias is also likely to derive from variations in the physiological state of the founding cells. Colonies are structured genetically, with mothers and daughters staying in close proximity, but also physiologically, with cells toward the periphery of the colony horizontal area having access to more nutrients and for a longer time period than their starved relatives in the colony center. When cells are collected from precultures, systematic spatial differences in whether edge or central cells of a colony are collected do emerge. These depend on the precision of the *x-y* positioning of the robotic pin-head holder, size differences of colonies, and medium surface unevenness. This means that some experimental colonies are founded by predominantly starved, or even autophagic, cells. Others are founded by cells with nutrient storages intact. This, as well, is difficult to address purely by standardization.

These and other sources of spatial bias combine to create the highly variable and unpredictable patterns that are seen in Figure S11. Avoiding these patterns completely, by standardization, is not feasible because many of the error sources, including edge effects, are intrinsic features of the experimental design. Perfect randomization of replicates across experimental positions would reduce the false positives that follow as a consequence of the spatial bias. However, randomization does not remove error. Rather, it transforms systematic errors into random errors. The number of false positives therefore decreases but false negatives increases. Perfect randomization is also effectively impossible to achieve when 100,000 experiments are run in parallel. We developed an unbiased normalization approach that sacrifices 25% of the experimental positions, dedicating these positions to internal controls whose growth rates serves as the basis for estimating and adjusting for the spatial bias *a posteriori*. The basic requirement for the normalization surface to adjust for bias is that the same spatial bias is attached to both control and experimental positions. Therefore, it is essential that controls and experiments are treated identically: they must have as similar genetic and environmental histories at the initiation of the experiments as possible. Most importantly, controls must be interleaved with experiments on the same plate at the preculture stage, at the latest. Otherwise, both initial population size-linked and other types of bias will differ between experiments and controls as evident from the discussion above. It should be noted that, because of how the 1536 colony format is constructed (Figure S12), no control colonies can be placed in two of the four outer edges of the colony arrays. This makes the spatial bias correction slightly weaker in the vicinity of these two edges, in particular in fast growth environments. The associated error remaining after normalization will therefore tend to be slightly higher. Alternative, more complex pinning schemes could potentially address this issue, but at the cost of very challenging logistics.

Earlier published posterior correction procedures have used spatial variations in the performance of experimental colonies themselves (Baryshnikova *et al.* 2010). However, the use of experimental data, both as correction input and as final output, risks creating unsound statistical dependencies and associated artifacts. In general, it is not recommended. These approaches also assumed spatial bias to manifest primarily as an edge effect and that this bias can be compensated for in a row/column-wise manner. This is a reasonably valid assumption for data obtained from colonies that have ceased to expand because of nutrient depletion. However, this edge effect is not the major driver of bias at the time of maximal growth rates. Consequently, the bias remaining after control-surface normalization does not have a pronounced edge character to it. Adding a posterior correction layer of row/column based normalization would risk introducing, rather than removing, artifacts. It is also debatable how local the correction for spatial bias should be, *i.e.*, what weight should be given to controls at different distances from the specific colony position to be normalized. In our basic settings, we have elected to be conservative; *i.e.*, we assume a softly undulating rather than a highly rugged spatial bias landscape. Mostly, this agrees with the observed spatial bias topology, as can be seen in Figure S11. However, when fault lines are sharp, such that the bias changes dramatically between neighboring positions, we typically fail to fully compensate for this bias. It is challenging to envision a normalization approach that is capable of accommodating these sharp fault lines without also overcompensating in regions where bias is less local.

Beyond addressing noise and bias, there are also opportunities for conceptual advances. Condensation of population growth curves into estimates of maximal growth rates, while taking advantage of the superior precision at these time points, makes poor use of the vast trove of accumulated data. A standard 72 hr experiment encompasses 217 data points, only five of which are currently used for the extraction of growth data that are retained in downstream analysis. Time before net growth commences (growth lag) and the total gain in population size (growth efficiency) are additional fitness components that have traditionally been extracted from growth curves (Cooper 1991). Given that improvements of these phases are capable of driving adaptation independently (Ibstedt *et al.* 2015), they should certainly be the target of efforts to improve the analysis pipeline. However, much higher noise in the beginning and end of the net growth phase means that more attention to the experimental design and analysis procedure is needed before sufficient robustness can be achieved for these fitness components. Beyond lag and efficiency, colonies rarely maintain maximal growth rates over extended periods of time. In addition, growth often becomes multiphasic (Warringer *et al.* 2008), and this constitutes a serious challenge both for the standardized extraction of growth variables and normalization to account for bias. Finally, it is clear that the microbial phenomics field would benefit substantially from standardization. As seen in Figure S15, the overlap between studies that are supposedly rather similar is at the most moderate, presumably due to the use of a variety of nutrient media, cultivation methods, and genetic backgrounds. This low reproducibility of experimental results does not necessarily indicate that results obtained in any individual study are spurious, but rather suggests that they are only valid in a very limited genetic or environmental context, and should therefore not serve as the basis for generalized conclusions. Standardization will certainly improve reproducibility between laboratories but risks restricting microbial phenomics discoveries to the genetic and environmental context chosen for generalization. Future expansions aiming to exploit the entire growth curve and to further optimize precision and accuracy will ensure that the introduced Scan-o-matic framework provides truly high-quality microbial phenomics data for extensive cohorts of individuals to generate well-populated, highly resolved, and standardized phenotype databases.

## Supplementary Material

Supplemental Material
