# Supplementary material for: Scan-o-matic: High-Resolution Microbial Phenomics at a Massive Scale
Source: G3 (Bethesda). 2016 Jun 30;6(9):3003–14. doi: 10.1534/g3.116.032342 (PMC5015956; doi:10.1534/g3.116.032342)
Supplement: Supplemental Material [file supp_6_9_3003__index.html]

Scan-o-matic: High-Resolution Microbial Phenomics at a Massive Scale — Supplemental Material 

# Scan-o-matic: High-Resolution Microbial Phenomics at a Massive Scale

## Supplemental Material for Zackrisson *et al.*, 2016

**Files in this Data Supplement:**

- Figure S1 - Scan-o-matic process overviews. (.pdf, 2 MB)
- Figure S10 - Normalization by initial population size. (.pdf, 3 MB)
- Figure S11 - Spatial bias before and after reference grid normalization. (.pdf, 1 MB)
- Figure S12 - Construction of a reference grid of controls using a custom designed Scan-o-matic pinning program. (.pdf, 952 KB)
- Figure S13 - Reference grid normalization with and without removal of deviating controls. (.pdf, 1 MB)
- Figure S14 - Scan-o-matic performance of gene deletion strains in absence of stress. (.pdf, 122 KB)
- Figure S15 - Comparing Scan-o-matic and previously published yeast growth data. (.pdf, 440 KB)
- Figure S16 - Spatial bias in the form of local changes in pH across solid media plates. (.pdf, 2 MB)
- File S1 - Supplemental materials and methods. (.pdf, 778 KB)
- Figure S2 - Localizing and positioning image orientation markers relative the fixture calibration model. (.pdf, 471 KB)
- Figure S3 - Calibrating pixel intensities using a calibration strip. (.pdf, 2 MB)
- Figure S4 - Placing a virtual positioning grid across each plate image. (.pdf, 3 MB)
- Figure S5 - Assigning pixels to colonies and local background and estimating background subtracted pixel opacity values. (.pdf, 555 KB)
- Figure S6 - Converting background subtracted pixel opacity values to cell counts. (.pdf, 1 MB)
- Figure S7 - Reducing noise in growth curves. (.pdf, 266 KB)
- Figure S8 - The period of maximal growth rate is short and growth is sometimes complex in expanding yeast colonies. (.pdf, 273 KB)
- Figure S9 - Effect of pinning format on Scan-o-matic growth curves. (.pdf, 203 KB)
- File S2 - Animations connecting raw colony images to growth. (.avi, 788 KB)
- File S3 - Animations connecting raw colony images to growth. (.avi, 1 MB)
- File S4 - Animation illustrating spatial bias in population size as a function of time. (.avi, 2 MB)
