# Supplementary figures and images for: Scan-o-matic: High-Resolution Microbial Phenomics at a Massive Scale
Source: G3 (Bethesda). 2016 Jun 30;6(9):3003–14. doi: 10.1534/g3.116.032342 (PMC5015956; doi:10.1534/g3.116.032342)

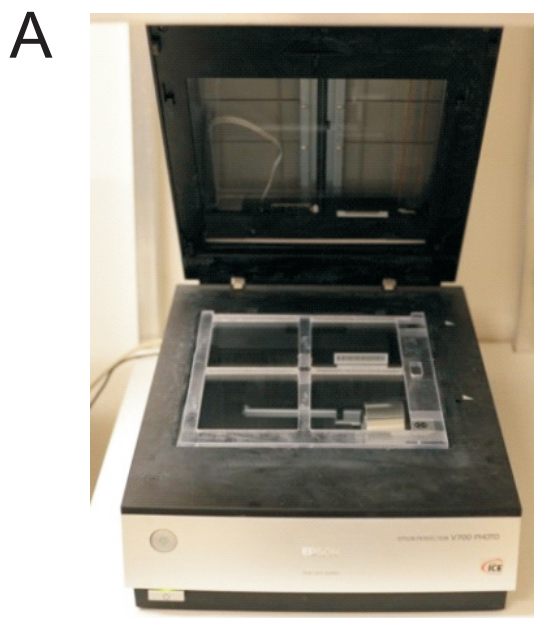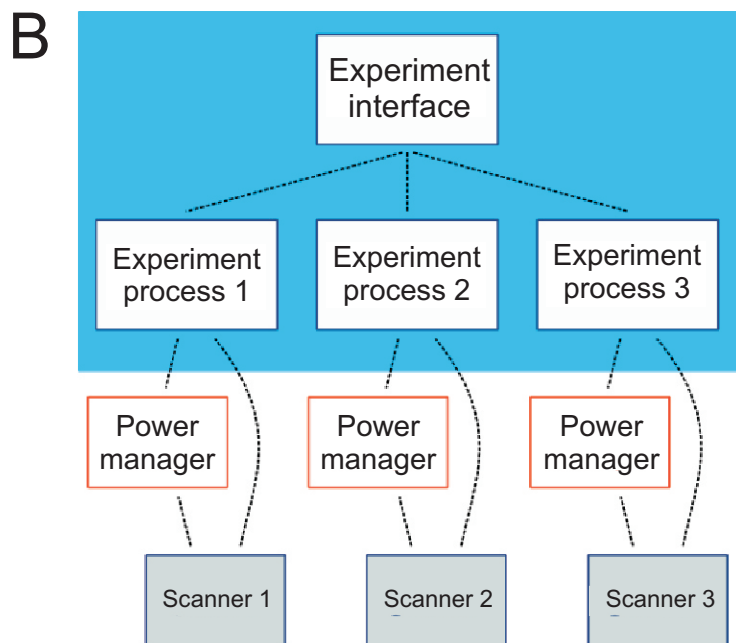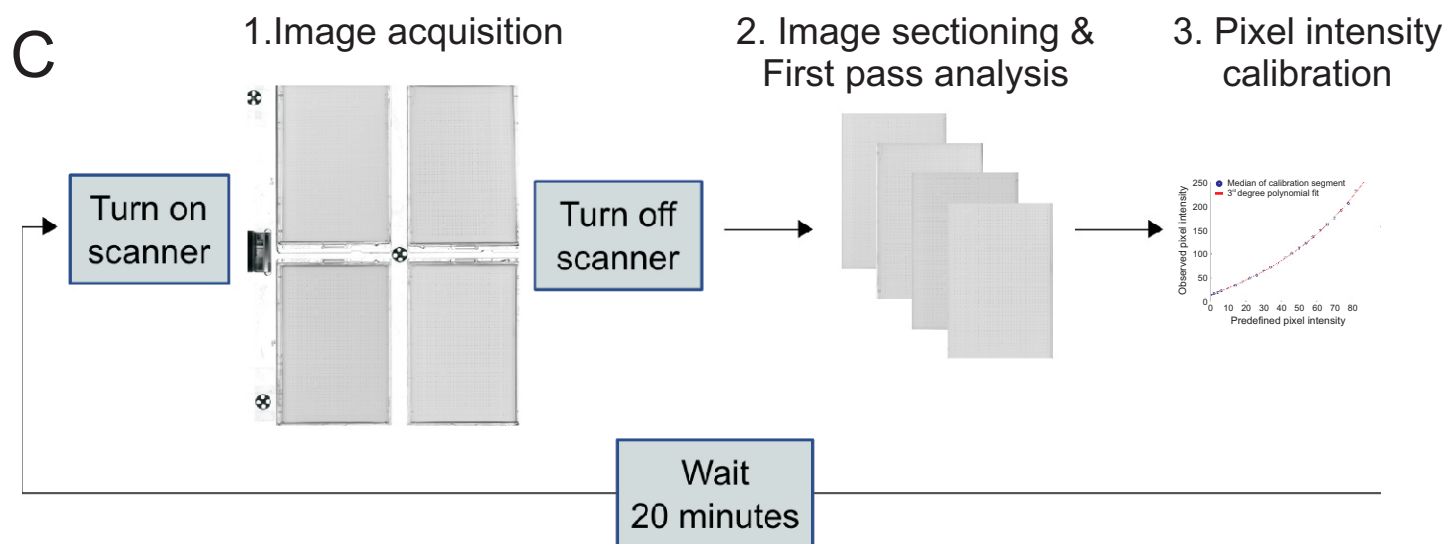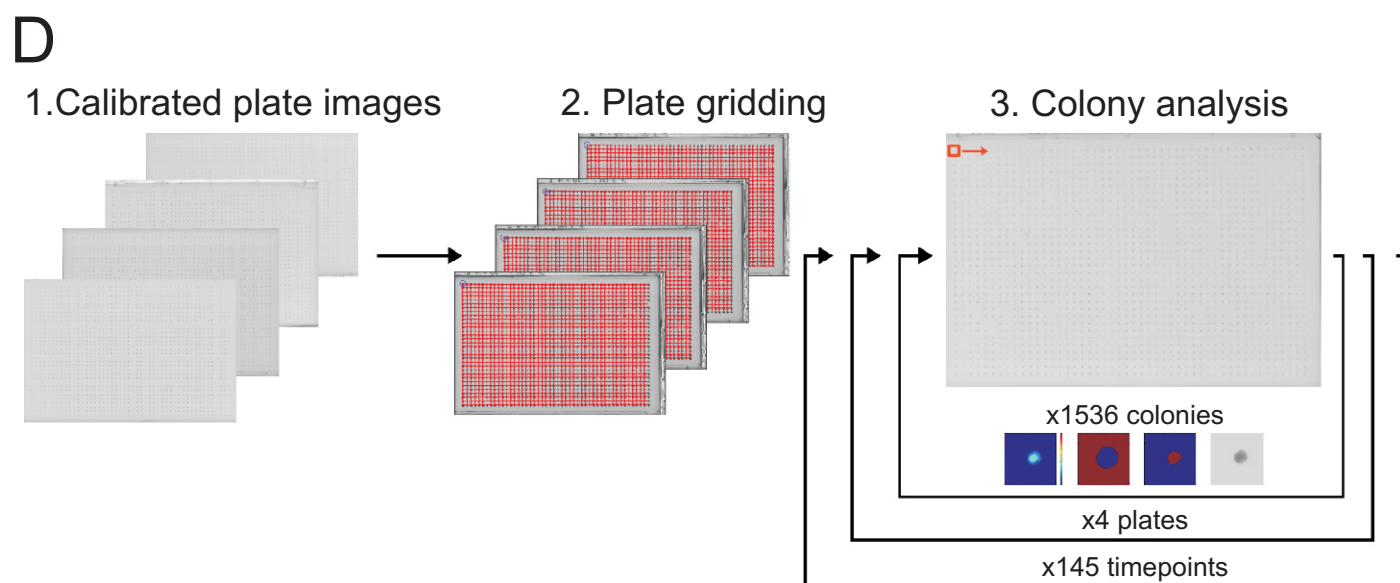

Figure S1 Zackrisson *et al*

Supplement: Supplemental Material [file supp_g3.116.032342_FigureS1.pdf]

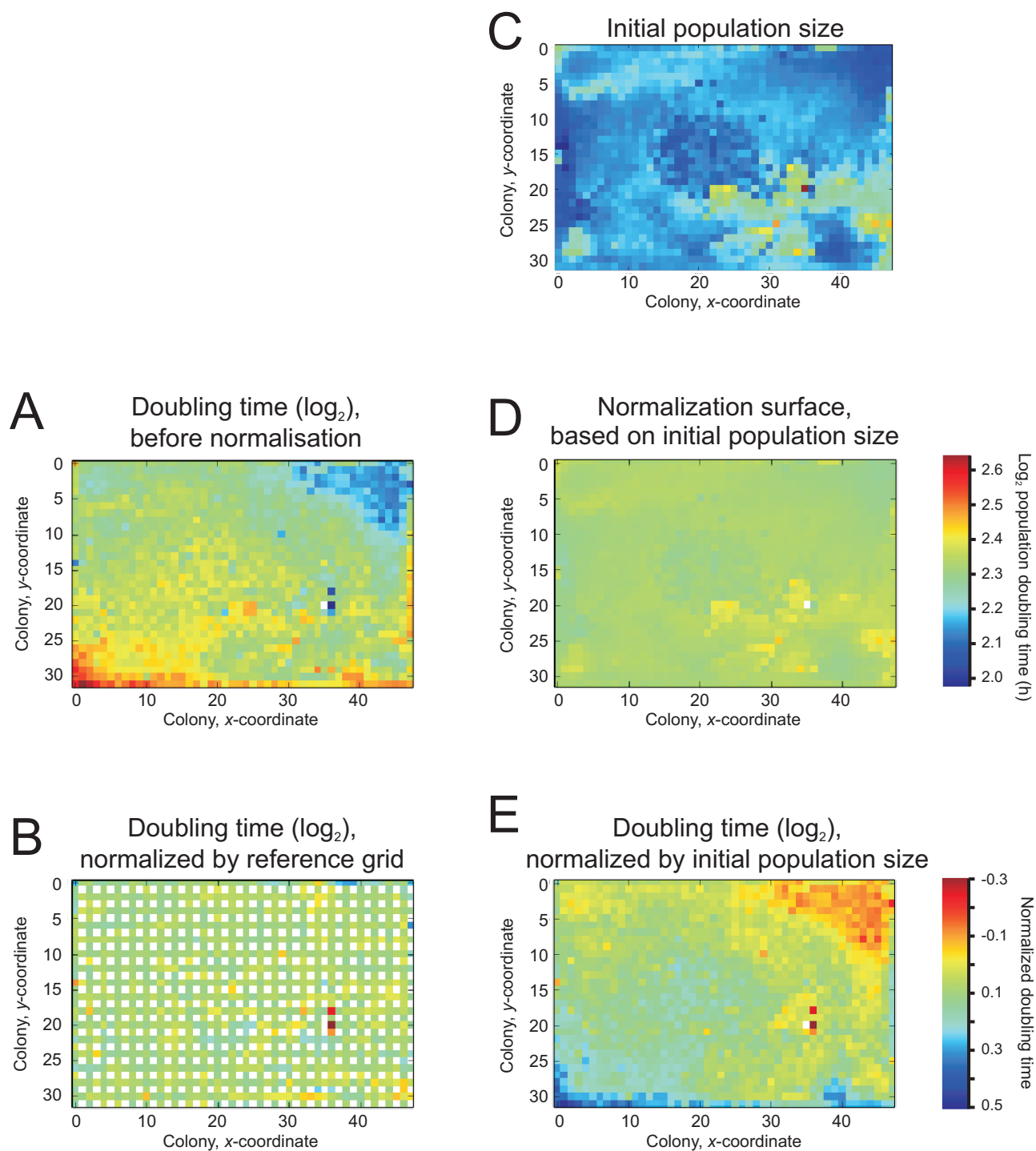

Figure S10 Zackrisson *et al*

Supplement: Supplemental Material [file supp_g3.116.032342_FigureS10.pdf]

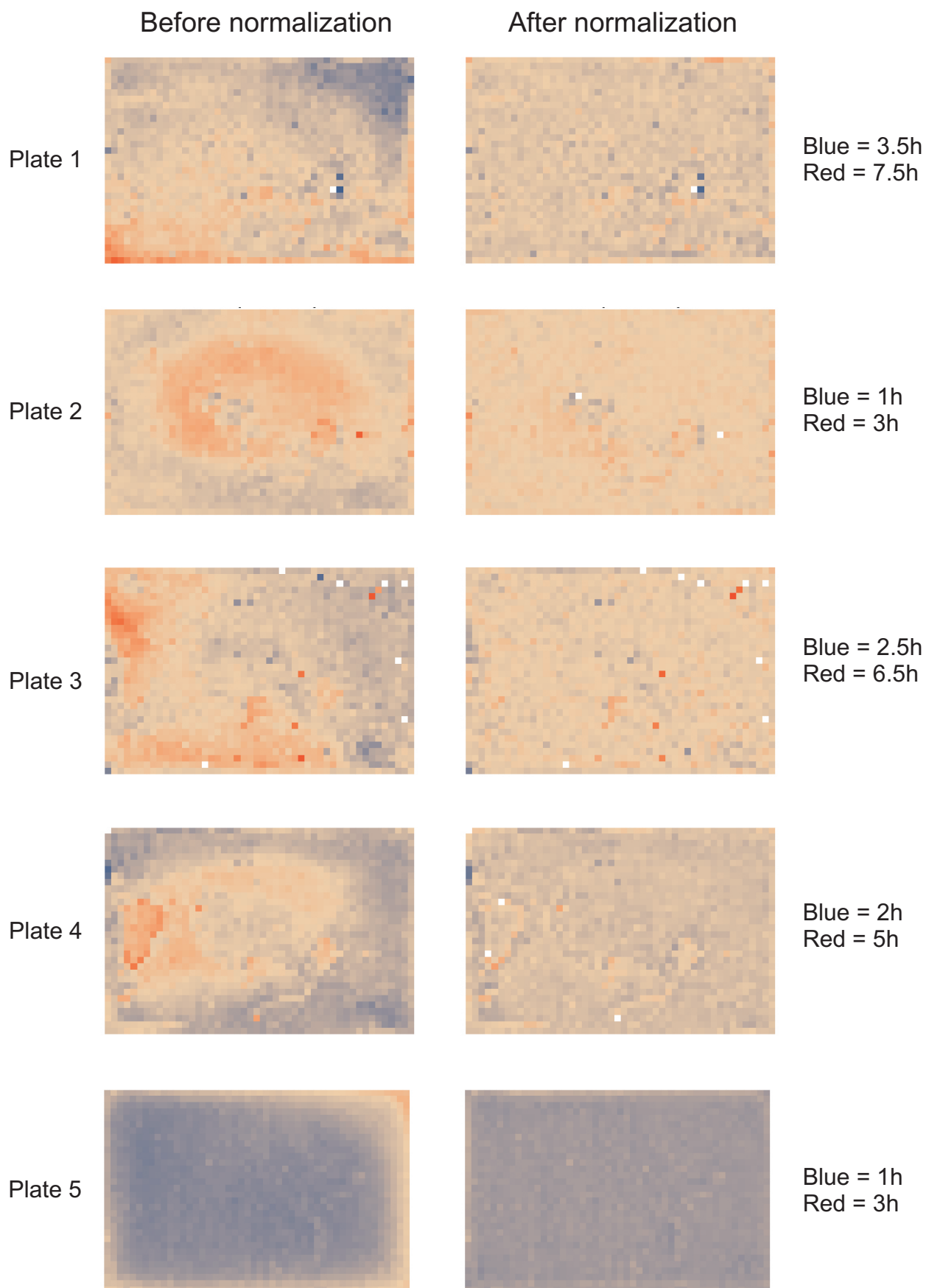

Figure S11 Zackrisson *et al*

Supplement: Supplemental Material [file supp_g3.116.032342_FigureS11.pdf]

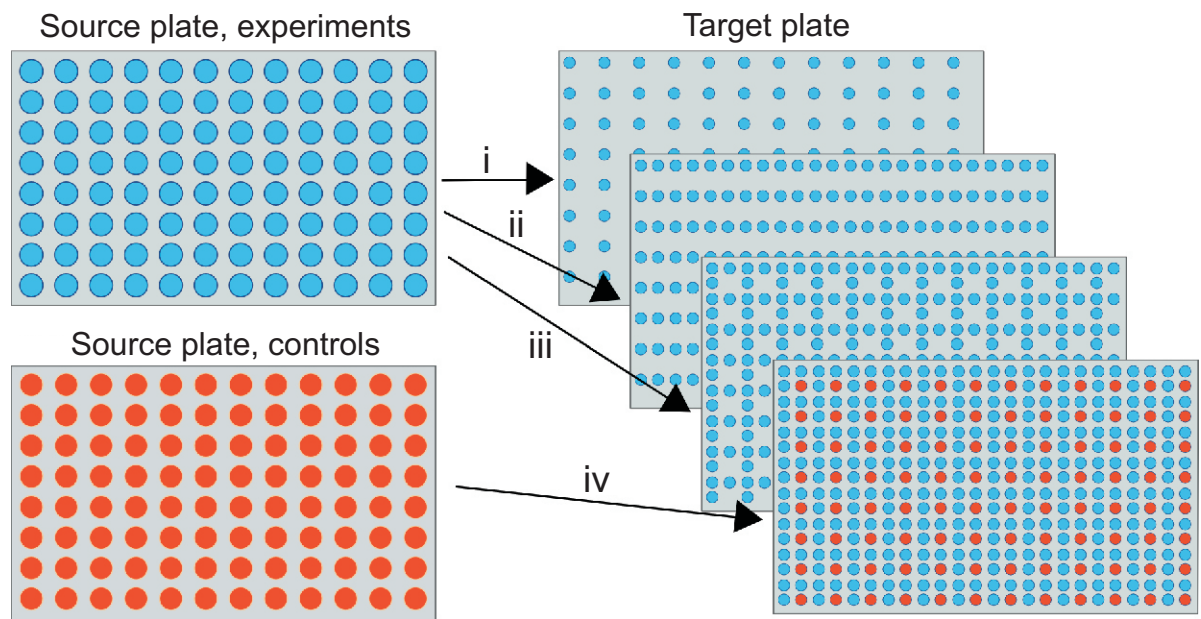

Figure S12 Zackrisson *et al*

Supplement: Supplemental Material [file supp_g3.116.032342_FigureS12.pdf]

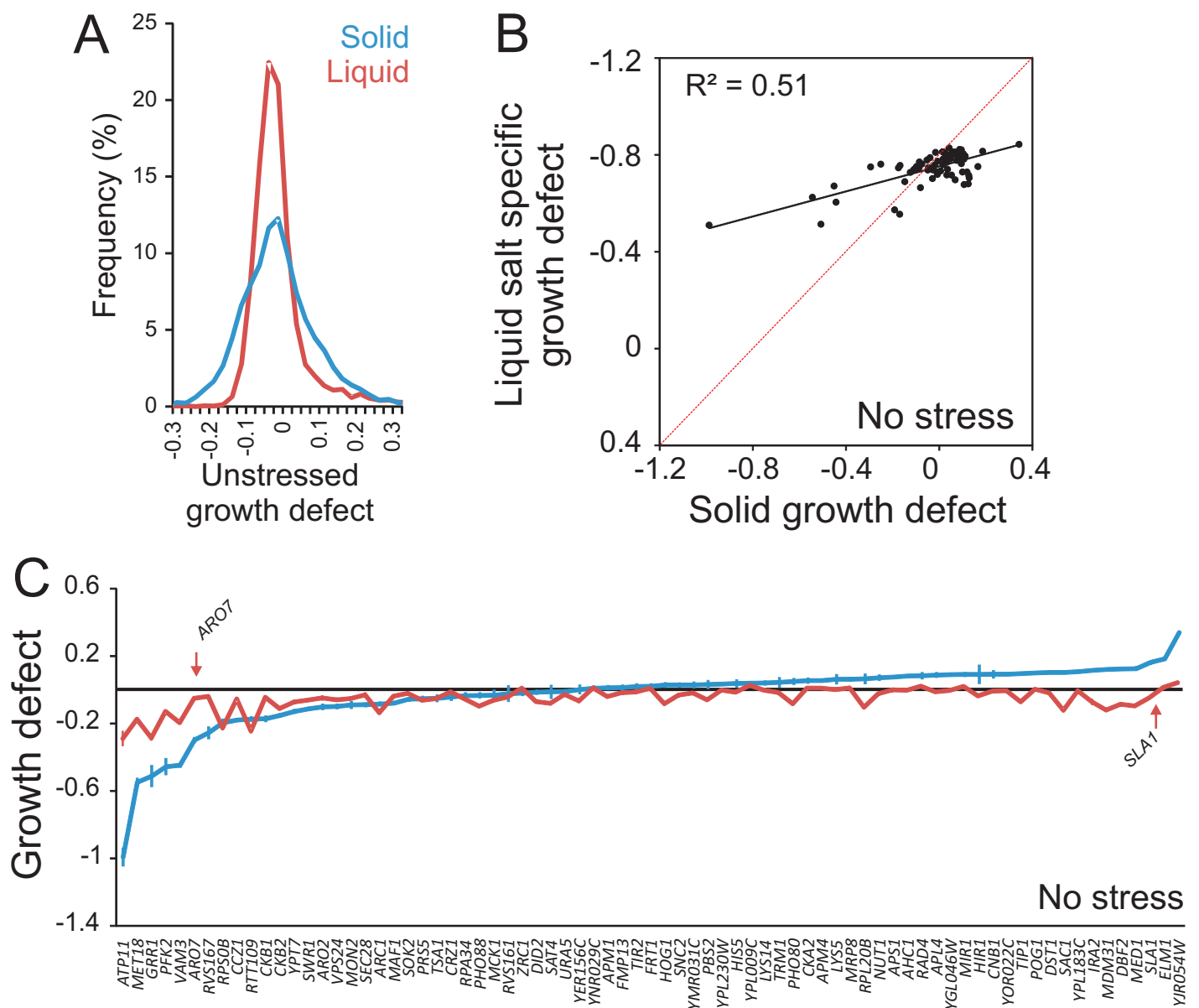

Figure S14 Zackrisson *et al*

Supplement: Supplemental Material [file supp_g3.116.032342_FigureS14.pdf]

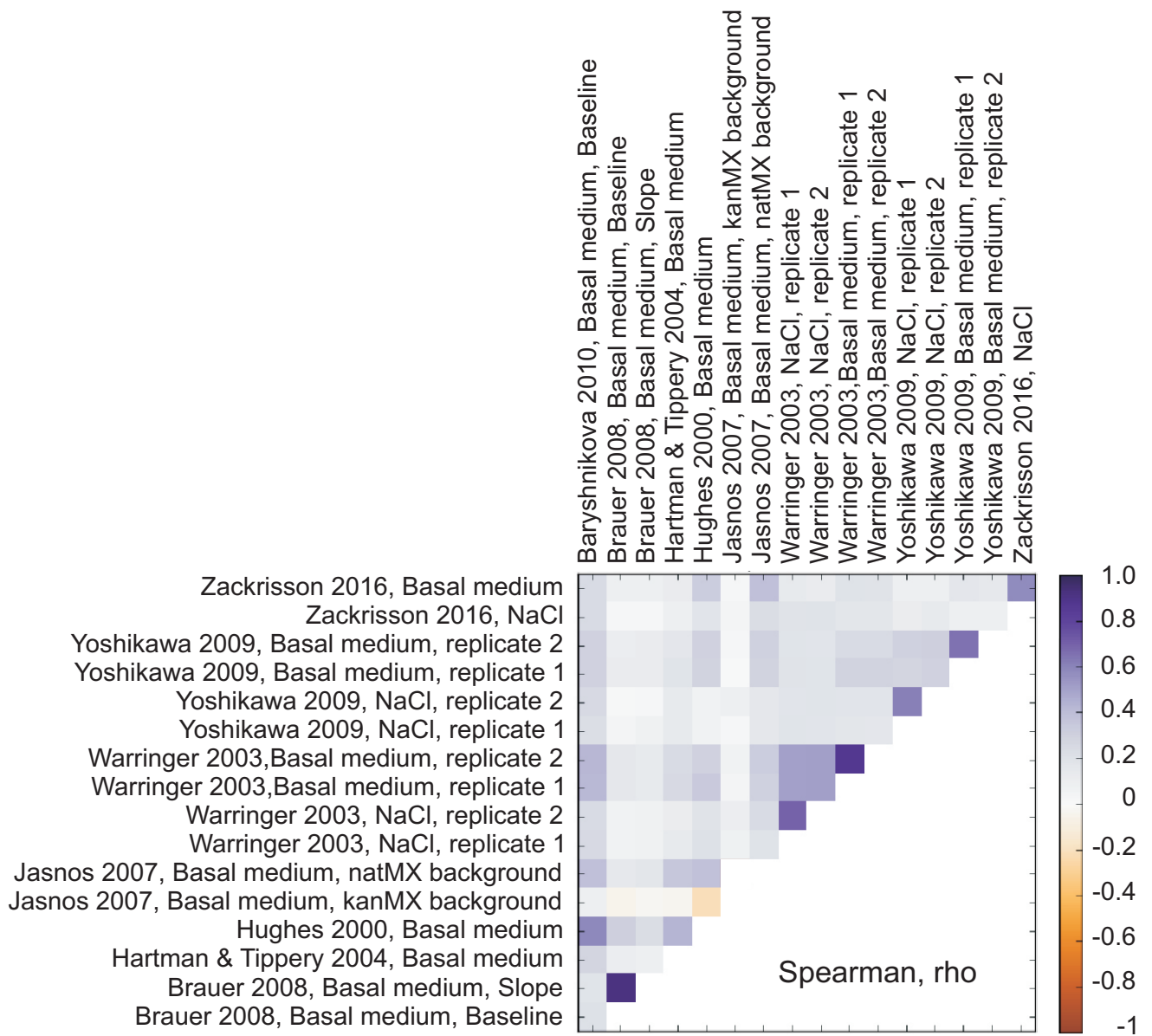

Figure S15 Zackrisson *et al*

Supplement: Supplemental Material [file supp_g3.116.032342_FigureS15.pdf]

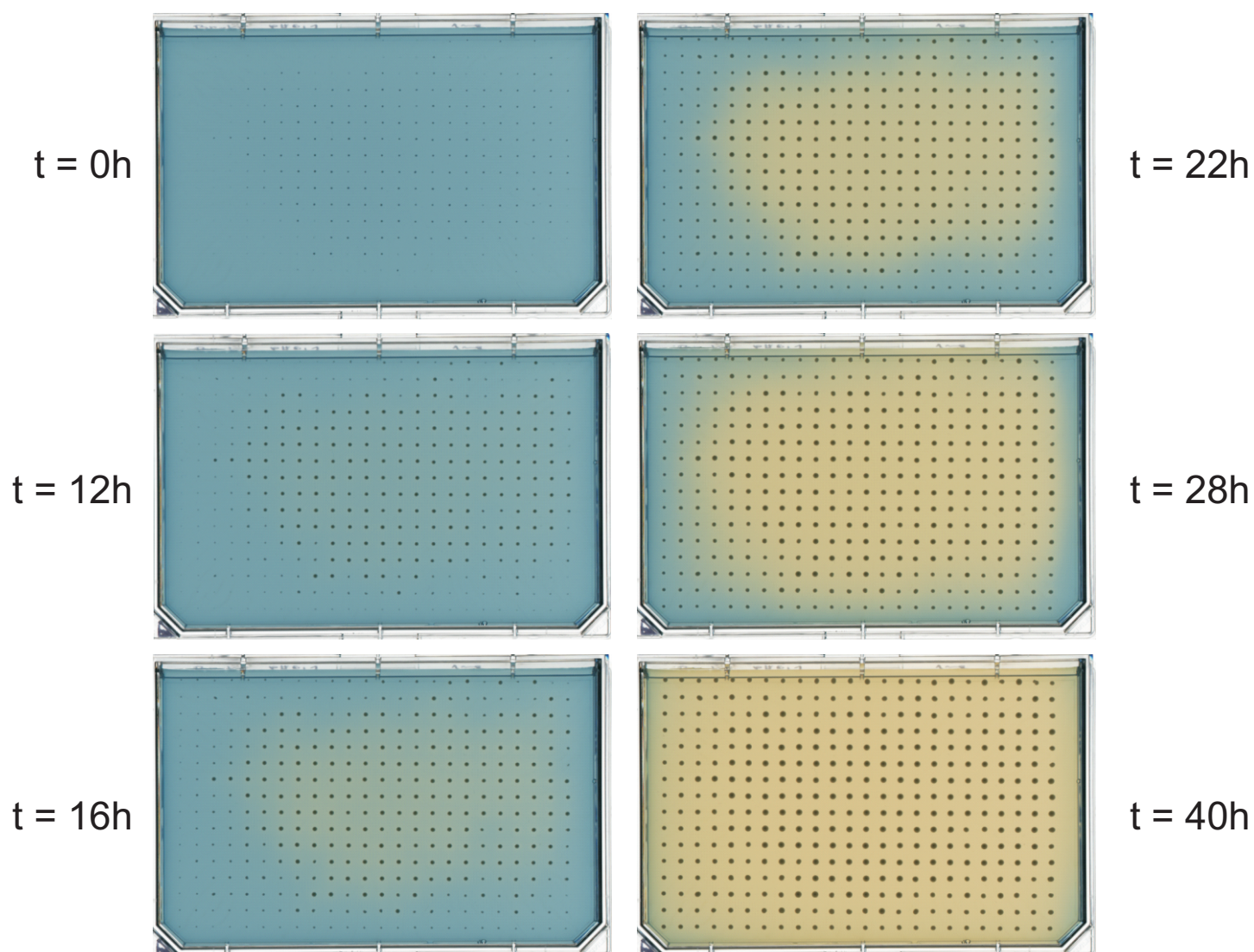

Figure S16 Zackrisson *et al*

Supplement: Supplemental Material [file supp_g3.116.032342_FigureS16.pdf]

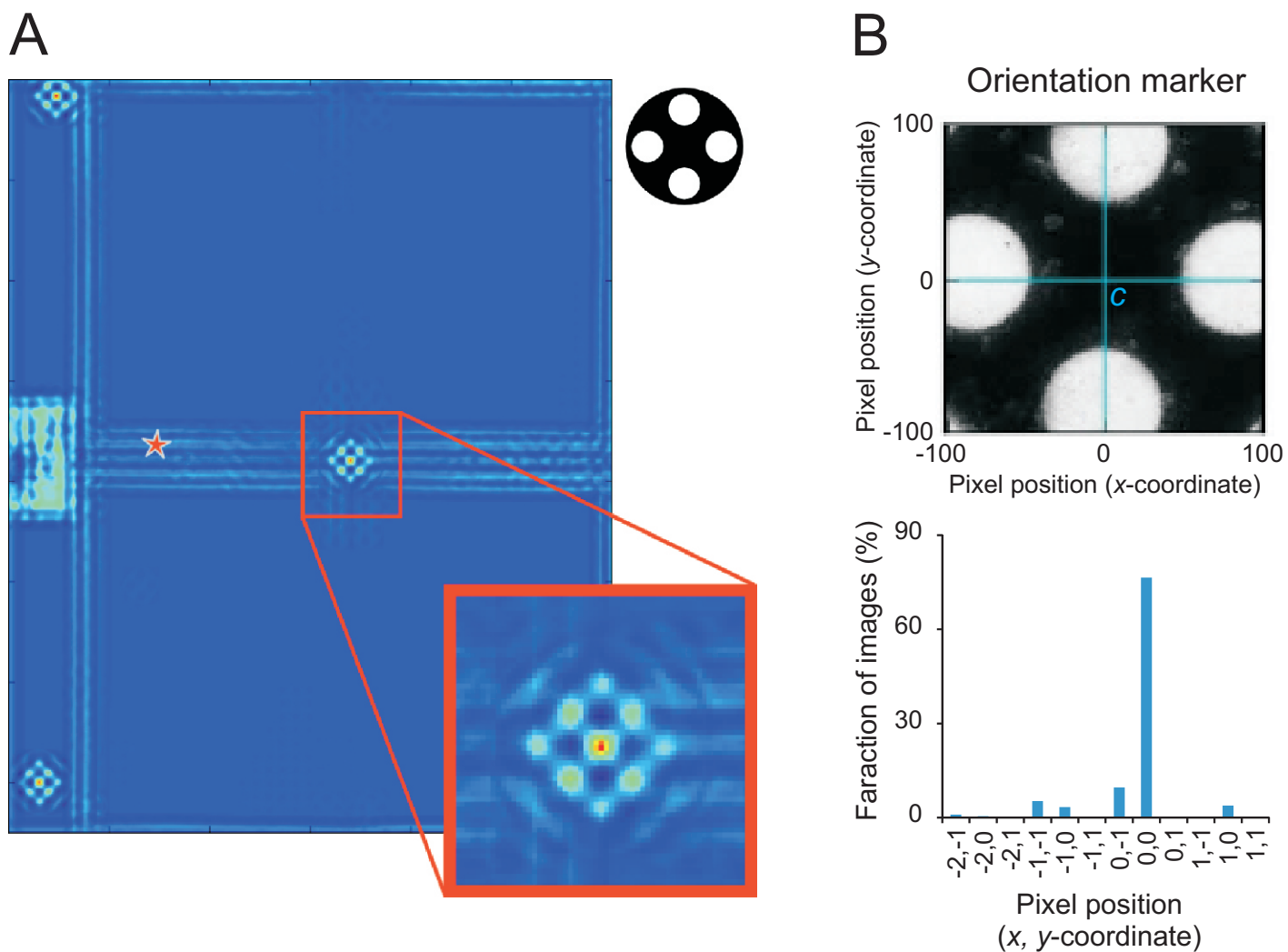

Figure S2 Zackrisson *et al*

Supplement: Supplemental Material [file supp_g3.116.032342_FigureS2.pdf]

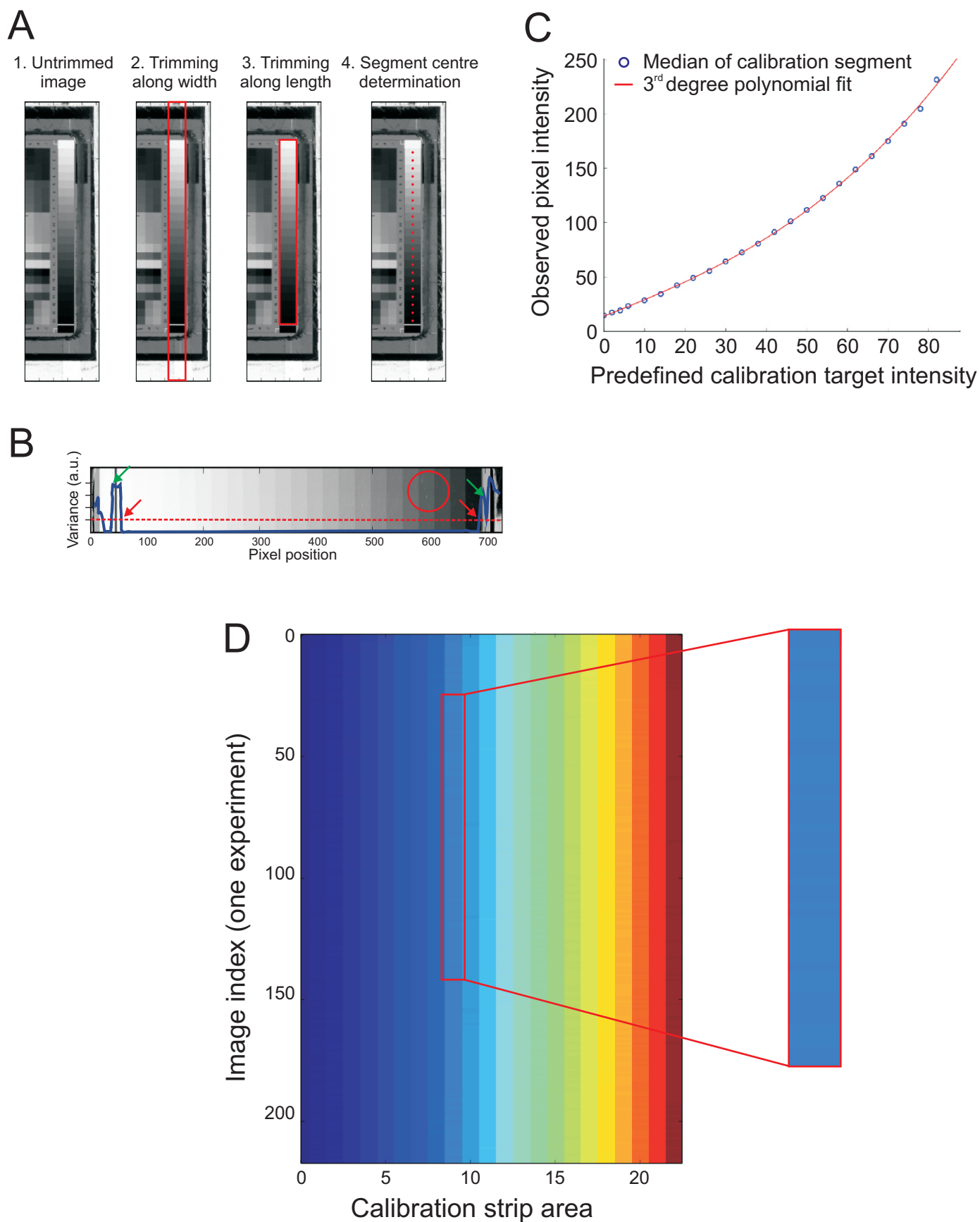

Figure S3 Zackrisson *et al*

Supplement: Supplemental Material [file supp_g3.116.032342_FigureS3.pdf]

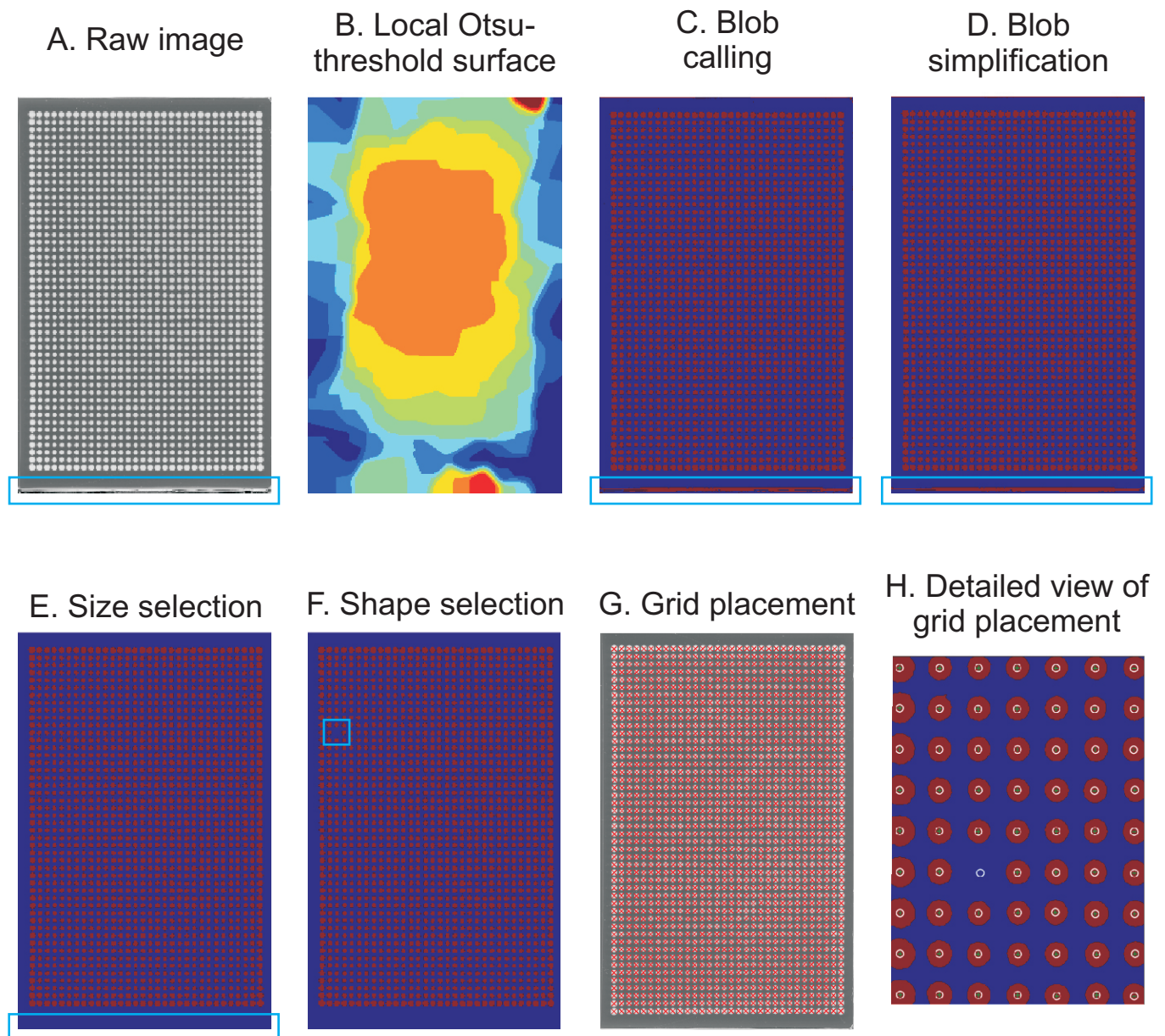

Figure S4 Zackrisson *et al*

Supplement: Supplemental Material [file supp_g3.116.032342_FigureS4.pdf]

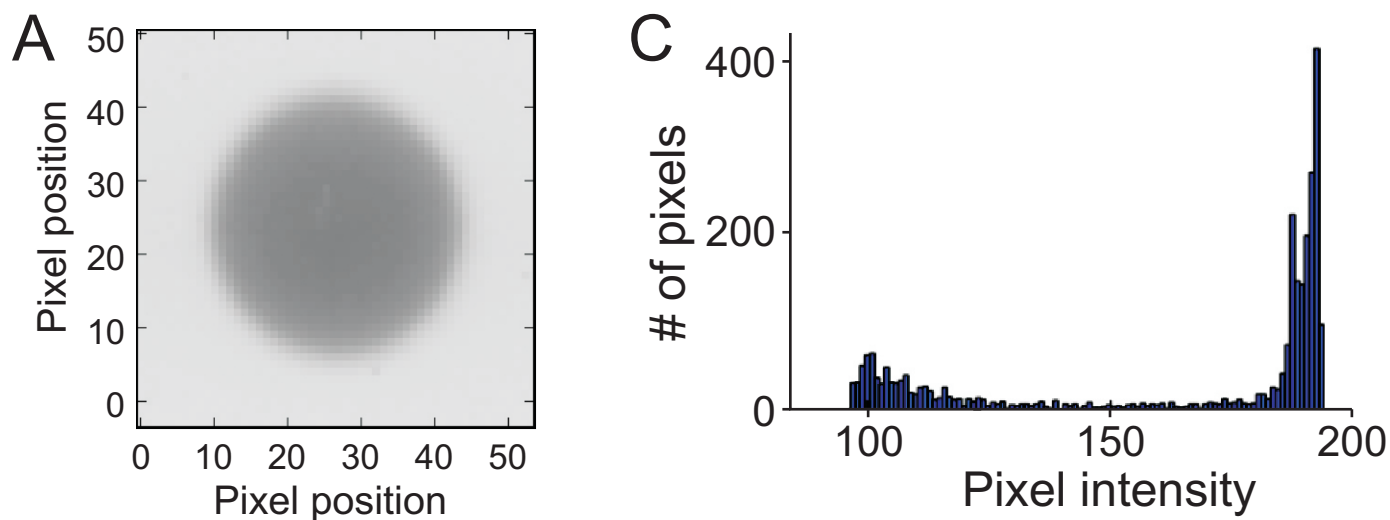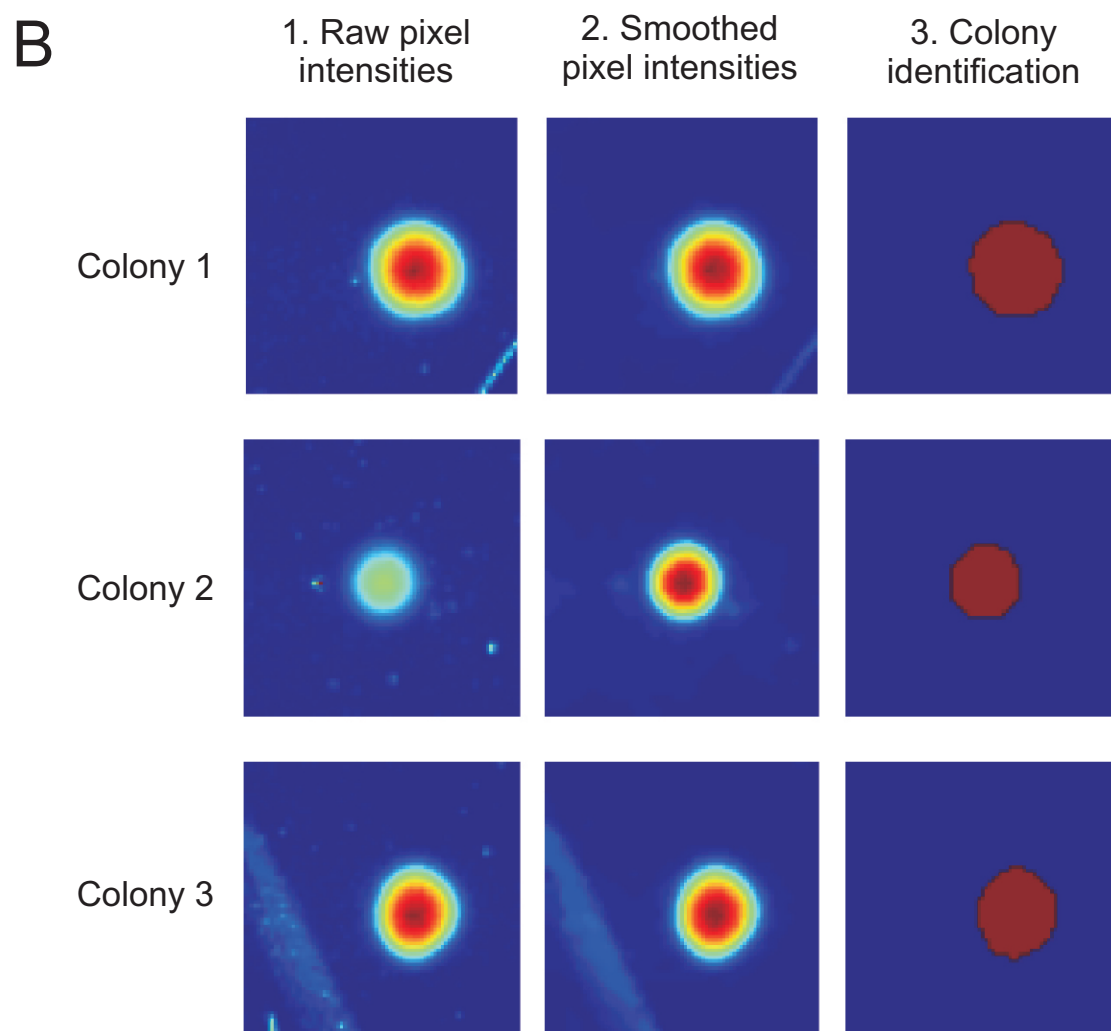

Figure S5 Zackrisson *et al*

Supplement: Supplemental Material [file supp_g3.116.032342_FigureS5.pdf]

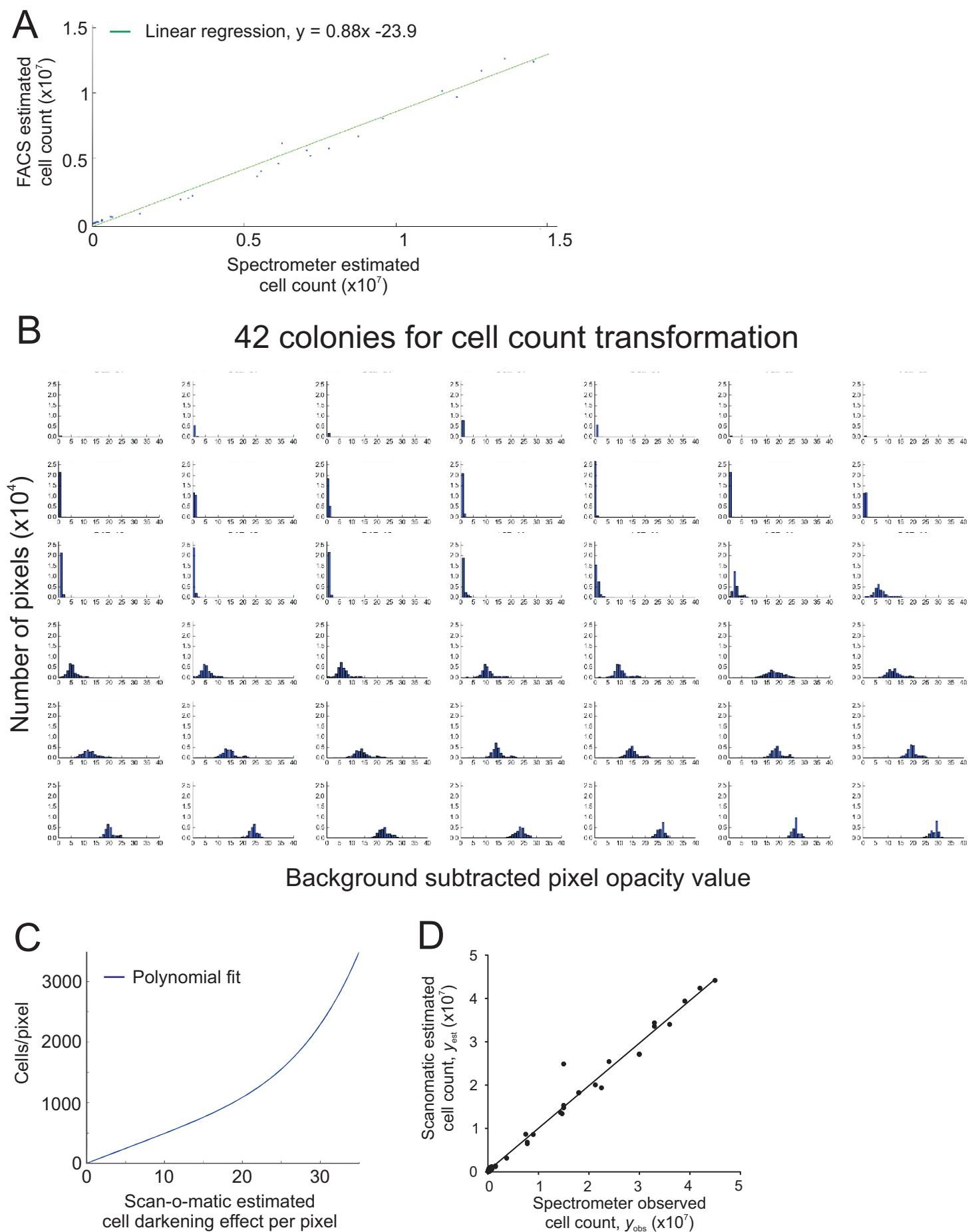

Figure S6 Zackrisson *et al*

Supplement: Supplemental Material [file supp_g3.116.032342_FigureS6.pdf]

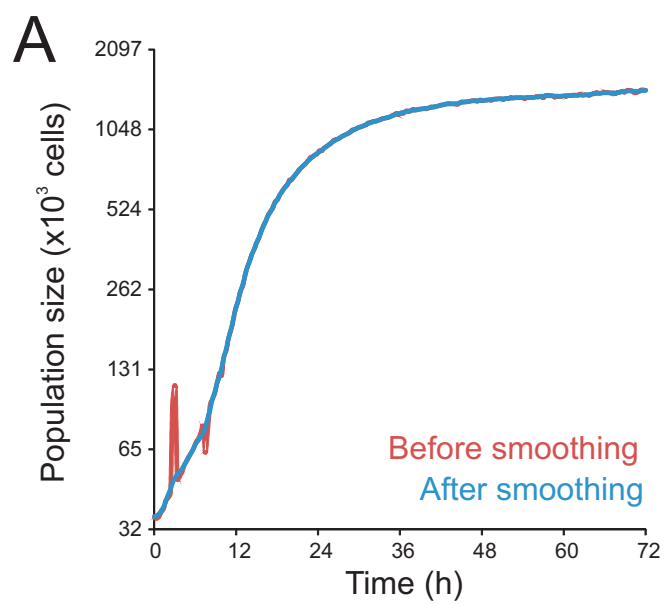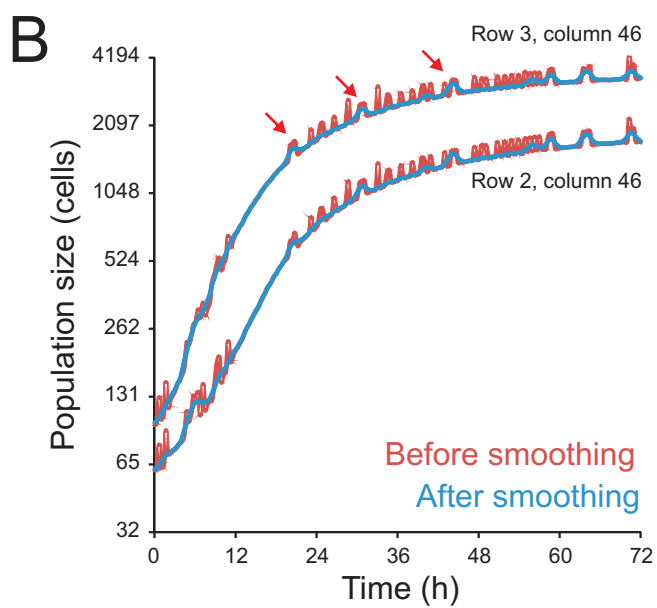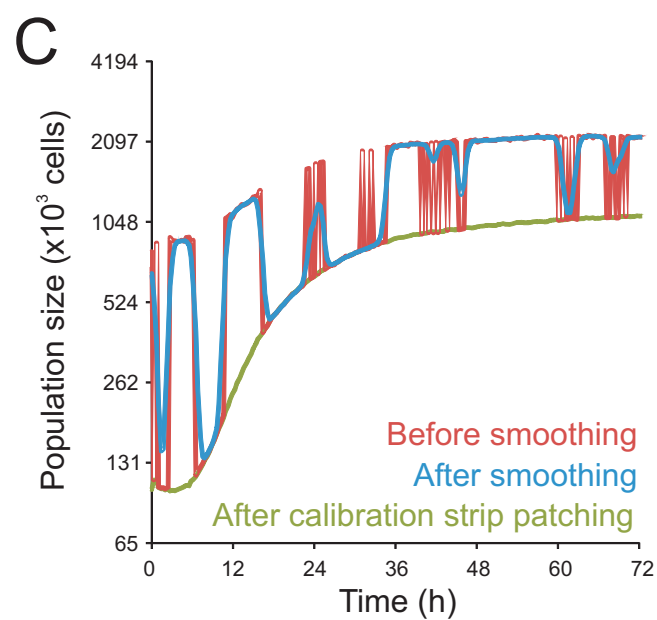

Figure S7 Zackrisson *et al*

Supplement: Supplemental Material [file supp_g3.116.032342_FigureS7.pdf]

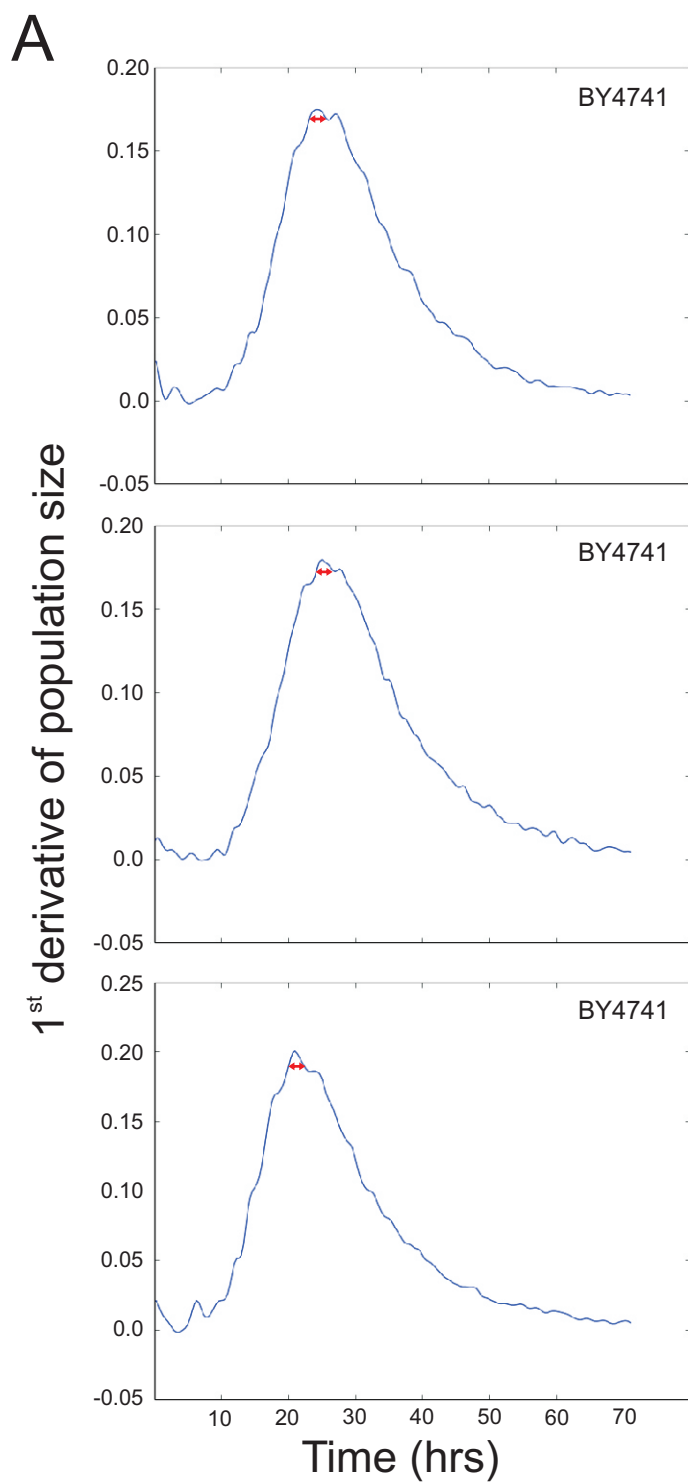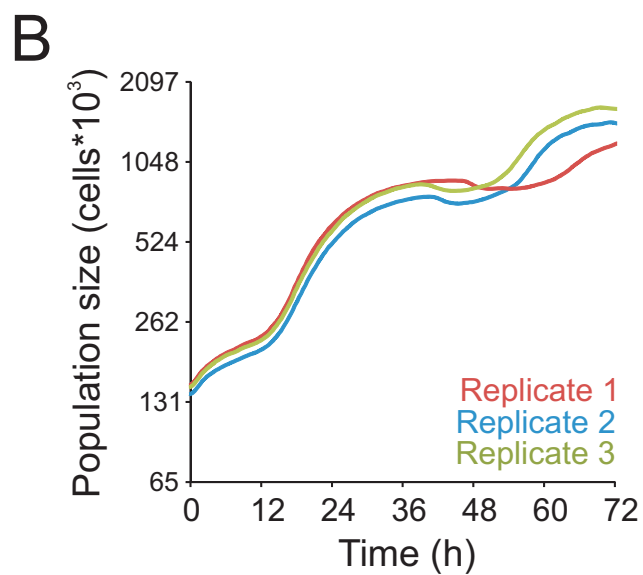

Figure S8 Zackrisson *et al*

Supplement: Supplemental Material [file supp_g3.116.032342_FigureS8.pdf]

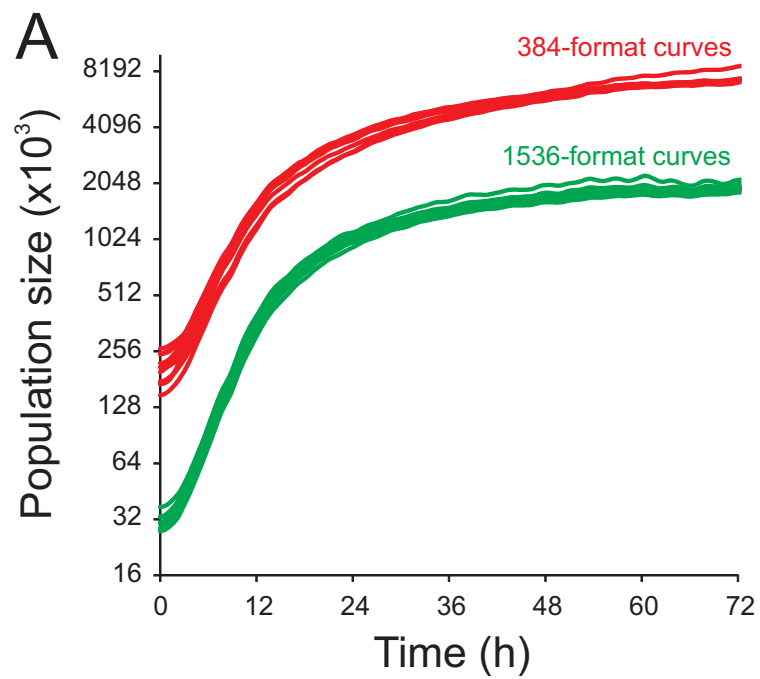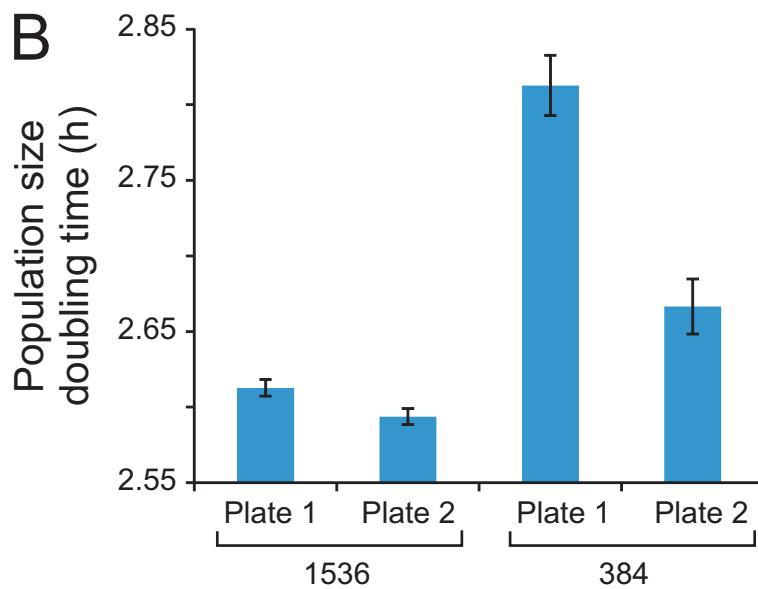

Figure S9 Zackrisson *et al*

Supplement: Supplemental Material [file supp_g3.116.032342_FigureS9.pdf]
