# Supplementary material for: Scan-o-matic: High-Resolution Microbial Phenomics at a Massive Scale
Source: G3 (Bethesda). 2016 Jun 30;6(9):3003–14. doi: 10.1534/g3.116.032342 (PMC5015956; doi:10.1534/g3.116.032342)
Supplement: Supplemental Material [file supp_g3.116.032342_FigureS13.pdf]

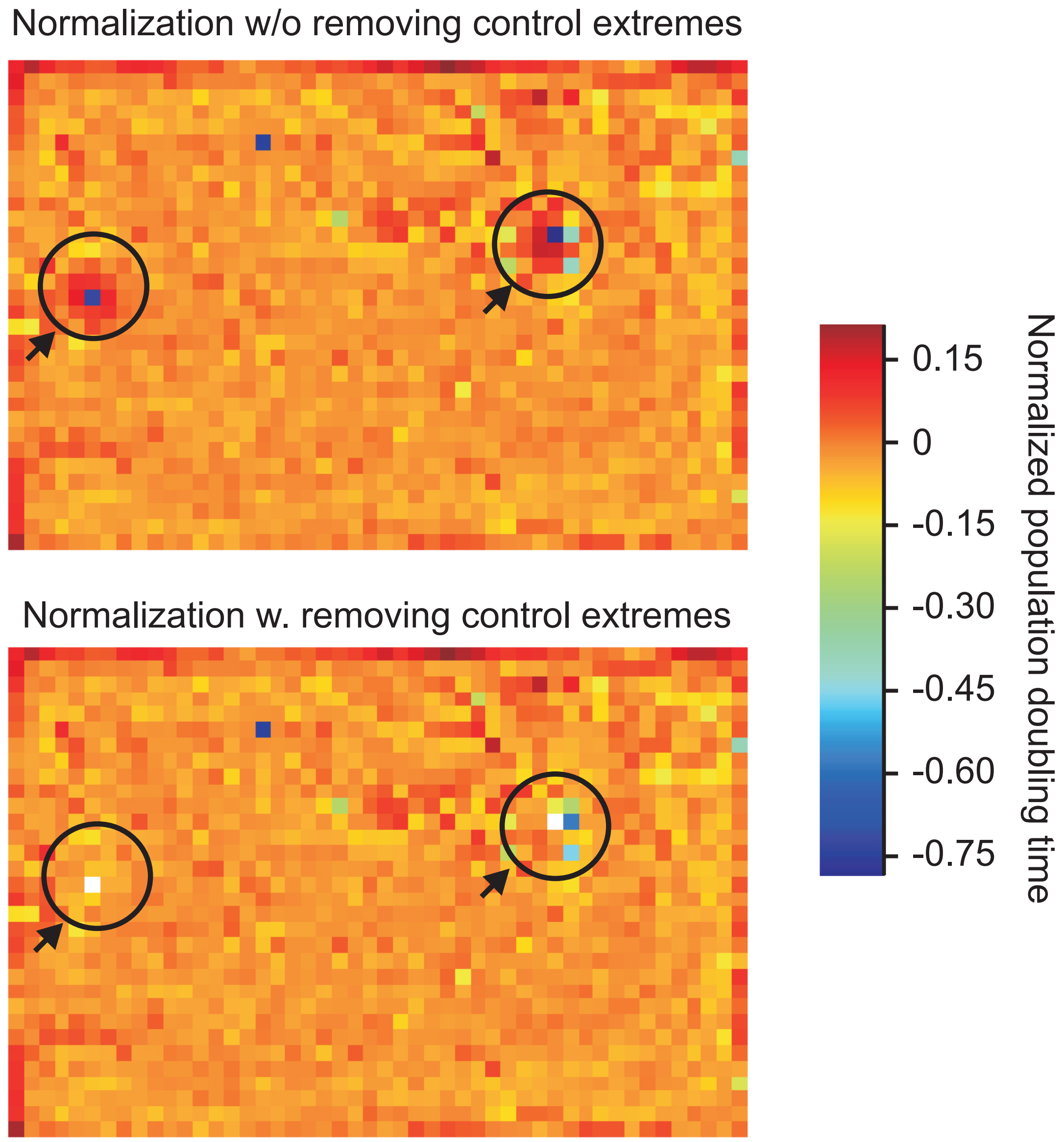

Figure S13 Zackrisson *et al*
