# Supplementary material for: Scan-o-matic: High-Resolution Microbial Phenomics at a Massive Scale
Source: G3 (Bethesda). 2016 Jun 30;6(9):3003–14. doi: 10.1534/g3.116.032342 (PMC5015956; doi:10.1534/g3.116.032342)
Supplement: Supplemental Material [file supp_g3.116.032342_FileS1.pdf]

**File S1: SUPPLEMENTARY MATERIALS AND METHODS**

**Zackrisson *et al***

## 1. Physical arrangement, hardware and hardware control of Scan-o-matic

**1.1 Pinning and plates - standardisation:** Scan-o-matic is based on the use of a pinning robot, which allows for swift and reliable transfer of parts of colonies from source plates to target plates. Dependent on pinning format (pin dimensions), strains and growth conditions, in the range 10,000 – 100,000 cells are estimated to be transferred (Fig S9A). Here, the HDA RoToR robot (Singer LTD, UK) was used but in principle any robot could be used that delivers rather distinct cell-aggregates to the target plate. The plastic, disposable pinning pads (from the same supplier) used by the robot are fixed but not fully rigid 2D matrices of pins that allow transfers in multiples of 96; supported pinning pad formats are 96, 384, and 1536. Scaling-up to higher density colony-formats is done by interleaving repeated pinnings, according to default, as well as custom-made programs (see below). The custom-made pinning programs are specifically designed to introduce an evenly distributed grip of 384 internal control strains in order to minimize measurement noise and spatial bias using the Scan-o-matic normalization principles (see below). Normalization is not a replacement for experimental standardizing experiments (media, plate pouring, pre-cultivation, pinning, humidity, temperature etc.), and the outcome of the normalization depends critically on the quality of the standardization. With regards to pinning and plates, we employ and recommend the following standardization principles:

- All plates and pin pads used in an experimental series should come from a single supplier and ideally from a single production batch.
- All pinning should be done with the same robot, in the same location, with the same pinning program and with the same software version.
- It is critical to create solid medium of even thickness and even surfaces, both for target and source plates. Even surfaces decrease the initial spatial variation in population size that follows from uneven deposition of cells, and decreases local variations in access to nutrients. This will in particular impact on growth later in the growth curve (Fig 2E, F). As even surfaces as possible are achieved by having an absolutely even, and level, surface when casting plates. Note that some causes of uneven medium, e.g. agar

medium creeping up along the walls of plate due to surface tension, cannot be countered in this way.

- To minimize batch bias between plates, it is imperative to pour exactly the same amount (here 50mL) of medium into each plate, to pour into the same position of each plate, to maintain temperature at pouring constant over time and to let plates dry for the same length of time (we recommend 1h) before pinning. If plates are prepared in advance, they should be kept cold, dark, and enclosed in plastic to minimize chemical reactions as well as evaporation.
- The time delay between plate pinning and start of the experiment in the scanners should be kept as short as possible and should not be allowed to vary to much (more than a few minutes) between plates and experimental runs. We pin no more than eight plates (corresponding to two scanners) before initiating scanning.
- Consecutive pinnings from the same section of a pre-cultured colony collect fewer and fewer cells, resulting in systematic differences in the number of deposited cells and thus in initial population size. As initial population size strongly affects maximal growth rate (Fig 3B), the bias effect can be large. The greatest difference is observed between the first and the second pinning. We dispose of the first pinning from a pre-culture plate. We use no more than four of the following pinnings from any pre-cultured colony.
- We pin only from the interior of each pre-culture colony, if at all possible. If pinning is done from the periphery, there is a risk of cross-contamination because of the partial encroaching of larger colonies into the space of smaller colonies.
- Pinning plates are not absolutely hermetically fixed in one position in the robot. The small variation in positioning leads to variation in from which part of a colony pre-cultured cells are collected, more peripheral or more central, and thus variation in the physiological state of cells deposited on experimental plates. To minimize any associated variation, we ensured that the plates were consistently pressed against the same two edges of the frame enclosing the pre-culture plate, when pinning.

Here, these principles were adhered as strictly as possible throughput the experimental series. For further standardization information, see section 8: “wet lab procedures”

**1.2 Scanner:** Scan-o-matic is designed to handle a wide array of scanners as long as they have a transparency mode, are compatible with Ubuntu and that images produced are compatible (dimensions, format) with the analysis-pipeline. The grey-scale calibration (see 1.3) ensures that differences in scanner properties have minimal effect on growth curves generated. Here we used Epson Perfection V700 PHOTO scanners (Epson Corporation, UK) but the current version of Scan-o-matic performs identically with e.g. the Epson Perfection V800 model. Adapting Scan-o-matic to handle other models (e.g. the Epson Perfection V1100) should offer few challenges. Three scanners are connected via USB to each controlling computer (Fig S1A, B). The power supply of each scanner is controlled individually and independently from a computer using a GEMBIRD EnerGenie PowerManager LAN (Gembird Ltd, the Netherlands) (Fig S1B). The power manager, by reducing fluctuations in electrical power, also ensures that the lamp temperature is stable over time, reducing bias and increasing the life expectancy of the lamp. Each experiment (each scanner) is run as an autonomous process, but scanner power supply toggling is coordinated (scanners are fired up at different time points; see below). This ensures that malfunction in one scanner does not affect other scanners connected to the same computer. Experiment processes do not require the user interface to continue running for their operation. Images are acquired using SANE (Scanner Access Now Easy), a standardized Linux interface for scanning (Mosberger 1998). Scanning is performed using transmissive scanning with the transparency unit (TPU) fixed at 600 dpi, 8 bit grey scale. The V700 has two TPU modes. The officially documented TPU mode is unable to support scanning of the full scanner area, and can only capture two plates in each image. To enable simultaneous capture of four plates and allow sufficient throughput, we updated the back-end for Epson scanning in SANE to support a second TPU mode. This mode captures the full scanning area, i.e. recorded images can contain four plates. The second TPU mode is available from Ubuntu 13.04 onwards; for that reason, we recommend using the long-term release Ubuntu 14.04. The scanner firmware lacks control options for shutting down the scanning lamp rapidly after image acquisition. This causes the lamp to stay on for >15 minutes after scanning. Extensive light exposure leads to drying out of the solid agar medium, contraction of the agar and consequently colony repositioning. It also causes excessive and spatially biased variation in radiation, water evaporation and temperature. The spatial bias in these respects is due to that the lamp is positioned at one end of the scanner between scans. The effects combine to create severe

spatial bias. Furthermore, the unnecessary lamp-use leads to faster deterioration of lamp and sensor properties over time. This deterioration in scanner properties leads to peak shifts in image histograms and associated loss of measurement precision and bias. To minimize these effects and taking into account EU regulations regarding stand-by mode in electronic appliances, we physically modified scanners by insertion of a plastic wedge behind (and thus short-circuiting) the power button of each scanner. Scanners are therefore ready to scan when given power. To allow instant shutting down of the scanner lamp after image acquisition, scanner power supplies were connected to a GEMBIRD EnerGenie Power Manager LAN, a programmable power manager with an internal web-server. The Scan-o-matic software automatically logs into the power manager over the local area network, turning on and off power-sockets via its web-interface, as scanners are needed. Further complicating scanner control, individual Epson V700 and V800 scanners lack unique identifiers. Thus, there is no default way of distinguishing multiple scanners connected to the same computer. Scan-o-matic instead tracks the provision of power to scanners, ensures that scanners are provided with power in a serial manner (i.e. they are fired up serially but scanning proceeds simultaneously), and links the order of power provision to assignment of USB ports.

**1.3 Computers:** Scan-o-matic is run on standard desktop computers with the following suggested minimum requirements: Linux operating system, 2 GB RAM, Intel Core i3, 2TB storage. The analysis segment of Scan-o-matic (described in section 3 and 4 below) also works on Macintosh computers. Correct functioning in a Windows environment is currently not ensured.

**1.4 Plate fixture, orientation markers, and grey-scale calibration strip/target:** Each scanner has an individual custom-made CNC (Computer Numerical Control) milling machine-cut acrylic glass fixture with four slots, each slot designed to take one plate (in our case Singer Plus plates; these are slightly larger than ordinary standard plates). Each fixture can be used in any scanner but must be calibrated to that scanner before use. Fig S1A shows a scanner with a fixture in place. Each slot has pegs along its edge to ensure that plates are inserted with a fixed orientation, an otherwise common human mistake. A graphical representation of the fixture that can be used as a template for cutting is available at https://github.com/local-minimum/scanomatic/wiki/Fixtures. Fixtures have three orientation

markers (Fig S2A). Orientation markers can be downloaded from https://github.com/local-minimum/scanomatic/wiki/Fixtures. These are used to triangulate the position of the plates and the calibration target within the fixture, both of which are initially unknown to the computer. The three orientation markers allow precise identification of plate and calibration target positions, thus avoiding any bias arising from slight movement of the fixture between scans. Each fixture also has a slot where a scanner calibration target, containing a transmissive scale, is inserted. The grey-scale calibration strip is necessary to normalize pixel intensities between scanners, as well as to account for changes in the property of lamps, sensors and the space between them (dust, scratches, and light conditions) within and between experimental series. In the present work, the transmissive scale Kodak Professional Q-60 Color Input Target (Kodak Company, USA) was used as a calibration target (grey scale). However, the framework also supports calibration targets from SilverFast (LaserSoft Imaging AG, Germany). Pre-set grey-scale values are available for these types of strips in Scan-o-matic. They work equally well and cover the same pixel intensity range, but the number and intensity of individual calibration strip segments differ somewhat. The operator indicates the position of the calibration strip in the fixture calibration model. The calibration target must be carefully fitted such that the whole calibration strip is contained within the slot and unperturbed by the borders of the slot, and such that it is aligned perpendicular to the movement of the lamp. Failure to accurately fit the calibration leads to severely distorted experiments. A typical example of this is shown in Fig S7C.

## 2. Scan-o-matic software

Scan-o-matic is written in Python (2.7). The user interface is currently being migrated from GTK 2.0 to a more flexible and maintainable HTML5/JavaScript front-end Flask server solution. The modules NumPy, SciPy and Scikits-Image (van der Walt et al. 2011; van der Walt et al. 2014) are used for computation and analysis (http://www.scipy.org/). Information about full suite of dependencies is bundled with the application and is available at: https://github.com/local-minimum/scanomatic/wiki/Installation.

## 3. Scan-o-matic analysis pipeline

### 3.1 Design of a custom-made robotic pinning program for the Scan-o-matic pipe-line:

Experimental standardization (see section 1.1 and 5.2) can reduce, but never completely

remove, spatial bias within plates. A primary objective in the Scan-o-matic design is to minimize any lingering spatial bias, despite the measures that have been taken to achieve standardization, at the analytical stage. To this end, the Scan-o-matic design sacrifices experimental positions on each plate to introduce a dense and even reference grid of control positions. Growth differences between these control positions capture the spatial bias across a plate. The array of control positions is therefore used to establish a growth normalization function (control-surface) that is used to subtract the spatial bias from the experimental positions. If controls are handled in a strictly Standardized way and therefore behave constant over time, they also allow for normalization between plates and between experiments. See 5.2 below for further information on spatial bias and the associated normalization. Controls are interleaved with experiments as outlined in Fig S12, using the following design:

- Two source plates are used, denoted A (or "strain source plate") and B (or "control source plate"). Strain source plate A contains the strains for which the experiment is designed and that are to be analyzed. The control source plate B is a plate with the control strain in all colony positions.
- The strain source plate A is pinned to three different, but neighboring, offset positions onto the target plate, C.
- The control source plate B is pinned once onto a single position of the target plate, C, placing a control colony in the fourth position in each tetrad of positions.

Thus, the target plate C, will contain three replicates of each strain of interest, with the fourth position in each tetrad of positions containing a control strain replicate. Observe that a merging of the two source plates A and B into a target plate C should always occur in the pre-culture, or before the pre-culture step, such that C is equal to the actual experiment plate placed in the scanner. Otherwise, control position spatial bias will not equal experiment position spatial bias and the normalization process loses much of its power. Note that although we recommend normalization by interleaved controls, Scan-o-matic does not explicitly require the use of this particular experimental design and imposes no particular constraints on the arrangement of strains.

**3.2 Fixture calibration:** For exact identification, delineation and quantification of colony population sizes and their local background, experimental plates must be fixed in the same position throughout an experiment. This is achieved using fixtures. Without a fixture, exactly

localizing the pixels covered by a colony would be extremely challenging. Failure to accurately assign pixels to colonies introduces random noise to population size estimates. Each fixture fixes four plates in place (Fig S1A,C) such that the center positioning of their 6144 colonies (4 x 1536 format) is constant and precise during an experiment. Before a fixture is used in a particular scanner for the first time, a spatial calibration model that describes the layout of this particular fixture must be created using the Scan-o-matic fixture calibration user interface. On a scanned image of the fixture, the number of fixture orientation markers, plate positions and the position of the transmissive scale calibration strip are marked by interactive click-and-drag mouse maneuvers. The marking need not be exact, but each marking must contain the complete feature to be captured and no part of any other plate. Scan-o-matic uses this fixture calibration as a model to automatically section images of said fixture into their meaningful components, discarding irrelevant image information and thereby greatly reducing the complexity of the image analysis, computation time, and errors. This is a one-time effort which takes about a minute. The same calibration model can thereafter be used for all experiments with the same fixture-scanner combination.

**3.3 Initiating a Scan-o-matic experiment:** Experiments are initiated from the Scan-o-matic user interface. Here, scanning parameters are set and meta-data identifying the experiment is supplied. Scanning parameters are: scanner (identity) number, fixture number, experiment duration (from 14 minutes to 7 days, but typically 2-3 days), time interval between consecutive scans (from 7 to 180 minutes but typically 20 minutes), and the pinning density of each plate (96, 384, 1536, 6144). The user is also asked to document the contents of the experiment. The experiment is automatically assigned a unique identifier and is initiated from the interface. It will run autonomously until completion. Results are automatically stored and analyzed.

**3.4 Image Analysis:** Analysing high-resolution images and converting them into accurate estimates of colony population sizes is computationally intensive. To separate this analysis from image acquisition, and to prevent it from interfering with image acquisition, we therefore run the image analysis procedure after all images in an experiment has been acquired. This also has the advantage of allowing us to analyse images in a time-reverse manner, starting with the last image in which colonies are largest and identification of their

center positions most precise. During the period of the image acquisition, we only perform the most basal analytical tasks: identifying the fundamental features (calibration strip, orientation markers and plates) of each image. Thus, the analysis is separated into two passes:

- A computationally cheap 1$^{st}$ pass analysis that is run while the image acquisition is progressing (schematically illustrated in Fig S1C).
- A computationally intensive 2$^{nd}$ pass analysis, run after the whole experiment and image acquisition has been terminated (schematically illustrated in Fig S1D).

**3.4.1 1$^{st}$ pass analysis - image registration:** The 1$^{st}$ pass analysis serves to register where the different features of the image are localized. Thus, it finds and annotates the orientation markers, the grey-scale calibration strip and the plates.

**3.4.1.1 Localizing orientation markers in each image:** A basic requirement for analysis of image time series is that correspondence between certain features in these images can be established over time. Typically, correspondence and consistency is achieved by including one or more easily recognizable symbols on the object to be imaged. These symbols, here referred to as orientation markers, tend to be symmetrical and depicted in opposite color extremes (i.e. black and white). This maximizes contrasts, both between fields within the symbols and between the symbols and their surroundings. The positions of the orientation markers can be defined with high accuracy in each image. Orientation marker positions can therefore be used to align image features, ensuring correspondence, and consistency, over the full sequence of images in an experimental run. In the Scan-o-matic design, three orientation markers are included on each fixture (Fig S2A, inset). The first step in the image registration is to localize these fixture orientation markers within each individual image. This is done in the following way:

- Each image, $I$, is thresholded with a heuristically set pixel value of 127 (8-bit value range midpoint). The threshold ensures that pixels are classified as either black or white.
- The thresholded image is then convolved, using a fast Fourier transformation function, with a black and white representation of the fixture orientation marker, $M$ (Fig S2A). The values of the image $I$ and the marker $M$ is set to range from -1 to 1. This

evaluates, for each pixel of the image, how well the local context of that pixel matches an ideal representation of a fixture orientation marker.

- Iteratively, with the number of iterations corresponding to the number of fixture orientation markers in the fixture model (i.e. three on the fixtures used here, but Scan-o-matic allows for any larger number as well) the strongest signal position is selected and chosen as the candidate center position, *c*, of that fixture orientation marker.
- Using the local signals around *c*, with local defined as the size in pixels of marker *M*, the candidate position is refined in an iterative process (<20 iterations; for termination see below). The refinement is achieved by iteratively calculating the mass (pixel intensity) center of the local image section; because of the absolute symmetry of the orientation marker its true mass center defines its true center. The convolution signals are used as weights, after the weights have been adjusted by multiplication with a 2D Gaussian function centerd on the candidate center position, *c*. When constructing this Gaussian, its width (sigma) is set such that magnitude = 0.5 magnitude correspond to a distance that is half the distance of *M* from *c*. The new mass center calculated is set as the true *c*. Note that this is done with sub-pixel precision. The iterative refinement is terminated if the squared distance between $c_i$ and $c_i + 1$ is less than 0.0001 pixels.
- Because the fixture includes several markers (here three), there is a risk of calling one of the markers several times, rather than each one once. To avoid reporting the same marker twice, the local area around a detected *c*, corresponding to the size of the convolution kernel *M*, is set to very low values (one less than the minimum of the entire convolution surface) before the process is iterated. This makes it impossible for the same marker to be called more than once.
- The process of marker localization is repeated on the modified surface until all markers have been found.
- The center coordinates of the orientation markers (*c*'s) are stored as a single output data set, *C*.

Fig S2B shows one random orientation marker and the consistency with which its central point, *c*, is identified throughout an experimental time series of images. The pixel identified as containing the center point in an image rarely varies with more than one pixel position across images. Note: when substantial variation across images begins to manifest in the assignment of c's, this is typically a signal that the mechanics of the lamp positioning of the scanner is

beginning to give up, giving rise to random variation in where the lamp is positioned in consecutive images. This is detectable as growth curve noise, where the position of noisy signals in the curves is shared across one column of colonies. An example of such noise is shown in Fig S7B. Problems in lamp positioning are addressed by retiring the scanner or changing its belt.

**3.4.1.2 Matching image orientation marker positions to fixture calibration model orientation marker positions:** Before further image analysis, the positions of the orientation markers on each image (i.e. the true c's above), must be aligned to corresponding positions in the fixture calibration model in order to pinpoint locations of the plates and the grey-scale calibration strip in the image. The alignment is performed as follows: First, all possible permutations of the three marker coordinates, $C$, of the analyzed image are evaluated for their distance to the coordinates in the calibration model. Second, the permutation with the smallest sum distance is identified and is assumed to describe the correct marker pairing. Third, the offset vector (defined by the marker positions) between the two coordinate systems, image and fixture model, is calculated. This offset vector is then applied to all other plate and calibration strip coordinates of the model in that experimental series. All coordinates for model and image are thereby aligned and their positions stored. The alignment of each image to model coordinates has five important consequences:

- It allows for the complete image registration to be automated, i.e. of plates and the calibration strip.
- It allows exact determination of the positions of plates within each image.
- It greatly reduces the complexity, and computation cost, of localizing, annotating and ensuring correspondence of colonies over time. Specifically, it allows gridding (see below) to be performed once for all images, starting on the last image in each time series.
- Since colonies will be largest in the last image in a time series, this has the added benefit of also making gridding more accurate.
- It further ensures that pixels used to estimate the local background of each colony will be consistent throughout the time series. This increases accuracy.

The use of fixtures ensures that the vast majority of experiments do not have problems with plate movement during the experimental run. The Scan-o-matic default setting is

therefore to use only the image registration done on the last image of the experiment. This simplifies the analysis procedure, while maintaining full automation. If any movement of plates has occurred, it will be immediately detected as concurrent noise to all curves. The analysis can then be re-run using dynamic image registration (meaning that the registration is performed on each image individually; this mode can be set in Scan-o-matic). Based on the matching of the fixture calibration model to each image, all relevant image features (the four plates and the single calibration strip area) are sectioned out and stored for later analysis.

**3.4.1.3 Pixel intensity calibration:** The properties of two instruments of the same type, such as two scanners, will never be identical. Consequently, identical signals recorded by two instruments, e.g. identical pixel intensities, might not mean exactly the same thing. This confounds comparisons of results between instruments. Moreover, the properties of an individual instrument also vary over time. Some of these changes correspond to reversible fluctuations in the environment, such as due to variations in power, temperature, light, humidity, air pressure or dust. Others correspond to irreversible alterations in the instrument itself (wear and tear), due to e.g. spontaneous chemical decompositions in the materials, reactions with light, oxygen and radiation or physical damage, such as scratches. Comparisons between instruments, and between time points, can only be done if the recorded signals are first compared to those recorded to a fixed standard, i.e. to something whose properties tend not to vary, or to vary very little, over time and space. Such comparisons are standard in most empirical sciences and are referred to as calibration. In spectrophotometry, including the here used transmissive scanning with Epson desktop scanners, five types of variation are particularly problematic:

- Variation in the properties of the light source, i.e. how much and what wavelengths of light that is emitted. Lamps vary in their initial properties and these properties deteriorate over time, with trajectories that vary from lamp to lamp, depending both on type of lamp (e.g. LED), brand and the properties of the individual lamp
- Variation in the properties of the sensor. These too correspond to both initial variations between instruments and to changes over time.
- Variation in the amount and type of physical particles in the space between lamp and sensor, i.e. dust.

- Accumulation of scratches and defects on the glass surfaces of the scanner. Irrespective of the calibration, we recommend users to use fine tissues and only approved solvents when cleaning scanners as well as handling the plates and fixtures with care.
- Variation in the lamp-to-sensor distance between instruments. The distance the light has to travel directly influences how much of it that is lost on the way due to reasons other than the imaged object. Even small variations in the lamp-to-sensor distance can have large influences on the recorded signals. As plates are 1cm high, the lid, containing the lamp, must be raised 1cm above its normal position. To avoid variations in lamp-to-sensor distance, the lid must be made to rest perfectly level over all plates. This must be ensured before an experiment is initiated.

Calibration of spectrometers to account for these variations is absolutely necessary. Similar calibrations are done in all spectrometers, often without the user being aware of it, or reporting it in publications. Typically, calibrations is performed either in association to individual recordings (e.g. before the acquisition of an image) or every time the spectrometer is switched on. Scan-o-matic performs calibration of each scanned image independently, to counter potential changes in scanner properties also during an experimental time series. Calibration is performed in a manner that is absolutely standard for image analysis: by inclusion of a grey-scale calibration strip/target. The calibration strip corresponds to a strip with 20-25 areas or segments, depending on brand (see above). Each area contains predominantly pixels of one shade of grey, such that the calibration strip forms a grey-scale. Areas in one end of the strip are close to fully transparent. Areas in the other end of the strip are close to black (no transparency). Some minor variation in shade of grey between pixels in each area is unavoidable. However, given that calibration strips are of high quality and protected from damage by placement in a slot in the fixture, each segment is highly uniform in its transparency. Different calibration strips produced by the same producer are nearly identical and will remain invariable also over time if protected from scratches, tears and discolorations. Thus, each calibration strip represents a set of fixed pixel intensities against which recorded image pixel intensities in a scanner can be calibrated. This accounts for any variation in scanner properties not caught by the scanners' own calibration, both between instruments and over time. Note that the recorded pixel intensities, for 8-bit images, range from 0-255. However, the opacity of the grey scale segments is given by the manufacturer in

a different value scale, where the segments' transmissive values range from 0 to around 85. Thus, recorded pixel intensities of 0-255 are, during the calibration, transformed into values on a quite different scale. For calibration strips to accurately fulfil their purpose five criteria must be fulfilled:

1. All calibration strips must have near identical properties. Except for the choice of calibration strip producer, this is beyond the user to influence. Calibration strips from the producers indicated above live up to this requirement.
2. The calibration strip must be protected from physical damage, i.e. tears or scratches, from dust and from all liquids. If there is any visible alteration to a calibration strip, it must be discarded and replaced. The calibration strip is well-protected from wear and tear by its positioning inserted into a slot in the fixture (see Section 1.4).
3. Each area within the calibration strip must be very precisely defined: any erroneous inclusion of pixels that belong to another area, or the surrounding, may lead to a substantial error in the final colony population size estimates.
4. For each image and each calibration strip area, a single value, accurately representing the underlying true opacity in that area, must be established.
5. From the calibration strip, the relationship between the observed pixel intensities and the fixed, real world standardized transmissive values given by the producer must be established. The relationship must be made for the full value range of pixel intensities possible in the image format.

Finally, calibration substantially reduces random noise. This is shown in Fig 1B. When a scanner digitalizes an image into an 8-bit opacity image, there are only 256 possible transparencies available. If the imaged object is of low complexity, many pixels will have the same digitalized value representation. Further, the real transparency in a pixel will tend to be somewhere between two neighboring 8-bit intensity values. Therefore, very minute differences in the properties of a particular scanner can shift a large fraction of the pixels from one value to the next, in between scans. The calibration function uses over twenty regions of pixels, each representing a known level of opacity, to capture and compensate for such shifts. First, an average of such a region will be shifted less than one pixel value if there is a slight shift in scanner properties because only a portion of the pixels would have shifted value. Second, because over twenty grey-scale regions, covering different levels of opacities, are

jointly used to construct a calibration function, noise from individual region's mean measurements is reduced. Together, these properties give the calibration function a very high accuracy in compensating for sub-pixel intensity step shifts in scanner properties. As a result of this, a growth curve based on the calibrated opacity values will be much smoother than a growth curve based on the raw pixel intensities (Fig 1B).

Sections 3.4.1.3.1, 3.4.1.3.2 and 3.4.1.4.3 deal with exactly defining where the calibration strip is, and where the boundaries for each calibration strip area is, in each image; i.e. they deal with point 3 in the list of criteria above. Section 3.4.1.3.3 also deals with establishing a single value for the pixels in each calibration strip area in each image; i.e. it deals with point 4 in the list of criteria above. Section 3.4.1.3.4 deals with establishing the relationship between the observed and fixed pixel opacity values, for each image; i.e. it deals with point 5 in the list of criteria above. The key steps in the calibration are shown in Fig S3.

**3.4.1.3.1 Trimming the calibration strip image along its width:** The image section identified to contain the calibration strip is only an approximate definition of the exact boarders of the calibration strip: pixels outside the 20-25 (depending on brand) opacity segments are still contained in it. As mentioned above, it is absolutely critical to define each grey-scale segment of the calibration strip exactly, such that only pixels of that area are considered when estimating the area's pixel intensity value. Any errors in defining these segments will confound all pixel intensity estimates on that image. Unfortunately, precisely and robustly defining the boundaries of each calibration strips grey-scale segment is not trivial. The challenge is three-fold:

- There is variation (even if minor) in the recorded intensities of the pixels that belong to a segment, i.e. they are not identical but follow a distribution of pixel intensities.
- Neighbouring areas are quite similar in pixel intensities such that pixel intensity distributions of two segments may overlap.
- In the low opacity end, pixel intensities of the calibration strip areas closely resemble the pixel intensities of the surrounding: i.e. finding the exact end of each grey-scale segment is hard.

The first and third requirement when defining calibration strip segments is the precise removal of all pixels that do not belong to the calibration strip, i.e. to trim the calibration strip image. This is easier along the width of the calibration target because of the uniformity of

pixel intensities along this axis. We therefore begin by trimming the strip along its width (illustrated in Fig S3A; the second panel from the left shows the trimming along the width of the calibration strip). The trimming is done as follows:

1. First, pixel intensities in the calibration target image are normalized and centerd on zero. Normalization, setting the value range from -1 to 1, makes the process comparable between images. The centralization ensures that equal weight is given to the lower and the higher opacity values.
2. Second, a 1D kernel is constructed, using the manufacturer's predefined, real world grey-scale opacity values in combination with the expected length of the calibration strip (in pixels, in the 600 dpi image).
3. Third, this kernel is also normalized and centerd on zero (-1 to 1) as above, so that it corresponds to the normalized image in its value range.
4. Fourth, the normalized calibration strip image is convolved with the normalized 1D kernel. The outcome constitutes a 2D signal representing how much the different image offsets looks like the center positions of the areas in the ideal, real world grey-scale.
5. Fifth, along the width axis, each length-wise maximum in a column of pixels is collected.
6. Sixth, the vector containing the convolved image's column maxima is smoothed. The purpose of the smoothing is two-fold:

- All pixel columns that map to the actual calibration target should in theory produce the same value. However, minute variations when pixel intensities are registered by the scanner tend to make one random column match the ideal slightly better than the others.
- The interest of the process is to find the center along the width axis. Smoothing, with a large kernel (i.e. including many columns in the smoothing), will benefit those positions in the vector with more neighbors with high signals, i.e. with neighbors that belong to the calibration strip rather than to its surroundings. The kernel size can be precisely set forcing the background to contribute negatively to all columns, except the column in the very center of the calibration strip. Thereby, its center can be identified.

Smoothing is done using a 1D Gaussian filter, i.e. the weights given to neighboring pixel columns when determining the smoothed value of a particular column are based

on a normal distribution: the further away they are from a the specific column to be smoothed, the less influence they will have on its smoothed value. The width, in pixels, of the normal distribution, i.e. its standard deviation defining the weights, is set to equal half the width (in pixels) of a standard calibration strip. The areas on each side of the calibration strip have very low signals, i.e. they are nearly fully transparent along the length of the calibration strip. Thus, the smoothing allows the surroundings to cause a relevant decrease in the signal intensity of all positions – except for the center of the calibration strip. The position with the highest value will therefore exactly and robustly coincide with the center of the calibration strip along the width axis.

7. Seventh, the true boundaries of the calibration strip along its width are defined as being exactly half the expected width of the calibration target (in pixels, in a 600 dpi image) away from the identified center. The image section is trimmed at these boundaries.

**3.4.1.3.2 Trimming the calibration strip image along its length:** Trimming of the calibration strip along its length is more complex, because of the lack of pixel intensity uniformity and the poorly defined boundary at the low opacity end. The trimming along the length is illustrated in the third panel from the left in Fig S3A. It is performed in the following way:

1. First, we calculate the local variance of pixel intensity in regions (60% the width and length of the expected grey scale segment sizes). This is done because the part that corresponds to the actual calibration strip positions is expected to be highly uniform in pixel intensities, i.e. have low variation. Borders between two segments will have slightly higher variation, but as the opacity difference between two neighboring sections is still comparatively low, the resulting variance is still low. Comparatively, other features outside the strip will be less uniform. As the image is already trimmed along the width and pixels corresponding to positions outside the calibration target have already been removed, no spurious peaks in variation is expected for the true calibration strip part of the image.
2. Second, we take the row-wise median of the variances reported by the first step. This is to avoid any spurious local variation peaks, such as due to dust or hair. Since the

grey-scale is uniform along its width the operation will conserve the information about the position of grey-scale along its length axis. The row-wise median of the variances of one example calibration strip is shown as a blue line in Fig S3B. The permissible rows, i.e. rows that could potentially belong to the calibration strip rather than its surrounding can be determined from these row-wise medians of variances. Such rows are heuristically defined as rows with a median of variances below a value of about 1000 (it varies slightly between the two types of grey-scale calibration strip supported). The cut-off is indicated in the example calibration strip image (Fig S3B) as a dashed red line. This limit is heuristically set, after observing a large number of outcomes of image analyses of calibration strips. The outcome can be viewed as a True/False Boolean permissible position vector: either a row is considered a potential row of the calibration strip area (True), or it is not (False).

3. We can now begin to defining the outer boundaries of the calibration strip along the calibration strip length. The main challenge is not to miss any of the true calibration strip segments in the low intensity end of the strip, while not including any pixels of the adjacent non-relevant pixels. The challenge is evident in Fig S3B: note the similarity to the surroundings of the segments in the low intensity end of the strip. Potential calibration strip edges are detected as non-zero outcomes of the [-1, 1] convolution of the Boolean vector (True represented as 1, False as -1), i.e. where the vector changes from False to True (signal = -2) or from True to False (signal = 2). The other combinations, True to True and False to False both produce signal values of 0. These non-zero positions are stored in a vector. The first edge is verified to have the sign that indicates a potential edge into the calibration strip area (signal = -2), else all values in the candidate edges vector are shifted one to the right and the first image section index is inserted as first potential edge into a calibration target area at the first index of the vector. This is because the first edge type detected indicated that the image started with a candidate area for the calibration target. The last edge is then verified to have the sign indicating a potential edge out of the calibration target area (signal = 2): else the last index of the image section is appended as potential edge out of calibration target area. The pair of edges that define the calibration strips outer boundaries in the vertical dimension is finally selected as the pair of edges with an edge-edge distance that best agrees with the expected length of the calibration target

used. For the example calibration strip image shown in Fig S3B, the pair of edges chosen are indicated with red arrows.

4. Because the variance is calculated across pixels rows and columns corresponding to 60% of the size of an individual segment, the variance will increase well before the last pixel row of the end segments. This causes a slight underestimation (corresponding to approximately 60% of each end segment) of the calibration strip area defined by the edges called in the previous step. The areas immediately outside the calibration strip may also sometimes (by chance) vary very little along their widths, contributing some additional random error in the edge determination. To avoid loss of the end-segments, and the associated potential skew of the calibration function, we therefore add some pixel rows to each calibration strip end. From the center position between the edges selected in the previous step, we trim at half the expected length of the calibration strip - plus an extra ¾ of the expected length of a single calibration strip segment - in each direction. On average, this leaves slightly too many pixel rows along the length of the calibration strip; i.e. it does include some of the immediately adjacent pixel rows. However, the associated inclusion of some noise is accounted for in the downstream process and is much preferable to the alternative: losing some parts of the end segments of the strip. For the example calibration strip image shown in Fig S3B, the final position for the defined calibration strip end points are indicated with green arrows.

**3.4.1.3.3 Defining calibration strip segments:** Once the center of the calibration strip have been precisely established and the calibration strip image trimmed to only contain the calibration strip, the next challenge is to exactly define where each of the grey-scale segments are in the image. To this end, we analyse the calibration strip image along its length axis. The analysis is illustrated in Fig S3A (the rightmost panel). The process proceeds as follows:

1. First the pixel intensity row mean (i.e. the mean across each row pixels) is calculated.
2. Second, the vector of all row means is convolved with a [-1, 1] kernel. As described (above), such a kernel detects edges among the row means, and such edges should only emerge when going from one calibration strip area to another. The largest spikes in the convoluted signal will represent boundaries between the calibration strip areas.

3. Third, we call candidate boundaries as spikes exceeding a cut-off value of 1.2: this value is heuristically set from inspecting the performance of this and alternative cut-offs on a large number of calibration strips. These signal spikes are trimmed from both sides such that only the most central position of the spike remains, marking the exact boundary between calibration segment areas.
4. Fourth, distances between these signal-centers are aligned to the expectation for distances between successive calibration segment boundaries in a 600 dpi scanned image. Note that the expected distances between calibration segment boundaries will vary between different types of calibration strips. The alignment is done such that total error in the alignment (i.e. the sum of pixel distances between each expected edge position and its most proximal called edge position) is minimized. Rarely, some boundaries are not called, i.e. their corresponding signals were just below the signal cut-off of 1.2. In these rare cases, the positions of the missing boundaries are determined by interpolation based on known distances between segment boundaries, or extrapolation if being the first or last boundary, from those that were detected.
5. Fifth, once the boundaries of all calibration strip segments have been precisely determined, we establish the central pixel of each segment. Note that the center along the width of the calibration strip was already exactly known from the trimming stage, and defined by the width edges of the image section. The center positions along the length of the calibration strip are determined by interpolation using the boundaries of each area. Together with boundary coordinates positions, the center position coordinates form a complete model of the calibration strip area.
6. As a final step, we verify that the complete model falls within the trimmed image area. In the extremely rare cases that it does not, accurate detection has failed: this is then reported and the experiment can be re-run, after re-doing the fixture calibration model.

**3.4.1.3.4 Pixel intensity calibration:** Once a perfect model of the calibration strip area has been established, the next step is to determine the pixel intensity that is representative of each of the 20-25 calibration strip segments. This proceeds as follows:

1. To robustly determine the representative pixel intensity in each area, we first define the central 16% (i.e. the central 40% in each horizontal dimension) of pixels and

consider only these pixel intensity values. The exclusion of pixels close to the boundaries of each area:

- buffers against any slight errors in the definition of these boundaries
- buffers against the case that the calibration strip is slightly rotated in the image

2. Second, these 16% most central pixels of each specific segment are sorted in value order and the mean of the central 50% of the pixel values are used as an accurate and robust representation of the average pixel intensity in the segment. The purpose of forming the mean of these pixel values is to protect against any noise that may distort estimates of the calibration strip segments. Such noise may emerge due to dust, scratches, discolorations, tears, hair and similar. Examples of such effects are indicated by a red circle in Fig S3B. By only considering the central 50% of the value sorted pixels, we buffer against the vast majority of such distortions. Note that the interquartile range, commonly used in signal processing, is not suitable here as the values are discrete and rather few (the pixel intensities in a calibration strip segment are rather uniform). Also note that a mean, for much the same reason, results in better precision than the median.
3. Third, the in this way defined mean pixel intensity for each calibration strip segment is compared to the manufacturer's predefined grey-scale opacity values. The relationship between the observed pixel intensities of the calibration strip segments and the predefined grey-scale opacity values of the same calibration strip segments form a calibration function. The calibration function is obtained by fitting a polynomial function to all 20-25 pairs of values. The polynomial is defined to be a third degree polynomial, $y_i = ax_i^3 + bx_i^2 + cx_i$ where $y_i$ is the predefined grey-scale opacity value of calibration strip segment $i$ and $x_i$ is the corresponding observed pixel intensity value of calibration strip segment $i$. One example calibration function is outlined in Fig S3C. Note that the calibration function is unique for each image. Calibration functions are typically quite stable for different images in the same experimental series, but differences do emerge, even with the most extreme level of experimental standardization. This can be seen in Fig S3D. If substantial amounts of dust enter during a run, if light conditions around the instrument change drastically or if the instrument lamp is approaching the end of its life span – the calibration function can differ quite substantially also across images in an experimental series.

4. Fourth, the outcome calibration, for each image, is used to transform all observed pixel intensities in that image into Standardized grey-scale values, here termed pixel opacity values. Note that the calibration function is established and implemented before any detection and quantification of colonies, preventing systematic differences between instruments and between time points to distort the key parts of the image analysis. Also note that the pixel opacity values have no meaningful unit and no direct interpretation; they must be further transformed into actual cell counts before any direct biological interpretation (such as a colony population size and finally growth curves) can be done. This cell count transformation is done in the $2^{nd}$ pass analysis (see below).

Finally, note that the use of the calibration strip makes the downstream analysis of colony population size independent of variation in scanner properties. As most signal recording devices, Epson scanners do contain a built-in calibration: however, this calibration is only done on maximum transparency (i.e., against air), not the full range of transparencies, and is therefore very far from sufficient to buffer against variation between instruments and between time points. Without sufficient calibration, no comparisons between growth curves obtained with different instruments, or obtained at different time points, should be made. We also caution users that substantial artefacts may emerge also within growth curves, if conditions change e.g. due to changes in background light, if calibration is not performed.

**3.4.2 Image Analysis:** The $2^{nd}$ pass analysis detects and segments colonies, and then estimates the number of cells in each colony. This is done after the experiment is terminated, i.e. after all images in a time series have been acquired. We perform the $2^{nd}$ pass analysis in reverse chronological order, starting from the last image. The reason for this is that colony population sizes are larger in the later images; thus, detection of colonies and definition of their positions becomes more accurate. As the $2^{nd}$ pass image analysis progresses from the last to the first image, analysis of each new image uses the candidate colony location, (i.e. a so-called “blob”), from the previous image and ensures that the mass centers are roughly maintained. This assumes that growth have been more or less symmetrical in the horizontal plane, and allows accurate identification, annotation and segmentation of colonies also in

early images where colonies otherwise are quite challenging to distinguish from background features. The principles for the 2nd pass analysis are outlined in Fig S1D.

**3.4.2.1 Grid placement:** The first challenge for the 2nd pass analysis is pinpointing where each colony is on each plate in the image and to assign each colony with a unique identity. To achieve this, we designed an algorithm for automated, high throughput gridding that require manual intervention based on the following fundamental principles:

a) The colonies are arranged in a symmetric array by the use of a pinning robot (see section 1.1). Missing information about a colony position can therefore easily and accurately be determined from its neighbors. False negatives in the automated detection of colonies therefore carry only a small cost.
b) Retaining features that are not colonies, in contrast to discarding some actual colony detections, has the potential of biasing the assignment of where the rows and columns start. This would offset the complete grid and would result in miss-identification of a large fraction of the colonies. Therefore, false positives carry a high cost.
c) The task of gridding is only to locate the center of each colony position, not to define the shape (the outer boundaries) of the colony (this is done later, see 3.4.2.2). The accuracy in defining the horizontal and vertical dimensions of each colony therefore does not need to be high - as long as the center position remains accurate.

Accordingly, the algorithm is highly conservative in what it classifies as a colony and is less precise than the colony detection step (see 3.4.2.2) in defining what pixels belong, and do not belong, to each colony. To achieve a robust registration of where the colonies are located, we produce a virtual representation of the pinning matrix (specified by the user). This virtual representation is based on the average empirical distance between pins in the pinning format translated into number of pixels, given a 600 dpi image. The virtual representation corresponds to a grid of vertical and horizontal lines, with the line intersections corresponding to expected colony positions. We place this virtual grid on top of each imaged plate and align it to the centers of the features that are estimated to be true colonies. The gridding process is outlined for an example plate in Fig S4. The procedure is as follows:

- First, each raw image of a plate is partitioned into random (number, size and shape) sections. Fig S4A shows a raw image of the example plate. The partitioning is done using a standard, randomly seeded Voronoi diagram. Partitioning is essential because

background distributions of pixel intensities vary across an imaged plate, due to variations in medium thickness and coloration, bubbles in the medium, scratches on the plastic and the glass, discoloration in the plastic and on the glass, the inclusion of plate edges and parts of the fixture in the image, and because of specks of dust and hairs. The partitioning of sections is random not to introduce biases: we know very little beforehand about how the described variations distribute across any individual image.

- Second, each section is Otsu-thresholded (Otsu 1975). The Otsu-thresholding can be explained as follows. For each section, the pixel opacity values of the background pixels form one Gaussian distribution whereas the pixel opacity values of the colony pixels form another Gaussian distribution. Otsu-thresholding defines, for each section separately, a threshold pixel opacity value that must be exceeded for a pixel in that section to be considered a candidate colony pixel. We will refer to each candidate colony as a blob, and each candidate colony pixel as a blob pixel. Thresholds will differ between sections with the edges between sections being sharp. Given that sections were randomly defined, such sharp edges are per definition artificial. Thus, before thresholds can be invoked, and thus before colony and background pixels can be defined, the surface of thresholds across a plate must be smoothed to remove sharp edges. We smooth the surface of thresholds using a Gaussian filter: i.e. the weights for threshold values in surrounding pixel positions in contributing to the smoothed threshold value of any one pixel-position of interest is given by a normal distribution, where the normal distribution only takes into account the pixel distance. The sigma, i.e. standard deviation, of this Gaussian distribution of weights is set to a pixel-distance of 30. After smoothing, each pixel position has its own, private threshold value for what pixel intensity is considered to reflect a blob pixel. Fig S4B shows the Otsu-thresholds for all pixels in one local area of the example plate, with the center of this local area being centerd on a blob. We invoke this threshold value to call candidate blob pixels such that each pixel is either defined as a candidate blob pixel or not. The distinction between blob (red) and non-blob pixels (blue), after invoking the Otsu-thresholds, for the example plate is shown in Fig S4C. Note that substantial noise remains at this stage (see e.g. red line of pixels falsely called as blob pixel in the bottom of the image).

- Third, the candidate blob image is de-noised in a multi-step process:

a) We erode away minute candidate blobs consisting of very few pixels (3 iterations with a kernel size 3x3). This removes isolated candidate blobs where no pixel is further than 3 pixels from the edge.

b) The erosion in (a) distorts the boundaries of true blobs. To restore these boundaries to their original shape for the remaining blobs, the output of (a) is binary propagated using the original blob detection as mask.

c) We then erode the background produced in (b), causing small and narrow pieces of background to be removed. The settings are the same as in (a). This causes the blobs to be expanded, which we counteract in (d).

d) The eroded background from (c) is binary propagated using the background as it was before the erosion in (c) as mask. This means that all small and narrow background pieces, for which no pixel in that region was further than 3 pixels from a blob, to be converted into blobs.

   Again, note that the aim in this gridding step is not to precisely define colony shapes, only to robustly register colony positions. In principle the steps above are grouped: (a) and (b) simplifies the blob detections by removing some blobs (reclassifies them as background) that do not represent colonies, while retaining the original shape of the blobs not removed. Next, (c) and (d) removes parts of the background (reclassifies them as blobs) without distorting the other background detections. The steps involved, (a) - (d) can be seen as a de-noising process. The effect of the de-noising process is subtle and consists of the removal of small background features that if retained and included in blobs could seriously distort calling of their center positions. The outcome of the de-noising is shown for the example plate in Fig S4D. Note that after the de-noising, there are still a substantial number of larger background features that are falsely called as blobs (see e.g. red line of features falsely called as blobs in the bottom of the image).

- Fourth, to remove these larger artefact background features, blobs are filtered by size and shape. Because the Otsu-thresholding only looks at opacities in pixels, without considering how these relate to their neighbors, and the de-noising step only removes very small blobs and background “islands”, the blobs can at this stage correspond to a variety of phenomenon. These include, e.g. plate edges, dust particles, scratches and

discolorations in the plastic. Such irrelevant blobs must be removed so that they don't influence the grid placement. Relevant blobs that can be trusted as representatives of actual colonies have size restrictions. Most importantly, their area must be smaller than the product of the vertical and horizontal distance between colonies: else they would have grown into their neighbors, which do not occur in experiments of reasonable length. We also apply a minimum size requirement of 40 pixels. This is heuristically set to buffer against the vast majority of dust particles, excluding hairs, while being a low enough threshold to retain the vast majority of the smallest colonies. Note that it is vastly more important to the gridding algorithm to remove all irrelevant blobs than it is to keep all the relevant ones. In the next step we apply a shape filter that assumes blobs corresponding to true colonies to be reasonably symmetrical (round) in shape. This is done in two steps:

- Scan-o-matic verifies that the extents of the blob along the horizontal and vertical axis deviate less than 25% from each other. Scratches and hairs are typical examples of non-square features that may be falsely called as blobs, unless a shape filter is employed.
- The shape of the blob is verified to be roughly circular by comparing the size of the blob (in pixels) to the ideal circle size that would have produced an equal sized bounding box. Here we allow for a 10% deviation.

We thus call true blobs, blobs that:

a. Cover >40 pixels
b. Cover fewer pixels than the product of the expected horizontal and vertical distance, in pixels, between colonies
c. Have a reasonably square bounding box
d. Have a size that corresponds to being roughly circular within their bounding box

The outcome of the blob detection and filtering is shown in Fig S4E and S4F. Fig S4E shows the outcome of the size selection. Fig S4F shows the outcome of the shape selection. Blobs failing to pass any of the thresholds are discarded. Because the aim is to align a constructed ideal grid to the observed colony positions, it is quite irrelevant to keep all blobs that in fact correspond to colonies; the colonies, and the blobs are arranged in neat rows and columns because of the robotic pinning and all that is

needed is support for all rows and colonies. Note again: falsely called blobs have a much higher chance than missing blobs at skewing the grid placement; therefore, the filtering step is very restrictive. This can be seen in Fig S4F: a true colony (blue box) is rejected by the shape selection, but as reflected in Fig S4H, this does not in any way effect the correct placement of the grid.

- Fifth, we calculate the two-dimensional mass center coordinates for each retained blob. These coordinates are stored in an array.
- Sixth, we construct the virtual ideal grid. This is done by considering the number of pins, i.e. 1536 or 384, as well as the average empirical spacing between these pins, i.e. roughly 50 and 100 pixels in 1536 and 384 formats, respectively (actual distance is a decimal value and it is not identical for horizontal and vertical distances). Thus, the idealized grid assumes that all pin centers, and consequently the center of all deposited colonies, all have equal spacing. The primary purpose of the virtual grid is to detect the best supported center of the pinning array: if the colony deposition by the pinning pad didn't exactly follow the expected spacing for all colonies, due to distortions in the production and/or handling of the plastic, it has no, or extremely little, effect on the analysis outcome.
- Seventh, the virtual grid is fitted to the stored blob array such that the sum of errors between grid intersections in the idealized grid and the center positions of the corresponding retained blobs are minimized.
- Eight, for some colony positions specified by the ideal grid there will be retained blobs nearby. For these, we replace the colony position specified by the ideal grid with the actually observed position of that blob. A blob can only be used to update one position and each position in the ideal grid can only be updated by the nearest blob. Further, the square distance between blob and ideal grid intersection cannot exceed a heuristically set threshold of 105 pixels. This is to not distort the edges of the grid with rare cases of remaining falsely retained blobs.

Overall, the gridding procedure is highly accurate and robust. It is not sensitive to how the user defined the plate during fixture calibration as long as:

a) The entire area where the colonies are is included
b) The neighboring plates' (there are four plates per image) colonies were not included.

It robustly deals with variations in experimental design. However, if experimental conditions are extremely harsh or the experimental design was very far from optimal, such that the outer rows or columns of colonies are non-existent, or very close to non-existent, problems can emerge. In these cases, the grid may sometimes become offset, one row or column, relative the actual colony array. To allow the user to address these rare instances, the gridding can be adjusted manually and the analysis rerun. The final outcome of the gridding for the example plate is shown in Fig S4G (the whole plate) and S4H (zoom in on the area with the falsely rejected blob).

**3.4.2.2 Colony detection, segmentation and pixel intensity analysis:** Once the positions for all of the colonies have been defined by the gridding procedure, we can exactly detect and quantify the colonies in their respective local contexts. The process proceeds somewhat similar to the grid placement (see 3.4.2.1).

1. We first partition the image into sections. These sections need not be random, as in 3.4.2.1, because the grid now specifies where to find colonies. Thus, the sections correspond to the local areas around each of the 384 or 1536 grid intersections (colony positions). Each local area is defined by a bounding box, centerd on each grid intersection, with sides equal to an average distance between pins (i.e. roughly 50 and 100, for 1536 and 384, respectively). From here on and until spatial normalization (see section 5), each section is analyzed independently and in isolation from its neighbors. The description of the remaining steps will therefore be for one such section, but it is executed in parallel for all section-boxes for all plates.
2. Second, the box is smoothed. Smoothing is done to prevent dust, bubbles, hair, scratches, cracks, and discolorations from confounding the assignment of blob and background pixels. Smooth is done using a 2-dimensional median filter with a size of 3x3 pixels; i.e. the pixel opacity value of each pixel is replaced by the median of itself and its eight closest neighbors. It should be noted that the smoothing is done on a copy of the image section: it is not included in the process of quantifying the colony population size, only to define where the colony is. The effects of smoothing is shown in Figure S5A and B:
    - Fig S5A shows a raw image, before the smooth.

- Fig S5B illustrates how the smoothing reduces the impact of three types of noise in the vicinity of three different colonies: a hair (upper panels), specks of dust (middle panels) and scratches (lower panels).

3. Third, we separate background and blob pixels by a slightly modified Otsu-threshold (Otsu 1975). The actual threshold is shifted half an opacity unit upwards from the calculated Otsu-threshold, thus becoming somewhat more restrictive than optimal. This has the effect of slightly under-estimating the horizontal dimensions of the colony. The purpose of the underestimation is to buffer against the risk of spuriously detecting parts of the background as colony. By this manoeuvre the potentially overlapping pixel opacity value distributions are separated into two discrete categories: blob pixels and non-blob pixels. This is the functional equivalent of step 2 in 3.4.2.1. Distributions will overlap greatly during early phases of growth when there's little difference between colony and background transparencies. When colonies grow, the distributions drift apart and become easier to separate. Fig S5C shows one random example of what the overlapping blob and background pixel distributions can look like.
4. Fourth, the pixel-area(s) detected in the previous step, the blobs, are binary dilated in two iterations by a 7x7 sized kernel with rounded corners:

   [[0, 0, 1, 1, 1, 0, 0],

   [0, 1, 1, 1, 1, 1, 0],

   [1, 1, 1, 1, 1, 1, 1],

   [1, 1, 1, 1, 1, 1, 1],

   [1, 1, 1, 1, 1, 1, 1],

   [0, 1, 1, 1, 1, 1, 0],

   [0, 0, 1, 1, 1, 0, 0]]

   This has the net effect of smoothing the blob contours. Assuming that true horizontal growth is symmetric, on the scale captured by the current pixel size, irregularities in the blob edge are artificial. Smoothing is achieved by a blob dilation, rather than blob erosion, to compensate for the slight underestimation of the blobs horizontal dimensions in the previous step. The dilation also has the added benefit of merging very proximate blobs. Particularly in early images, when the cell layer in the colony is only a few cells

thick, there is a slight tendency for the thresholding to partition true blobs into several very proximate, smaller blobs. The dilation almost completely circumvents this issue. Note that being too permissive when defining the horizontal colony size, i.e. including a few pixels of the actual background into the measure of the colony, will have next to no effect on the estimated population size due to the transformation discussed in 3.4.2.3. Omitting some true colony pixels from the blob definition would, by logic of the same transformation, cause substantially more error.

5. Fifth, at this stage, the detection may still detect more than one blob (if well separated), only one of them being true and others corresponding to artefacts. All blobs in a section are therefore ranked according to perceived similarity to a true colony. Two factors are here taken into account:
    - Which of the blobs is largest (i.e. cover the most pixels)
    - Which of the blobs is most circular (i.e. has a bounding box that is most square).

   These two quality components are weighted as follows:

   $$Q_i = A_i * min\,(E_i)/max\,(E_i)$$

   $Q_i$ is the blob quality of the $i$:th blob, $A_i$ is the area of the blob (in pixels) and $E_i$ is the extents vector of the bounding box for the blob. If the mass-center of a rejected blob falls on a blob pixel of the highest ranked blob, it is accepted as being a part of the actual colony; else it is discarded as trash. Trashed pixels correspond to specks of dust, bubbles, discolorations, scratches on the plastic, or other similar types of noise and are not considered further, i.e. they are not regarded either as colonies or background.

6. Sixth, if it is not the first image analyzed, the colony blob is compared to the detection in the latest analyzed image. Note that images are analyzed in a time-reverse manner; thus, colonies are first called on the last image, when they are largest and most easily distinguished from both background and noise. The comparison aims to detect instances where the change in colony size is clearly unrealistic, and the earlier time point colony detection therefore constitutes a likely artefact. First, colony detection could have failed completely at the current time point, i.e. no blob has been called. If so, the previous detection is used. Second, if both images do have blobs, we test their overlap by comparing the absolute number of pixels present in one but not the other (in logic, this is referred to as material nonimplication) blob to the square root of the area of the previous

blob: if this ratio is larger than eight, the current detection is considered to be a potential artefact. The threshold, 8, is heuristically set to reflect a substantial lack of correspondence between the two sequential detections. A large difference in colony area can have two origins.

- It can be the result of a partially failed definition of colony area
- The colony position can have been shifted in the image due to disturbance of the scanner (malfunction in the positioning of the lamp and/or sensor, human factor, etc.). If this latter scenario is the case, a second iteration of the test is performed but first the mass centers of the compared blobs are aligned.

As with a missing blob, previous detection is used if both tests fail. Two things should be noted:

a. This implies that a previous detection can be maintained for several images in a row
b. Failed detections cause a permissive inclusion of pixels into what is considered the colony.

However, with regards to (b) due to the transformation in 3.4.2.3, it will have a marginal noise effect.

7. Seventh, after the pixels corresponding to the colony have been called, we decide what pixels that accurately represent the local background. We define the local background as pixels in the image section that belongs to neither the colony nor the trash. However, a colony at its edge is often only a single cell layer thick, and has marginal effect on light transmission; it is therefore extremely hard to distinguish the outermost cells from the surrounding background. Furthermore, pixels are substantially bigger than a single cell, and the true colony edge will therefore run through pixels, rather than perfectly follow pixel boundaries. As a consequence of both these factors, the assignment of pixels to colonies will never be perfect. The colony detection and definition largely accounts for this problem by its dilation step. However, the background definition does not. Thus, to ensure that absolutely no part of the colony is ever included in the evaluation of its surrounding background an extra safety margin, around each defined colony, is applied. The safety margin is established by three iterations of binary erosion, with a 3x3 kernel. If the background fills out to the edge of the image section, erosion is not applied. The erosion removes the three outermost tiers of pixel-positions along the edge of the

background/colony boundary. The pixels of three colonies, and their underlying data, are shown in Fig S5B.

8. Eight, we calculate the contribution to the pixel opacity values of the colony made by the nutrient medium and plastic by estimating the average opacity value for the background in the section. We thus use a single local background value, for each colony. It is conceivable that the background from the plastic in the plate and the nutrient medium could be estimated pixel-wise, and subtracted as a complex background, if plates were scanned before deposition of colonies. However, in logistical terms, this would be quite challenging when performed in a high throughput context and risks introducing substantial error. Moreover, each colony covers an area that is much smaller than the local background, meaning that a single value estimate based on the average background in the area is likely to be a good approximation also for the pixels covered by the colony. The pixel opacity values of the local background follow a distribution. The extremes of this distribution may vary dramatically and represent artefacts (i.e. dust, discolorations, scratches, hair, bubbles, and tears, and neighboring colonies expanding into the area towards the end of experiments. Correcting all such pixels with 100% accuracy in the image smoothing step is not realistic. We address this problem as for the grayscale detection (see 3.4.1.3.4): by forming a mean of the mid 50% of the pixel opacity values defined as belonging to the background. We subtract this estimate of the local background pixel opacity values from the pixel opacity values of each colony pixel. These background subtracted colony pixel values represent the pixel opacity effect of the cells within each pixel. Because negative values have no realistic physical interpretation and will be problematic for the downstream analysis, they are set to 0. The background subtracted pixel opacity values in each section are retained for downstream use. The effect of background subtraction is shown in Fig 1B.

**3.4.2.3 Converting background subtracted colony pixel opacity values to cell counts per pixel and colony population size:** The background subtracted pixel opacity values have no meaningful unit and cannot be directly interpreted as population size. For them to provide exact biological information, they must first be converted into actual cell counts: i.e. we must answer the question of how many cells a certain recorded background subtracted pixel opacity value corresponds to. The density of cells in a particular volume does not scale linearly with the amount of light that passes through that volume because the cells scatter light: the more

cells that are added to the volume, the less light each additional cell scatters. In essence: cells shadow each other. This property of light, and of other types of radiation, means that cell number, or cell density, does not scale linearly with optical density (opacity). This is a serious and widely recognized problem that, unless attended to, dramatically confounds growth estimates (Blomberg 2011). When manually measuring optical density, this is handled by disregarding the error, when the error is small (e.g. at optical densities below 0.5) and diluting the cells when the error is substantial (e.g. at optical densities above 0.5). We aspire to both high throughput and high precision and neither disregard nor dilutions are therefore viable options. Instead, we established a transformation function that transforms the recorded optical signals to the true quantification of cell count, as previously done (Warringer and Blomberg 2003). The standard procedure for establishing such transformation functions is to consider a smaller set of samples (cell populations) that represent the full range of population sizes that can reliably be recorded. The actual number of cells in these samples is then determined, either by separating cells and counting them one by one, or by diluting each sample and measuring the optical density of diluted samples. In the latter case, obtained values are multiplied by the dilution factor. To convert our background subtracted pixel opacity values into actual cell counts per pixel, we performed such a calibration experiment and established the corresponding transformation function. This was performed as follows: We considered 42 colonies of the yeast strain BY4743 covering the complete range of colony population sizes that reasonably can be reached in Scan-o-matic experiments. After first imaging the colonies, the solid media (agar) was cut into pieces with a scalpel, each with a colony on top. Carefully, the agar sections were removed from the plate, taking care not to touch the colonies. Colonies were then washed off from the agar into sterile water. This was done by extensive vortexing. We then counted the actual number of washed of cells by splitting the sample obtained from each colony in two completely independent methods:

1. First, OD600 in a spectrometer (Pharmacia Biotech NovaspecII) of dilutions (OD of diluted samples = 0.1 to 0.2) was measured, the background subtracted and recorded values were multiplied by the dilution factor. These optical densities were transformed into cell counts based on the empirically well supported estimate that 1 mL of OD = 1.00 of BY4743 cells corresponds to $1x10^7$ such cells.

2. Second, cells were extensively sonicated such that no cells were sticking together and cells in a sub-fraction of the sample were counted using a Fluorescence Activated Sorting Machine (FACS) (BD FACSAria, Germany). The number of cells in the entire sample was calculated by multiplication in relation to the fraction analyzed.

The two cell count measures (OD and FACS) showed close to perfect linear correlation (Fig S6A). Given that the two methods are independent and suffer from different types of noise and bias, this is strong evidence that the recorded measures well capture actual cell numbers in the analyzed colonies. Given that the linear correlation is near perfect, it is irrelevant which of the two measures is used for actual transformation of the background subtracted pixel opacity values: the outcome is identical for all practical purposes. The transformation aims to establish a polynomial function whereby background-subtracted pixel opacity values can be converted into actual cell counts per pixel. The polynomial function was established using the 42 colonies. Note that whereas the calibration measurements (FACS, OD) are total cells per colony, not cells per pixel, the Scan-o-matic measures are a pixel-based distribution of background-subtracted pixel opacity values for that colony. The distributions of background subtracted pixel opacity values for the 42 colonies are shown in Fig S6B. We must thus relate measurements of total cells per colony to the Scan-o-matic estimated distributions of background-subtracted pixel opacity values in each of these colonies. We resolve this challenge as follows:

- Scan-o-matic estimates of cell counts for a colony, $Y_{est}$, can be expressed as a function, *f*, of the frequency distribution, $\varphi$, of background subtracted pixel opacity values for the colony. In essence: $Y_{est} = f(\varphi)$.

- We consider a polynomial function to capture the properties of $f(\varphi)$. This can be expressed as: $Y_{est} = \sum_{i=0}^{n-1}(ax_i^5 + bx_i)y_i$, where *a* and *b* are constants. The index *i* is the *i*:th unique observed background-subtracted pixel opacity value for the colony considered, from the first observed value, index (0), to the final index *n-1* (*n* the number of indices), $x_i$is the *i:*th background subtracted pixel opacity value and $y_i$ is the number of pixels with that value in the colony.

- The error, $E_{j,est}$, in the Scan-o-matic estimate of cell number for the colony, $j$ compared to the known actual value, can be expressed as: $E_{j,est} = Y_{j,obs} - Y_{j,est}$. $Y_{j,obs}$ is the OD observation of cell counts for colony $j$, $Y_{j,est}$ is the estimate for colony $j$. The sum of squared errors, $SSE$, over the total number of colonies $m$, becomes: $SSE = \sum_{j=0}^{m-1}\left(Y_{j,obs} - Y_{j,est}\right)^2$.

- The least-square optimization to find the polynomial that consequently produces the smallest error is done on the constants $a$ and $b$ such that $SSE$ becomes minimal using the entire set of measurements jointly from the 42 colonies. Lower degree polynomials are incapable of capturing the sharp rise in cell count at higher pixel opacity values, i.e. of capturing the high cell shadowing effect at higher cell densities. A higher degree polynomial adds more flexibility, but is not supported by the data observed. A polynomial that includes the intermediate exponents over-fits the data, such that it has negative slopes for part of the curve (implying fewer cells with higher image opacity). This has no realistic physical interpretation. The $x^0$ coefficient is omitted because the in-data are background-subtracted opacity values: thus, a zero value should imply no cells present.

- The best fit was found for $a$=1.2678179849224454*$10^{-4}$ and $b$=128.91562154694719. The polynomial is shown in Fig S6C. The calibration function to convert background-subtracted pixel opacity values to actual cell counts was thus defined to be: $y$ = 1.2678179849224454*$10^{-4}x^5$ + 128.91562154694719$x$, where $y$ is the cell count and $x$ is background-subtracted pixel opacity values.

To further validate that the calibration works and that there is sufficient and accurate information in the images over the full range of colony population sizes (more than tree orders of magnitude size differences), the estimated population sizes, $y_{est}$, were plotted against the observed sizes, $y_{obs}$. This comparison is shown in Fig S6D. As can be seen, there is a near perfect 1:1 correlation across the full range of colony population sizes (slope $k$ = 0.98; Pearson, $r^2$ = 0.98). The result therefore shows that a polynomial can accurately translate image data to population size, independent of colony size. It also shows that for all of the 42 colonies studied, the image data is a correct and sufficient representation of the colony for the cell numbers to be correctly calculated. As a final step

in the image analysis, we therefore transform all background-subtracted pixel opacity values, in all called colonies, into cell counts, using the established calibration function. Cell count estimates per colony and time are stored as colony population size in NumPy files and XML-format. Note the following:

1. The preceding calibration of pixel intensities to those recorded for a grey-scale calibration target with fixed standardized pixel intensities accounts for any reasonable variations in instrument performance (including lamp status) and environmental properties (light, heat, humidity, other radiation). We therefore expect the transformation from background-subtracted pixel opacity values to cell counts to be very robust to such variations. Repeating the cell count calibration experiment for each individual scanner and time of experimentation is therefore not necessary.

2. Because of the preceding subtraction of the local background around colonies, the transformation function is very robust to variations in medium thickness, evenness, and Coloration. Repeating the cell count calibration experiment for each type of medium is therefore not required (although see point 6 below)

3. Based on our experience with calibration functions in liquid cultivations (Warringer and Blomberg 2003), we estimate the calibration function to be robust to within-species variations in cell size, shape and composition. Repeating the cell count calibration experiment for each genotype is therefore not required (although see point 5 below). For a species whose ploidy, cell size or cell shape differ dramatically from *S. cerevisiae,* the calibration function will only be approximately correct. As a general rule, we recommend the user to perform individual cell count calibration experiments for yeasts outside the *Saccharomyces sensu stricto* clade and for bacteria

4. Though the output of the cell calibration function gives values as cell counts per pixel, it is optimized to produce reliable cell counts for entire colonies. Because of this and the fact that colonies with the same number of cells tend to share morphology (number of pixels with certain background subtracted opacity values), the per pixel cell count is not as accurate as the sum of per pixel cell counts over the entire colony. Due to this, if colonies with drastically different morphologies are investigated, we recommend that cell calibration functions are determined for the specific colony morphology of

interest. Separate cell count calibration experiments must also be performed when measuring the growth of yeast accumulating strongly colored pigments or metal species, such as tellurium (Ottosson et al. 2010), over time.

5. With extremely large background subtracted pixel opacity values, transformation to cell count space is not equally reliable. This is because the transformation function becomes very steep in this region such that even a small error in estimation of background-subtracted pixel opacity values can lead to large errors in cell counts. When this happens, if a pixel is estimated to have more than 4200 cells (corresponding to the most extreme background-subtracted pixel opacity value used in the cell calibration function), a warning is emitted. 4200 is much above what you typically observe in 384 or 1536 format colonies: even in the densest colonies, pixels will only very rarely reach this value (e.g. if pixels are affected by very dark dust particles). The warning signal indicates in which scans a certain colony has any pixel surpassing this value. This allows the operator to visually inspect the images of the experiment in question to ensure that the data is indeed sound. If cells have an intense dark coloration, the threshold will regularly be reached and the warning displayed. This may happen with normal yeast cells, if intracellular accumulation of inorganic (e.g. many metals, such as tellurium (Ottosson et al. 2010), or organic (e.g. certain metabolites) molecules occurs. We strongly caution against scanning on media inducing dark coloration. If such experiments are performed, independent calibration experiments must be done to obtain alternative polynomial fit coefficients. In our standard analyses, we use synthetic complete medium with a very low-degree of opacity.

6. The cell calibration function is not valid for pixels with background-subtracted pixel opacity values below zero. This happens, albeit rarely, when a colony pixel has a lower pixel opacity value than the background estimate. This, as discussed above, is corrected before applying the calibration function by setting all negative values to zero.

**4. Extracting growth variables from dense growth curves**

**4.1 Smoothing of growth curves**: Cell count estimates for a colony are finally re-arranged to a per colony time series, rather than a per scan estimate. Each time-series of cell counts constitute a growth curve that depicts that colony's change in population size over time. Given that estimates are taken with 20 minute intervals, each growth curve will be represented with a very large number of measures (217 for a typical 3 day experiment), i.e. it will be very highly resolved. The high resolution means that there is no need to fit the curve to a model that makes assumptions on what growth should look like. This is a considerable advantage relative previous solid-medium based methods. However, there will unavoidably be some noise in the growth curve, i.e. there will be errors in the estimates of cell counts over time. Such errors must be minimized before variables, such as growth rates, can be extracted from the curves: otherwise, parameter estimates may be artefacts that reflect noise rather than biology. Any removal of noise must take care not to remove any true biological features of the curve. We address this challenge by smoothing the growth curve in two steps.

1. The first step is designed to take care of sudden spikes in the growth curve. Such spikes may be due to vibrations, variations in the power flow, dust particles passing by the sensor, or due to fluctuations in properties of the lamp, in particular when it approaches the end of its life span. There is no biological reason for spikes to occur and they must be removed in order not to distort growth estimates. The upstream analysis has several features, such as the conversion to pixel opacity values and the image registration with orientation markers, which remove the vast majority of the events that would have caused spikes. The first curve smoothing filter is included as a final safe-guard. We filter for spikes using a median filter of size = 5, i.e., the median of each of five consecutive cell count values replaces the third value in each such series of five. The filter's logic implies that if the curve is continuously increasing (i.e. being monotonic), as is the typical growth curve scenario, the data will not be altered in any way by the filter. If a measurement stands out in either negative or positive direction it will be replaced by the median of its neighbors. We smooth initial and final measures by employing reflect edge conditions: e.g. when smoothing the first estimate we assume that the two fictive measurements preceding it would have been equal to the first and second value. The removal of both positive and negative spikes by median filtering is shown for one example growth curve in Fig S7A.

2. Second, we employ a Gaussian filter of width $\sigma$ = 1.5. This allows estimates for all time points surrounding a particular time point to contribute to adjusting its estimate, with the degree of each contribution, i.e. its weight, being defined by the distance (in time). Weights follow a normal distribution centerd on the estimate itself, with the standard deviation of this weight distribution set to 1.5. The Gaussian filter efficiently reduces lower amplitude noise, even if this noise occurs with a relatively high frequency. This is shown for two very noisy growth curves in Fig S7B. The high frequency noise in the cases shown is shared across all colonies in one column and derives from the belt of the scanner approaching the end of its life-span, i.e. from a loss of accuracy in the positioning of the lamp at this specific column position.

These two smoothing procedures are standard in signal processing and very soft, i.e. we are very conservative when removing noise, preferring not to risk smoothing out true biological features, such as multiphasic growth. The chosen settings of the two smoothing procedures, 5 and 1.5 respectively, may not be optimal for all growth curves. In particular, with different time intervals between measurements, other settings may be preferable. However, based on visual inspection of the consequences of smoothing over many thousands of growth curves, these are the settings that we recommend for yeast experiments with 20 minute intervals between measurements. Other settings can be applied, using the scanomatic.dataProcessing.Phenotyper object in python. However, currently no user interface exists. In any case, we caution users against shifting these thresholds far away from these settings. We also strongly emphasize that settings must be maintained constant throughout a complete experimental series for fair comparisons between experiments to be done. Because both the median and the Gaussian filter are local and very light, they cannot fully account for complex noise that persists over many consecutive time points. This can be seen for the very noisy growth curves in Fig S7B: arrows point towards some cases of complex noise that cannot be completely accounted for by the Gaussian filter. Note that highly distorted growth curves, with dramatic noise that derives from a fundamentally incorrect handling of software or hardware, cannot at all be compensated for by smoothing. One source of such dramatic noise is an incorrect placement of the calibration strip in its slot on the fixture. This is shown in Fig S7C. In such cases, Scan-o-matic allows the operator to patch an image of the calibration strip from a separately scanned fixture to the image files of the affected experiment, allowing correct calibration of the data which is unperturbed.

**4.2 Extracting phenotypes and auxiliary growth measures:** From the smoothed population size growth curves we extract biological properties, i.e. growth phenotypes (or fitness components), as well as some auxiliary measures that serve as quality indicators of each individual growth curve. In the present work, we only extract the maximum growth rate, or rather the minimum population doubling time, as a growth phenotype. While doing this, we recognize that it does not completely capture the complex dynamics of growth and that much more information is contained in the curves than represented by this single phenotype. When extracting the maximum growth rate, we employ the following assumption. We assume the budding yeast life-cycle on solid medium to be completely mitotic (i.e. no meiosis and no hyphae formation is assumed to occur), the length of the mitotic cell cycle to be normally distributed and cell death to be negligible. Furthermore, we assume no population structure. In these simplified circumstances, a population is expected to double in size during the time of an average cell cycle. Consequently, a growth-curve with a $\log_2$ *y*-axis should be linear as long as environmental conditions are constant (from the perspective of the cells) and each unit on the *y*-axis represents one doubling. In reality, both the genetic and the non-genetic environment of each cell are in constant flux and assumptions of an extended phase of such linear growth on a $\log_2$ scale are precarious. Growth is rarely $\log_2$ linear for any extended period of time. This becomes increasingly evident when growth, as here, is precisely estimated at short intervals (Fig S8A; the first derivatives of three random growth curves from genetically identical BY4743 growing on the same basal medium are shown). This argues for using dense growth estimates and extracting the maximum growth rate over short time periods, rather than fit growth models that assume extended log-linear growth phases, to the data. The second grave complication for fitting growth models to sparse data, or even fitting growth models to densely sampled data like ours, is that a considerable number of the curves do not follow the simple one modality growth-pattern. The probably most common variation to the norm is multiphasic growth. This is exemplified in Fig S8B, showing a curve with three exponential growth phases. We address these issues by minimizing the assumptions about the growth curve and extract the fastest population doubling time. To reliably measure the maximum slope of the smoothed growth curve we perform all possible linear regressions with a window size of five. Note that five is selected to be suitable with the interval used here (20 minutes) and coincides with the approximate duration of the generation time for a fast

growing culture (Fig S8A; red arrows indicate the five time-point window for maximum growth rate). We caution users against comparisons across experiments that have been measured with different time intervals. From these we select the linear regression with the steepest slope and calculate the minimal population doubling time (note: used interchangeably with maximum growth rate throughout the text). We also extract four auxiliary measures:

- initial colony population size
- time of the minimum population size doubling time
- error of the linear regression that underlies extraction of the minimal doubling time
- growth curve fit to an initial value extended version of the classical Chapman-Richard model are extracted from smoothed growth curves.

The latter three are used to assist the user during curve quality control. We emphasize that no model is fitted to the data before extraction of the maximum growth rate. The Chapman-Richard model is never used to draw direct biological conclusions, just to flag potentially low-quality curves for visual inspection. Model fitting is not needed with the curve resolution here achieved and because they could lead to the wrong conclusions (for the above reasons), and we caution users against it.

**4.3 Initial population size:** To obtain a robust measure of the number of initially deposited cells, we calculate the mean of the initial three (from the first three time-points) population sizes. The initial population size is not used for quality control by Scan-o-matic. Here, it is only extracted and analyzed as a potential confounding variable. As shown (Fig 3B, left panel), the initial population size is positively correlated to maximum growth rate. Normalization to internal spatially distributed standards (see 5.2.) removes this correlation (Fig 3B, right panel).

**4.4 Time of minimum population size doubling time:** The central of the five time points associated to the steepest linear regression is stored as the auxiliary quality measure time of maximum growth rate (minimum population doubling time). This is used for quality control, as indicated in 5.1. A strongly deviating (compared to the other colonies in that experiment) time of maximum growth rate sometimes indicate that a rare noise effect, rather than biology, drives the reported population doubling time phenotype.

**4.5 Error of the linear regression underlying extraction of the maximal doubling time:** The five *x*, *y* value pairs used as a basis for extracting the steepest linear regression spread around the calculated linear regression. The larger the spread, the noisier the curve is at its steepest part and the less precise the population size doubling time estimate. If the curve is noisy, it also, as with 4.4, increases the risk that the noise is driving the reported phenotype. We therefore report the standard error of the estimate of the steepest linear regression and store it as quality control measure, as indicated in 5.1.

**4.6 Curve fit to an extended Chapman-Richards Four Parameter Curve:**
The poor fit of colony population size estimates to an ideal model of a growth curve either reflects that the model poorly reflects the biological reality, i.e. correspond to non-standard growth, or that the growth data underlying the curve is noisy, i.e. affected by random errors. Thus, the model fit can be useful in directing attention to curves that may warrant particular scrutiny, either for exclusion (noisy curves) or follow-up analysis (non-standard growth). We therefore used an extended version (allowing for a differentially long exponential growth phase) of the classical Chapman-Richards four parameter growth curve model (Pylvänäinen 2005) as one of several quality indices that call the operator's attention. We fitted this model to each series of population size estimates, and used the degree of fit as a quality indicator, as indicated in 5.1. The model is not used for extracting the generation times themselves, only to indicate growth curves which are of potentially lower quality and for which generation times should be considered with caution. The extension corresponds to inclusion of an initial y-offset, *D*, which makes the model better fit to our data as the curves at $t = 0$ tend to start around $2^{15}$ cells rather than at the classical Chapman Richards assumption of $y = 0$. The model fit is done in $y$-value space. To ascertain that parameters are kept within their defined ranges, the input parameters to the model are transformed as follows:

$$b_0 = e^{b_0}$$

$$b_2 = e^{b_2}$$

$$b_3 = \frac{e^{b_3}}{e^{b_3} + 1}$$

$$b_1 = \frac{e^{b_1}}{e^{b_1} + 1} * b_3 + (1 - b_3)$$

Initial parameter estimation was optimized iteratively with parameter estimates for the first iteration selected being based on previously suggested estimates (Pylvänäinen 2005). In

consecutive iterations, the mean parameter values from the previous iteration, for over 6,000 curves, were used as new parameter estimates. Values producing the least summed error of fit and used by Scan-o-matic were: $b_0 = 1.64$, $b_1 = -0.1$, $b_2 = -2.46$, $b_3 = 0.1$, $D = 15.18$. The model fit to each growth curve was recorded and used as a quality index of that curve, i.e. a poor fit was taken to indicate a growth curve that is suspicious and in need of further attention. Again: observe that no model fitting is performed as part of extracting of the maximum growth rate. This is not needed with the here achieved time-resolution. Indeed, it would risk strongly distorting maximum growth rate extractions from the many growth curves that do not resemble curves in the standard growth model (Fig S8B).

**5. Quality control and spatial normalization of colony growth data**

**5.1 Quality control:** The sole output phenotype from the analysis is the minimum doubling time/generation time. Because growth, as discussed in section 4, is a complex process that results in a plethora of types of growth curves, simple thresholds and naïve models are not reliable enough to cast judgement of whether a curve is a good representation of the biological phenomena studied or not. Automated evaluation of curves could potentially reach the same fidelity as the expert human, but it lies outside the scope of this study. It is therefore critical that the assistance that the quality indices provide doesn't bias the human observer in their judgement. We combine the need for effective but unbiased quality assessment by presenting the curves sorted by the quality measures (see 4.4 to 4.6), without indicating if the measure should be considered “good” or “bad”. A large error of the linear regression, a poor model fit and a very late or early extraction of minimum population size doubling time may suggest that a growth curve~~s~~ is of low quality. Scan-o-matic delegates decisions on whether to reject or retain suspicious curves to the operator, indicating positions with curves of suspected low quality on a heatmap, on the basis of the quality indicator of choice. Low quality growth curves should be rejected. Rejection must occur before normalization, not to negatively influence the normalization procedure. We urge users to be conservative in retaining growth curves, in particular if replication is high, which it always should be. In the reported experiments ~0.3% of curves were excluded. During quality control, meta-data for the experiments can be joined with the observed phenotypes by importing a meta-data containing spreadsheet file. This establishes a connection between the growth curve inspected and a description of the colony. The spreadsheet file needs to meet these requirements:

1. Each row on a sheet represents all information about a position on the plate.
2. Each row on a sheet only represents data for one position on the plate.
3. Order of rows in a sheet should correspond to first left to right order of colonies in a plate row and then a top to bottom order of plate rows (assuming a landscape orientation of each plate
4. Each sheet represents a complete plate, a fourth of a plate or a collection of several full plates.

Both OpenOffice/LibreOffice and Microsoft Excel file formats are supported. Examples are provided here: https://github.com/local-minimum/scanomatic/wiki/Strain-meta-data-importable-formats.

## 5.2 Normalization

### 5.2.1 Origin of bias and need for normalization

A fundamental challenge is accounting for systematic errors that create unacceptably high rates of false positives and negatives, even when measurement precision is excellent. Technical, biological and other biases can come from many different sources, such as variation in temperature, radiation, light influx, humidity, dust, scanner light source or sensor, scanner mechanics, robot mechanics, software updates, power fluxes, composition of nutrients, chemicals and water, calibration strips, orientation markers, fixtures, plates and pin pads, contamination and mutations. Some of these sources of bias can be completely or partially accounted for by careful standardization in the lab, as described in section 1.1. Standardization ensures that experiments are performed under as identical conditions and using as identical practices, as possible. Thus, it removes, or reduces the influence, of bias. Unfortunately some sources of bias are difficult to reduce with standardization. For example:

- Random *de novo* mutations emerge when cells replicate in pre-cultures and experimental cultures
- The light intensity of scanner lamps declines over time and thereby the amount of radiation cells are exposed to
- Plates in an instrument are positioned at different physical distances from the lamp parking position.

In a wider perspective: no two experiments can be run in the same instrument, at the exact same position, at the same time and without any variation in initial conditions. Perfect

standardization and removal of all bias is therefore impossible also from a theoretical perspective. The degree to which each remaining source of bias impacts on growth estimates depends on genotype and environmental composition. Bias is therefore challenging to describe and understand. From evaluating many millions of growth curves, it is absolutely clear that the most pronounced bias that remains in Scan-o-matic after the extensive experimental standardization outlined in section 1.1 is spatially correlated variation between experimental positions within a single plate. This spatial bias can and do come from several sources:

- Nutrient medium poured into a plate settles with slight variations in media thickness across the plate, solidification of the medium at different rates in different positions leads to slight variation in agar composition, water loss occurs at different rates at different positions and the medium creeps-up along the plate walls, with slight variations. Therefore, even with extreme precautions taken, the medium properties will not be perfectly even across the plate. Variation in medium thickness means that different colonies have different access to nutrients. It also means that the medium surface will not be perfectly level. Both contribute to spatial variation in the amount of cells that are extracted by the pins from different pre-cultures and the fraction of extracted cells that are deposited at different positions on the experimental plates.
- The pinning pads used by the pinning robot are highly precise. However, there are slight irregularities and variations in the plastic. These irregularities mean that some pins collect and deposit slightly more cells than others, or collect cells from a more peripheral position in pre-cultured colonies, leading to the transfer of cells that are in a slightly different physiological state.
- The distance to the edge of plate varies between experimental positions, with colonies at the edges having fewer competitors and therefore inherently greater access to nutrients and less exposure to toxic products produced by neighboring colonies.
- Similarly, genotypes grow at different rates and extract nutrients from, and secrete toxic products to, the local environment at different rates. On a plate with many different genotypes (e.g. large mutant collections), both nutrient access and exposure to toxins will therefore vary between experimental positions depending not only on how these genotypes were initially arranged but also on how they emerge through mutations (which will disproportionally emerge in larger growing edge colonies).

- Due to the edge effect, colonies tend to be substantially larger at the end of growth towards the edges of pre-cultured plates. This means that when cells are collected from these pre-cultures, pins might collect cells from the interior of the large colonies at the plate edges, but close to the periphery of smaller colonies in the plate centers. Because cells in the center of colonies have less access to nutrients than cells in the periphery during later stages of growth, their physiologic states are different.

All these sources of spatial bias combine to create often very substantial spatial differences in the number and state of cells deposited on experimental plates and in the environment they experience when they grow. Randomization of a large number of replicates across experimental positions could convert some of the spatial bias into random error, thereby avoiding false positives and negatives in the final statistical analysis. However, the massive logistical challenges involved in producing completely random designs in high throughput screens mean that this will rarely be feasible. We encourage users to randomize replicates, or distribute replicates evenly across pre-culture and experimental plates, to the greatest extent that is practically possible, while keeping in mind that unless perfect randomization of replicates is achieved, some spatial bias will always remain.

#### 5.2.2. Normalization to account for spatial bias

Given the substantial correlation between initial population size and minimum population doubling time (Fig 3B), we first investigated a normalization approach based on initial population size. The procedure and outcome for one example plate is shown in Fig S10: more generally, the outcome is shown and commented on in Fig 3A. Initial population size, which is measured in cell number, is qualitatively different from population doubling times, which is measured in hours. We therefore performed the initial population size normalization on log scale, by least square fitting of the initial population sizes (Fig S10C) to the $\log_2$ of population doubling times (Fig S10A). This resulted in a normalization surface, capturing the spatial bias on population doubling time assuming this bias to be completely explained by variations in initial population size (Fig S10D). Finally, normalized population doubling times (Fig S10E) were obtained by subtracting the normalization surface from the observed $\log_2$ population doubling times. However, given the limited success (Fig 3A, Fig S10E) of this initial population size based approach, we rejected it in favour of a reference grid based normalization approach based on the growth rate of the internal control colonies.

In general, normalization estimates the contribution of bias to each recorded measure, and subtracts the bias to provide a more accurate, normalized estimate. Various approaches to normalization in order to account for spatial bias within plates have been employed in microbial phenomics. These include comparing experiments row-wise and column-wise, assuming that systematic differences in the mean estimates for columns or rows correspond to technical bias (Baryshnikova et al. 2010). Such normalization can only, by virtue of its use of row/column means, compensate for long-ranging smooth transitions in the bias. However, the observed spatial bias for population doubling is much more complex than that (Fig S11; left panels) and the means of columns and rows will often differ for other reasons than spatial bias, i.e. due to the genotypes they contain, so that the null-hypothesis of now growth difference between columns /rows would not be valid. Normalizing experiments by means of their own estimates is also conceptually problematic and entails a high risk of introducing rather than removing errors. To circumvent these caveats, we designed and implemented an alternative reference grid based approach that is agnostic with regards to the nature of the bias: *i.e.* the operator need not know or make any assumptions about the nature or source of the bias: it is enough to be able to estimate its magnitude over the plate. The approach is based on the following principles:

- We sacrifice a quarter of the experimental positions and introduced a reference grid of 384 evenly spaced controls that are genetically identical (i.e. the null-hypothesis is that they shall have the same growth properties). The arrangement is illustrated in Fig S12. Systematic variations between the growth estimates recorded at these control positions will therefore reflect the spatial bias acting on the experiments over the plate.
- Assuming that the spatial bias affects experimental colonies similarly (i.e. assuming no interaction between genotype and the source of bias), we can use the systematic variations between control estimates to quantify and subtract the spatial bias. Using the population doubling time recorded for the evenly spaced 384 controls, we estimate what population doubling times that would have been recorded at each of the other 1152 position - if that position had contained a control. Note that for the method to perform optimally, systematic variation between experiments/plates must be kept reasonably low: it is therefore not a replacement for, but should always be accompanied by, careful experimental standardization.

- We then subtract the estimated control $\log_2$ values for each of the 1152 positions from its corresponding actual experimental $\log_2$ measurements to obtain a normalized experimental phenotype value that, ideally, is free from spatial bias.

In detail, the control values for each of the 1152 experimental positions are obtained in a three-step procedure, as follows:

1. First, controls with extreme values that may not represent actual spatial bias, but other types of rare errors (local medium deformations, discolorations, dust, missing colonies or contaminations) must be removed. Failure to remove control estimates affected by large errors other than spatial bias may result in a normalization process that introduces rather than removes spatial bias. Fig S13 shows the outcome of normalization across a plate, with (lower panel) and without (upper panel) removal of control extremes. We define controls with extreme values using a 2D Laplacian kernel, i.e. we look at the rate of change (acceleration, second derivative) between neighboring controls on each plate. All accelerations are ranked based on how much they differ from the mean of all accelerations between controls on that plate. If there's a control position that differ more than a corrected two standard deviations (2σ, based on the acceleration values) away from the mean it is considered an outlier. We iterate the removal process, i.e. we calculate new accelerations, rank these, recalculating the mean and standard deviation and estimate outliers again for a maximum of 10 iterations - or until no more outliers are found (whichever occurs first). In each new iteration, we raise the threshold for removing controls slightly (by a factor that corresponds to the exponential of the fraction of controls remaining from the previous iteration). This compensates for that more accelerations tend to fall within 2σ when extremes are removed. On average, we discard ten of the 384 controls on each plate by this process. Note that the initial threshold (2σ) is set to be conservative with what controls we retain, i.e. we typically discard some useful control estimates. This is intentional: the consequences of imperfect normalization is much less with discarding some valid controls that correctly estimates spatial bias than in retaining even a single control whose value is dramatically influenced by an error other than spatial bias. This is shown in Fig S13.
2. Second, using all remaining control positions, the normalization surface is extended to cover all other positions (non-control and removed control positions). This is done

by standard interpolation, specifically by using the method griddata of SciPy's interpolate module. The method is a standard cubic splining, i.e. the values of all the immediate neighbors of an experimental position, vertically and horizontally, contribute equally to the estimation of its value. Note that when the value of a non-control position has been estimated, it in turn is used for estimating the values of its neighboring unfilled positions. This is particularly important towards edges: because of how the pinning is built-up: one of the outer rows and one of the outer columns completely lack controls. Here nearest set values are used. The procedure provides an estimate of the expected population doubling time in all positions, assuming that they had been controls. Note that the interpolation implies short range smoothing for the experiment positions.

3. When the surface has been completed, the $\log_2$ difference between the actually observed population size doubling time for a position, and the value of the normalization surface in that position is calculated. This $\log_2$ relative population size doubling time estimates the performance of a colony in relation to what a control would have had if it had been in that position. The values obtained will thus resemble the logarithmic strain coefficients we earlier introduced for normalization of liquid cultures (Warringer and Blomberg 2003). This $\log_2$ relative population size doubling time is reported as the final output.

The effect of normalization is reported in Fig 3C and Fig S11. The latter shows the distribution of minimum population doubling times across five plates, before (left panels) and after (right panels) normalization.

**6. Data Publication**

Both final and raw phenotypes can be exported by the user in a tab separated format. Phenotype values are appended after the user given meta-data for each colony. If no meta-data is given during quality control, phenotypes are preceded only by the positional information of the experiment. If meta-data is given it is inserted in-between the positional information and the phenotype data. Raw growth curves are stored as NumPy files, one file per scanned image. Each file is organized according to plate, row, and then column. Scan-o-matic includes a tool for easy conversion into growth-curves. The raw growth data is also stored in an XML-

format (either as full tag names or shortened aliases). Specifications for the XML are available in the Scan-o-matic repository documentation.

**7. Access to Scan-o-matic**

Scan-o-matic is currently available as two separate packages. 1) Scan-o-matic for scanning and analysis. 2) Scan-o-matic for quality control and normalization. Both are hosted at GitHub, which also contains the developmental code: https://github.com/local-minimum/scan-o-matic. There is a wiki and a user's manual included. These have detailed information about installation of the two mentioned versions as well as of many other details about the usage of Scan-o-matic. Through GitHub bugs can be reported and features requested. A set of experimental data for evaluation of the analysis procedures can also be accessed through the wiki: https://github.com/local-minimum/scanomatic/wiki/Example-experimental-data

**8. Wet-lab experimental procedure**

Solid media was cast in Plus plates (Singer Ltd, UK) designed for use with a Singer RoToR HDA robot (Singer Ltd, UK). Each plate was cast on top of the same perfectly level surface with precisely 50mL of Synthetic Complete (SC) at 50ºC. Medium was poured in the same lower left corner on all plates. Plates were left to dry for 1h at room temperature without lids and plates then remained at room temperature (with lids on) and used the next day: long enough to remove any moisture inside plate, but not long enough for any contraction of the medium to be visually detectable during the ensuing 3 day experiment. All plate preparations were performed in the same environmentally controlled space (room temperature; 23ºC). The medium composition was as follows: 0.14% Yeast Nitrogen Base (YNB, CYN2210, ForMedium), 0.50% ammonium sulphate, 0.077% Complete Supplement Mixture (CSM, DCS0019, ForMedium), 2.0% (w/v) glucose and pH buffered to 5.8 with 1.0% (w/v) succinic acid and 0.6% (w/v) NaOH. Media was supplemented with 20g/L of agar. Where indicated, 2.0% glucose was replaced by 2.0% galactose and/or supplemented with salt to 0.85M NaCl. The addition of 0.85M NaCl brings the final $Na^+$ concentration to 0.104 M (because of our routine of buffering with succinate and NaOH, and the presence of $Na^+$ as a complementary ion in some of the essential minerals). Chemicals and nutrients used throughout the experimental series were from a single batch, from a single supplier (Sigma Ltd, unless otherwise stated) and stored in dark, anoxic conditions at 4ºC as liquid stocks. Succinate is a

good buffer for growth at pH 5.8 of *S. cerevisiae* and is not known to be either taken up by, assimilated into or to affect the growth of this species. It will not be an optimal buffer for all microbes or at all pH. Pinning transfers were performed using 384 or 1536 short pin pads (Singer Ltd, UK), and a Singer RoToR HDA robot (Singer Ltd, UK). All transfers were performed using a single robot, standing in the same environmentally controlled space throughout the experimental series and a single production batch of Plus plates and pin pads. The robotic work space was sterilized by prolonged UV exposure before each use and routinely cleaned with ethanol. Three strain layouts were used:

1) All colonies being diploid BY4743 reference strain (Brachmann et al. 1998) (Fig 1-3). The experiment was designed as follows: A lawn of perfectly mixed BY4743 cells was cast by dispersion of 50 µL of an overnight culture evenly across the surface of the solid agar media and incubation at 30ºC for 2 days. From this lawn, a 1536 colony format pre-culture (incubated for 2 days) with SC medium was initiated using short pin 1536 pin pads. Plates were pre-cultured for 2 days, and then re-pinned using short 1536 pins to initiate actual experiments. Cultivations were performed for 3 days at 30ºC.
2) Colonies corresponding to single yeast gene knockouts of the haploid BY4742 deletion collection (Giaever et al. 2002), with BY4742 control colonies interleaved in every fourth position (Fig 4A-D). A liquid pre-pre-culture of deletion strains was constructed by pinning from thawed glycerol stocks with a long pin 96 format pin pad into SC medium (as above). In parallel, a lawn of BY4742 control strains was constructed as above, pinned onto solid media in 384 format and allowed to grow for 2 days. Deletion strains and controls strains were interleaved in a 1536 format (with the 384 short-pin pinning pad) using a custom-made pinning program for the RoToR robot as described in Fig S12. Each thus constructed 1536 format pre-culture plate contained *n*=3 replicates of 384 each mutant strain, with strains in juxtaposition as a consequence of the pinning scheme (Fig S12), and 384 control strains. Plates were pre-cultured for 2 days, and then re-pinned using short pins 1536 format pinning pads to initiate actual experiments. Cultivations were carried out during 3 days at 30ºC.
3) For the confirmation experiment (Fig 4E-F) the same procedure as in 2) was employed, except that *n*=24 replicates of each deletion strains were cultivated, distributed randomly over two experimental plates.

All Epson scanners stand in the same environmental controlled room in which the circulating air is maintained at exactly 30ºC by means of thermostat, and air humidity is maintained above >80% using an air humidifier, to reduce evaporation. Plates are not covered by lids during the experiment but the scanner lid is firmly and evenly placed on top to minimize evaporation. Scanners were covered by boxes (40x60x30cm) with water trays (50mL in a 4.5x12x1cm tray) inside each box in order to avoid cracks emerging in the agar because of drying. For the reference liquid media experiments (Fig 4E-F, S13B-C), 10µL from each well from the same 96 format microtiter plates that were used for the agar experiment was transferred to 350µL SC media per well in a 100-well format Bioscreen C plate. Pre-cultures were incubated for 2 days at 30ºC. 10µL of each pre-culture was then transferred to six experimental 100-well plates (i.e. Honeycomb plates), each well containing 350µL media (media as above but excluding the agar) and incubated in six different Bioscreens ($n$=6) for 3 days, as previously described (Warringer et al. 2011). Each of the six Bioscreen C used were individually temperature controlled to exactly 30ºC, with temperature variation across the plate being less than 0.1ºC. The lid of each plate is heated to slightly above 30ºC, to avoid any condensation on the inside of the lid that would influence the optical readings. All experimental procedures were standardized to the greatest extent possible and the standards strictly adhered to.

## SUPPLEMENTARY FIGURE LEGENDS

### Fig S1. Scan-o-matic process overviews

A) View of the scanner used, Epson Perfection V700 PHOTO scanner (Epson Corporation, UK), with the lid open and an acrylic glass fixture with four slots for plates. B) Overview of the Scan-o-matic hardware – software arrangement and interactions (dotted lines). Three scanners are connected via USB to a single controlling computer. The power supply of each scanner is controlled individually and independently from the computer using a single GEMBIRD EnerGenie PowerManager LAN with multiple sockets (Gembird Ltd, the Netherlands). Experiments on the different scanners connected to one computer are run as separate autonomous processes, while scanner-to-power supply toggling is coordinated by the Scan-o-matic server. This ensures that malfunction in one scanner does not affect the other scanners connected to the same computer. C) Overview of the scanning and the 1$^{st}$ pass analysis. After scanning, the 1$^{st}$ pass analysis identifies the position of the orientation markers within each whole fixture-image and matches recorded positions to positions in a stored model of the used fixture. The match is used to precisely determine positions (the edges) of each of the four plates in the fixture and of the transmissive scale calibration strip in the respective image. The transmissive scale is further analyzed to automatically establish the positions and values of the transmissive scale areas. These values are used to calibrate pixel intensities into opacity values in such a way that these become independent of variations in scanner properties over time and physical environment (temperature, humidity, electrical disturbances), and between scanners. Observe that the 1$^{st}$ pass analysis is performed immediately after plate-images are acquired, image per image, and is completed, reported and stored after acquisition of each image. The experiment processes rests until next scan is expected (standard setting every 20 minutes). D) Overview of the 2$^{nd}$ pass analysis. 2$^{nd}$ pass analysis detects colonies and quantifies their respective cell counts, image per image for each plate. 2$^{nd}$ pass analysis is performed in a time reverse manner, i.e. starting from the last image in each time series, after all images have been acquired. The reason for this is that the precision of gridding will be best on the developed colonies. First, a grid corresponding to the pinning density used is placed on top of each plate-image and positions of grid intersections are precisely aligned with the center point of candidate colonies. Second, each grid intersection region is segmented so that colonies are distinguished from their background:

pixels are assigned to colonies and local backgrounds, respectively. For each colony, the average local background is subtracted from the grey-scale calibrated pixel intensity (opacity value), giving a background subtracted pixel opacity value. This reflects the opacity effect of cells in that pixel. Background subtracted pixel opacity values are automatically converted into cell counts by an empirically defined general calibration function. The complete process of gridding, segmentation, background subtraction and cell number estimates is iterated for all 1536 colonies, in each of the four plates, for each of the images (145 for a 48h experiment with 20 min measurement intervals), starting from the last image is automatic. For one experiment (four plates) this 2$^{nd}$ pass process roughly takes 4 hours.

**Fig S2. Localizing and positioning image orientation markers relative the fixture calibration model**

A) Localizing orientation markers within each individual image. Each image is thresholded with a pixel value of 127 (value range midpoint) resulting in a two-Colour image. The thresholded image is then convolved, using a fast Fourier transformation, with a black and white representation of the fixture orientation marker (upper right corner). This evaluates, for each pixel of the image, how well the local context of that pixel matches a representation of a fixture orientation marker (result shown in the image, dark blue = very unlike a marker, dark red=very like). Iteratively, with the number of iterations corresponding to the number of fixture orientation markers in the fixture model, the strongest signal position is selected and chosen as the marker center position (red pixel in blow-up) and stored. To avoid repeated reporting of the same marker, the local area around a found marker is set to one less than the minimum of the convolution surface. The process of marker localization is repeated on the modified surface until all markers have been found. The mass center of all three markers (red star) is calculated to serve as a reference point during image registration. B) The center point of each marker, is consistently and correctly assigned throughout an experimental series. *Upper panel:* Shows a highly resolved view of an orientation marker from a typical experiment. *x*, *y* coordinates of pixel positions relative its center are given on the axis. Blue line intersection = central pixel, *c*, of the marker according to the algorithm. *Lower panel:* Fraction of images in this experimental time series for which a particular pixel position is

identified as the central pixel of the same marker. Position (0, 0) indicates the most common position. The other positions are given as offsets. The panel shows that for the vast majority of images, the same pixel position is identified as the central pixel, and when other assignments emerge, these are very rarely more than one pixel off in either dimension.

**Fig S3. Calibrating pixel intensities using a calibration strip**

A) Example of how calibration strip areas are identified and their pixel intensities are estimated. The image section corresponding to each calibration strip is sequentially trimmed, first along its width (second panel from the left) and then along its length (third image panel from the left), to exclude all pixels not belonging to the actual calibration strip. The red rectangle indicates the borders of the trimmed area. The 2D center of each calibration strip area (red circles) is then localized (rightmost panel). A representative value of the pixel intensities around each segment center is then taken as the best representation of pixel intensities in that area. B) Illustration of how the edges for the length-wise definitions of the calibration strip area is set. One example calibration strip is shown. First, we calculate the local variance of pixel intensity in regions corresponding to 60% the width and length of the expected grey scale segment sizes. This is done because the part that corresponds to the actual calibration strip positions is expected to be highly uniform in pixel intensities, i.e. have low variation. Comparatively, other features outside the strip will be less uniform. Second, we take the row-wise median of the variances reported by the first step. This is to avoid any spurious local variation peaks, such as due to dust or hair. Blue line = row-wise median of the variances. The permissible rows, i.e. rows that could potentially belong to the calibration strip rather than its surrounding, are heuristically defined as rows with a median of variances below 1000. Dashed red line = cut-off for permissible rows. The edges of the permissible areas (note: there may be more than one permissible area, as shown in the figure) are considered potential edges of the calibration strip area. We define the true calibration strip area as the permissible area that best agrees with the expected length of calibration strip. Red-arrows = called endpoints for the true calibration strip area. Because the variance is calculated across a region, it will begin to increase before the end pixel row of the end segments, causing a systematic underestimation of the true calibration strip area. To avoid loss of parts of the end

segments, and the associated potential skew of the calibration function, we add an extra 75% of the expected length of a single calibration target segment to each end. The final positions for the calibration strip end point definitions are indicated with green arrows. Red circle = noise due to dust or scratches that are accounted for when estimating the representative pixel intensities of each segment. C) Example of a calibration function. The expected values for each calibration strip area (*x*-axis) are provided by the calibration strip producer. Together with the observed calibration strip area pixel intensities (*y*-axis), these are used to construct a third degree polynomial calibration function. The calibration function is specific for each calibration target strip and image. The calibration function is used to translate all observed pixel intensities in an image, ranging from 0-255, to a normalized value space of calibrated pixel opacity values, ranging from 0 to 100. These are used for all further analysis and accounts for systematic variations in pixel intensity values over time and between instruments (see effect on growth curve in Fig 1B). Such variations emerge due to change in properties of the lamp, the sensor and changes to the space between these. D) In a typical experiment in perfect and highly standardized conditions with a new scanner and a new lamp, variations within an experimental time series in observed calibration strip are pixel intensities are small, but detectable. A typical such experiment is displayed. *x*-axis: each of 22 calibration strip areas on a calibration strip. *y*-axis: each of 217 images in one time series (one experiment). Colour: observed pixel intensity, from 0 (dark blue) to 255 (dark red). The slight image-to-image variation in pixel intensities is seen as a vertical banding pattern in each column of intensities.

**Fig S4. Placing a virtual positioning grid across each plate image**

The first step in the 2$^{nd}$ pass analysis places a virtual grid that matches the user supplied pinning matrix over the raw image of each plate and aligns grid intersections with the centers of candidate colonies. A) Raw image of a plate. Blue box indicates edge of the plate included in the image B) Defining a threshold pixel intensity for each pixel individually which is capable of identifying it as a background or a potential colony pixel for the entire plate. The picture shows the threshold for the entire plate in A). Threshold (Otsu thresholds, (Otsu 1975)) values after smoothing indicated with Colours. Dark red = high and dark blue = low pixel intensity being required to call a pixel a colony pixel). The surface is constructed from a

randomly seeded Voronoi diagram. For each partition in the diagram, the pixel intensities of background pixels are estimated to form one Gaussian distribution and the pixel intensities of colony pixels to form another. The Otsu threshold defines the optimal intensity to separate a mixed Gaussian distribution, i.e. to separate the pixels into candidate colony (blob) pixels and background pixels. The surface of threshold-values is heavily smoothed to remove sharp edges between partitions. C) The surface in B) is used to call blob pixels (red) as distinct from local background pixels (blue). Blue box indicates the thin red line of false positives at the bottom of the image that result from the edge of the plate. D) Aggregations of blob pixels are simplified, using iterations of binary erode and binary propagation. This re-assigns some pixels at the blob/background boundary. Blue box indicates the still remaining thin red line of false positives at the bottom of the image that result from the edge of the plate, however it now lacks many of the holes it had in C). E) All blobs are evaluated for size (>40 pixels in area size and cover fewer pixels than the square of the expected distance between colonies). Note that this removes the thin line of false positives at the bottom of the image, indicated by the blue box. F) All remaining blobs are evaluated for shape (expected to be circular due to the shape of the pin head that places the cells on the plate and expected to approximate evenly radiating growth; i.e. bounding box is expected to be roughly square). Blue box: area which is detailed in the zoom-in in H. Note: the shape evaluation could not unambiguously identify the colony in the center of the boxed area as a true colony, leading to its exclusion from the gridding step. G) The average empirical spacing between pins in the used pin format is used to construct an idealized grid. This idealized grid assumes that all pin centers, and consequently the center of all deposited colonies, are an average distance apart. The idealized grid is fitted to the blobs in F such that the sum of errors between grid intersections in the idealized grid and the blob center positions is minimized. Grid intersections of the idealized grid are finally replaced with the nearest blob mass center, given that the nearest blob is close enough (squared distance must be less than a heuristically set threshold of 105 pixels). H) Resolved view of the placed grid in a subsection of the plate, corresponding to the blue box in F. Grid intersections (white circles) and blob centers (light blue crosses) are indicated. Note how the colony removed by the shape selection in F nevertheless is precisely defined using the information provided by its neighbors.

**Fig S5. Assigning pixels to colonies and local background and estimating background subtracted pixel opacity values**

A) A typical example of a colony towards the end of an experiment. B) The picture shows three colonies (1, 2 and 3) and their local surroundings. The chosen areas reflect three challenges that must be successfully resolved for both colony population size and the local background to be correctly estimated. Challenges are: hair (colony 1), specks of dust (colony 2), and distortions in the transparency of the plastic casting (colony 3). The raw pixel intensities (leftmost panels) are first smoothed with a median filter (size 3x3 pixels) to reduce noise (2$^{nd}$ panels from left). The purpose of this smoothing is to adjust pixels values that differ dramatically from their immediate neighbors, specifically: to remove or reduce the impact of specks of dust and thin hairs. Note that this vastly but not completely, removes the impacts of the challenging elements (see colony 1 and 2). Pixels are assigned to blob (3$^{rd}$ panels from left). This is performed using Otsu thresholding (Otsu 1975), i.e. each pixel is assigned as candidate colony/blob pixels depending on a threshold that is specific for the local region. To accurately define blobs and ensure that the edge of each colony is included in the colony's definition, blobs are passed through iterations of binary dilation. This re-assigns some pixels at the boundary of each blob. Blobs falling inside, or partially inside, other blobs, are merged. The largest, most circular blob that, if it's not the first image to be analyzed, best concurs with the blob of the previous iteration (image, analyzed from end to start), is designated as a colony. Other blobs are placed in a second array as trashed pixels. Trashed pixels correspond to specks of dust, scratches on the plastic, or other similar types of noise and are not considered further. Note that this completely removes any remaining influence of the challenging elements encountered (3$^{rd}$ panels from left, blob pixels in red). C) Distribution of pixel opacity values at the grid intersection shown in A) after smoothing. Scan-o-matic assumes that colony pixel opacity values and local background pixel opacity values define two overlapping Gaussian distributions. Pixels are assigned to blob (candidate colony) and local background respectively, based on their pixel opacity values.

**Fig S6. Converting background subtracted pixel opacity values to cell counts**

The transformation from pixel opacity values to actual cell densities was based on a calibration experiment in which 42 colonies with a wide range of sizes were analyzed in Scan-o-matic, according to the procedures described above. Solid media (agar) pieces with each colony on top were then carefully removed from the plate, colonies were washed off from the agar into sterile water by extensive vortexing and the actual number of cells in each colony was estimated using two independent techniques. First, OD600 of diluted samples were measured in a spectrometer (Pharmacia Biotech NovaspecII), estimates were multiplied by the dilution factor and results were transformed into cell density based on that 1 mL of OD = 1.00 medium corresponds to $10^7$ cells. Second, cell samples were sonicated and counted using a Fluorescence Activated Sorting Machine (FACS) (FACS; BD FACSAria). A) The two empirical cell density measures (OD and FACS) showed close to perfect linear correlation, ensuring that they both equally and accurately capture, or at least scales linearly with, real cell numbers in each colony. B) The calibration aims to establish a polynomial function whereby background subtracted pixel opacity values can be converted into actual cell counts per pixel. To establish the polynomial function, we relate the OD based measures of total cells per colony to the corresponding Scan-o-matic based measures of background subtracted pixel opacity values, for all 42 colonies chosen as standards. Note that the Scan-o-matic based measurements are distributions of background subtracted pixel opacity values for each colony. These 42 distributions of background subtracted pixel opacity values are shown, with colonies ordered order from the lowest to the highest total cell count (left to right, top to bottom). These 42 distributions were compared to the 42 corresponding OD measures of total cells per colony to establish the polynomial shown in C. C) OD-measures were used to transform pixel opacity values (calibrated pixel intensity) to cell density, assuming a polynomial relation. The best fit polynomial was used: $y = 1.2678179849224454 \cdot 10^{-4} x^5 + 128.91562154694719x$, for $x > 0$, where $y$ = cell counts and $x$ = background subtracted pixel opacity values. Intermediate exponents are omitted to avoid over-fitting the curve. Pixels with $x < 0$, are set to $x = 0$ before applying the polynomial. D) To validate the accuracy of polynomial transformation across the full range of background subtracted pixel opacity values the 42 colonies were plotted against the observed population sizes. Close to perfect 1:1 correlation (slope, $k = 0.98$; Pearson $r^2 = 0.98$) was observed.

**Fig S7. Reducing noise in growth curves**

To reduce noise in growth curves, we smooth all growth curve in two steps. A) The removal of positive and negative spikes by median filtering, effect depicted in example curve. Such spikes may be due to vibrations, variations in the power flow, dust particles passing by the sensor, or due to fluctuations in properties of the lamp, in particular when it approaches the end of its life span. There is no biological reason for spikes to occur and they must be removed in order not to distort estimates of growth rates. The upstream analysis has several features such as the conversion to pixel opacity values and the image registration with orientation markers that remove the vast majority of the events that would have caused spikes, but the first curve smoothing filter is included as a final safe-guard. We filter for spikes using a median filter of size = 5, i.e. the median of each of five consecutive OD values replaces the third value in each such series of five. The filter's logic implies that if the curve is continuously increasing, as is the typical growth curve scenario, the data will not be altered in any way by the filter. If a measurement stands out in either negative or positive direction it will be replaced by one of its neighbors. For the initial and final measures reflect edge conditions are applied: e.g. for the first measure we estimate the values for the virtual two measures preceding it by assuming they would have been equal to the first and second value in the actual growth data. B) Removal of low amplitude, high frequency noise for two example growth curves. We employ a Gaussian filter of width $\sigma = 1.5$. This allows estimates for all time points surrounding a particular time point to contribute to adjusting its estimate with the degree of each contribution, i.e. its weight, being defined by the distance (in time points). Weights follow a normal distribution centerd on the estimate itself, with the standard deviation of this weight distribution set to 1.5. The Gaussian filter much reduces lower amplitude noise, even if this noise occurs with a high frequency. The high frequency noise in the cases shown is shared across all colonies in one column and derives from the belt of the scanner approaching the end of its life-span, i.e. from a loss of accuracy in the positioning of the lamp at this specific column position. These two smoothing procedures are standard in signal processing and very light, i.e. we are conservative when removing noise, preferring not to risk distorting true biological features such as multiphasic growth. Because both the median and the Gaussian filter are local and very light, they cannot fully account for complex noise that persists over many consecutive time points. This can be seen for the very noisy growth curves in Fig S7B: arrows point towards some cases of complex noise that cannot be completely accounted for. C) Smoothing cannot correct highly distorted growth curve that

results from fundamental problems in the experimental set-up. The distortion shown derives from a badly placed calibration strip. For this calibration strip, pixel intensities for several calibration area segments cannot be correctly estimated. The problem can be compensated for by patching the calibration strip values from an image in a separate experiment to the affected experiment. This circumvents the image to image normalization and will thereby cause the data to be noisier than usual (See Fig 1B upper panel). The patching should therefore only be done when the cost of rerunning the experiment justifies the lower data quality. One affected growth curve is shown, before and after smoothing and after patching with a correctly scanned calibration strip.

**Fig S8. The period of maximal growth rate is short and growth is sometimes complex in expanding yeast colonies**

A) The first derivative of three BY4743 yeast colonies, randomly selected from 1536 genetically identical sister colonies, growing on the same NaCl (1M) and galactose (2%) containing synthetic defined medium surface. The first derivative was extracted after smoothing of population sizes. B) Example of non-standard growth. Growth with three distinct growth phases is shown. Growth curves correspond to biological triplicates of the same strain.

**Fig S9. Effect of pinning format on Scan-o-matic growth curves**

Genetically identical (WT, BY4743) colonies were pinned with either 1536 or 384 pins onto four identical basal medium plates (synthetic defined media) with either 1536 ($n$=2 plates) or 384 pins ($n$=2 plates). Note that variation as a fraction of the signal, or CV, was much smaller for the 1536 (mean CV of 4.9% for 1536 vs. 8.2% for 384) A) Random growth curves from 1536 ($n$=10) and 384 pinned plates respectively ($n$=10). B) Mean population doubling time for each plate. Error bars = SEM ($n$=1536 or 384).

**Fig S10 Normalization by initial population size**

Normalization by initial population size accounts for some but not all of the spatial bias on population doubling time and is inferior to reference grid normalization. Growth phenotypes (colour) for all colonies on one 1536 plate (plate 1 in Fig S11): all colonies are genetically identical BY4743 populations growing on galactose + NaCl medium. A) Population size doubling times ($log_2$), before any normalization. B) Relative population size doubling times, after reference grid normalization. Note that the control position values in this display are removed since and they per definition have the value 0 as a consequence of how the normalization surface is constructed. C) Initial population size D) Normalization surface for population doubling times, constructed based on the distribution of initial population sizes in C. The normalization surface was constructed based on the least square fit of a linear transformation of the initial values in C to the $log_2$ population doubling times in A. It expresses the $log_2$ population doubling times expected in each position, given the initial population size. E) Relative population size doubling times, after normalization to initial population size, i.e. after subtracting the normalization surface in D from the observed values in A. Blue indicates shorter and red longer than expected doubling time.

**Fig S11. Spatial bias before and after reference grid normalization**

Spatial bias is removed by reference grid normalization. Genetically identical reference colonies are pinned into every fourth colony position, creating a matrix of 384 control colonies on which a normalization surface of population doubling times is based. The local normalization surface value is subtracted from each observation. *Left panels*: distributions of population size doubling times of 1536 genetically identical colonies for four plates, before normalization. Each square corresponds to a colony position. *Right panels:* As left panels, but colour represents population size doubling times after reference grid normalization. Plates correspond to distinct environments, but all experiments are genetically identical, thus an even surface is expected. Colour indicates population size doubling time with red = slow growth and blue = fast growth. Colour scales are linear but ranges vary between plates to maximize resolution. Ranges are indicated to the right.

**Fig S12. Construction of a reference grid of controls using a custom designed Scan-o-matic pinning program**

Strains from two source plates, plate A containing intended experiments and plate B containing intended identical reference strains in all positions, are successively transferred to one target plate, C. Pinning is iterated x4 (i - iv) resulting in a 3:1 ratio of experiments relative controls on the target plate. Note that plate C should not be used directly as experiment plate because the spatial bias of control colony growth on this plate poorly reflects the spatial bias of the growth of experiments. Instead, plate C should be used as a pre-culture for the real experimental plate.

**Fig S13. Reference grid normalization with and without removal of deviating controls**

Figure illustrating the effect of removing controls with values that deviate substantially from their neighbors before invoking the normalization. The figure depicts the normalized minimum population doubling times (h) of a single plate of 1536 BY4743 colonies growing on synthetic defined medium. The minimum population doubling times of every fourth colony were used to construct a surface of control values to which the minimum population doubling times of all other colonies were normalized. Colour indicates normalized values of each experiment, i.e. the $\log_2$ difference between each experimental value and the control surface value in that position. Note that in the complete absence of noise and measurement error, all normalized values should equal zero (orange). *Upper panel*: normalized experimental values when including control values that substantially deviate from their neighbors in constructing the normalization surface. *Lower panel*: normalized experimental values when excluding control values that substantially deviate from their neighbors in the construction of the normalization surface. Note (black circles, arrows) that the failure to exclude deviating controls distorts the normalization surface in surrounding positions such that the normalization for these positions will introduce rather than remove error (upper panel: high value bias around the two dark blue control positions). The third dark blue position in the plates is not in a control position and therefore doesn't affect the normalization.

**Fig S14 Scan-o-matic performance of gene deletion strains in absence of stress**

The haploid *MAT***a** yeast deletion collection was cultivated in Scan-o-matic in absence of stress. $Log_2$ population doubling times relative the control surface of WT controls were extracted. Negative values represent growth defects. A) Frequency distributions of salt-specific deletion strain growth effects, obtained by solid substrate cultivation in Scan-o-matic and by liquid microcultivation in a Bioscreen C. B-C) A subset of 70 deletion strains were re-cultivated in absence of stress at high replication, using Scan-o-matic (solid; $n$=24) and liquid ($n$=6) microcultivation respectively. Re-cultivations were performed in parallel, removing all conceivable systematic variation beside cultivation method. B) Growth effects of gene deletions in solid (Scan-o-matic) and liquid microcultivation. Regression (black, Pearson $R^2$ is indicated) and 1:1 lines (red) are shown. C) Gene deletion strains were ranked based on growth effects during solid substrate cultivation and growth effects were plotted. Error bars = SEM.

**Fig S15 Comparing Scan-o-matic and previously published yeast growth data**

The figure shows pair-wise comparisons of gene deletion growth phenotypes between studies, for basal conditions and NaCl rich environments. Colour: Spearman rank correlation (scale at right). Note that some studies are done using liquid and some using solid microcultivation and that studies differ in the type and amount of carbon, nitrogen, sulphur, mineral and vitamin compounds present, pH, buffering of the media, degree of shaking, temperature, humidity, presence/absence restrict oxygen and carbon dioxide flow, ploidy, marker composition, initial inoculum size, physiological state of precultures, and cultivation time. For NaCl rich media, NaCl concentrations differ. Finally, the manner in which growth data is recorded, treated, and reported differs substantially, with regards to whether complete or sub-fractions of populations are measured, whether colony area or population size is measured, whether data reflects the total yield, integral of the growth area, population doubling times or specific growth rates, and what time points that have been used to extract these numbers. To compensate for that some phenotypes would be expected to be inversely correlated (e.g. 1/h to h), we decided to multiply each data series with 1 or -1, such that they yielded the most number of positive correlations to all other studies. Because the differences in methods and

phenotypes measured don't readily allow for the expectancy of correlation linearity even under ideal circumstances, Spearman's rank correlation coefficient (Spearman's rho) was used rather than Pearson product-moment correlation coefficient. Experimental studies compared were: (Baryshnikova et al. 2010; Warringer et al. 2003; Hartman and Tippery 2004; Hughes et al. 2000; Jasnos and Korona 2007; Brauer et al. 2008; Yoshikawa et al. 2009). The correlations show the expected low to moderate degree of monotonic correlation. In general studies tend to correlate more to other results in the same study than to more comparable media compositions in other studies. Note that this causes the studies represented by several replicates to have seemingly higher correlations in general. The present Scan-o-matic results correlate with about equal magnitude to the other studies as Warringer 2003 - in most aspects the most comparable study. The trend of correlations for the present study generally follows the expectancies: when comparing two studies, it has increased correlation when more similar media conditions are compared (e.g. Zackrisson NaCl correlates better to Yoshikawa NaCl than to Yoshikawa Basal, while Zackrisson Basal correlates best with Yoshikawa Basal).

**Fig S16. Spatial bias in the form of local changes in pH across solid media plates**

Cells secrete organic acids metabolic by-products of carbon metabolism, lowering the pH of the local environment. Secreted acids diffuse slowly through the media, creating systematic spatial variations in pH as a function of colony size and colony metabolic state. External pH affects growth through altering a wide range of molecular phenotypes. Figure shows a time resolved view of pH change across a plate as a function of time. A plate was cast with unbuffered SC medium (initial pH = 6.0) supplemented with a pH indicator (1mg/50mL bromocresol green) and seeded with genetically identical BY4743 colonies (*his3Δ::kanMX4*) at uneven initial population sizes. Intense yellow = pH below 3.8, intense blue = pH above 5.4. Note: we recommend users to buffer the medium. Buffering to pH 5.8 was employed in all other experiments reported in this article.

**Supplementary movies**

**File 2 and 3. Animations connecting raw colony images to growth**

Animations of horizontal colony area and volume as a function of time. Two random colonies are shown. Time is given in hours, with images acquired every 20 minutes over a 72h period. File 2) *Left panel*: Raw colony images. Axes show pixel positions in the vertical (*y*) and horizontal (*x*) dimensions. Pixel intensity shows original pixel intensity values: white = low intensity (background), black = high intensity. *Right panel*: Non-smoothed growth curve showing the total change in population size (cells) in the colony (*y*-axis) as a function of time (*x*-axis). File 3) *Left panel*: Colony images. Axes show pixel positions in the vertical (*y*) and horizontal (*x*) dimensions. Pixel intensity shows calibrated pixel opacity values: dark blue = low intensity (background), light blue = high intensity. *Middle panel*: Rotating 3D rendering of the colony, showing its change in all three dimensions over time (height is slightly exaggerated estimate based on image resolution and spherical cells). *Right panel*: Smoothed growth curve showing the total change in population size (cells) in the colony (*y*-axis) as a function of time (*x*-axis).

**File 4. Animation illustrating spatial bias in population size as a function of time**

Animation showing the smoothed population size (colour intensity) for 1536 genetically identical WT (B74743) colonies growing on a 2% glucose + 0.85M NaCl plate. Red = low population size (25000 cells), blue = high population size (4.25 million cells).

## REFERENCES


Baryshnikova, A., M. Costanzo, Y. Kim, H. Ding, J. Koh *et al.*, 2010 Quantitative analysis of fitness and genetic interactions in yeast on a genome scale. *Nature methods* 7 (12):1017--1024.

Blomberg, A., 2011 Measuring growth rate in high-throughput growth phenotyping. *Curr Opin Biotechnol* 22 (1):94-102.

Brachmann, C.B., A. Davies, G.J. Cost, E. Caputo, J. Li *et al.*, 1998 Designer deletion strains derived from Saccharomyces cerevisiae S288C: a useful set of strains and plasmids for PCR-mediated gene disruption and other applications. *Yeast* 14 (2):115-132.

Brauer, M.J., C. Huttenhower, E.M. Airoldi, R. Rosenstein, J.C. Matese *et al.*, 2008 Coordination of growth rate, cell cycle, stress response, and metabolic activity in yeast. *Mol Biol Cell* 19 (1):352-367.

Giaever, G., A.M. Chu, L. Ni, C. Connelly, L. Riles *et al.*, 2002 Functional profiling of the Saccharomyces cerevisiae genome. *Nature* 418 (6896):387-391.

Hartman, J.L., and N.P. Tippery, 2004 Systematic quantification of gene interactions by phenotypic array analysis. *Genome biology* 5 (7):R49.

Hughes, T.R., C.J. Roberts, H. Dai, A.R. Jones, M.R. Meyer *et al.*, 2000 Widespread aneuploidy revealed by DNA microarray expression profiling. *Nat Genet* 25 (3):333-337.

Jasnos, L., and R. Korona, 2007 Epistatic buffering of fitness loss in yeast double deletion strains. *Nat Genet* 39 (4):550-554.

Mosberger, D., 1998 The SANE Scanner Interface. *Linux J.* 1998 (47es).

Otsu, N., 1975 A threshold selection method from gray-level histograms. *Automatica* 11 (285-296):23-27.

Ottosson, L.G., K. Logg, S. Ibstedt, P. Sunnerhagen, M. Kall *et al.*, 2010 Sulfate assimilation mediates tellurite reduction and toxicity in Saccharomyces cerevisiae. *Eukaryot Cell* 9 (10):1635-1647.

Pylvänäinen, I., 2005 A Parametric Approach to Yeast Growth Curve Estimation and Standardization in *Department of Mathematical Sciences*. Chalmers University of Technology, Gothenburg, Sweden.

van der Walt, S., S.C. Colbert, and G. Varoquaux, 2011 The NumPy Array: A Structure for Efficient Numerical Computation. *Computing in Science & Engineering* 13 (2):22-30.

van der Walt, S., J.L. Schonberger, J. Nunez-Iglesias, F. Boulogne, J.D. Warner *et al.*, 2014 scikit-image: image processing in Python. *PeerJ* 2:e453.

Warringer, J., and A. Blomberg, 2003 Automated screening in environmental arrays allows analysis of quantitative phenotypic profiles in Saccharomyces cerevisiae. *Yeast* 20 (1):53-67.

Warringer, J., E. Ericson, L. Fernandez, O. Nerman, and A. Blomberg, 2003 High-resolution yeast phenomics resolves different physiological features in the saline response. *Proc Natl Acad Sci U S A* 100 (26):15724--15729.

Warringer, J., E. Zorgo, F.A. Cubillos, A. Zia, A. Gjuvsland *et al.*, 2011 Trait variation in yeast is defined by population history. *PLoS Genet* 7 (6):e1002111.

Yoshikawa, K., T. Tanaka, C. Furusawa, K. Nagahisa, T. Hirasawa *et al.*, 2009 Comprehensive phenotypic analysis for identification of genes affecting growth under ethanol stress in Saccharomyces cerevisiae. *FEMS Yeast Res* 9 (1):32-44.
